# Early cephalopod evolution clarified through Bayesian phylogenetic inference

**DOI:** 10.1186/s12915-022-01284-5

**Published:** 2022-04-14

**Authors:** Alexander Pohle, Björn Kröger, Rachel C. M. Warnock, Andy H. King, David H. Evans, Martina Aubrechtová, Marcela Cichowolski, Xiang Fang, Christian Klug

**Affiliations:** 1 Paläontologisches Institut und Museum, Universität Zürich, Karl-Schmid-Strasse 4, CH-8006 Zürich, Switzerland; 2 Finnish Museum of Natural History, University of Helsinki, P.O. Box 44, Jyrängöntie 2, FI-00014 Helsinki, Finland; 3 GeoZentrum Nordbayern, Friedrich-Alexander Universität Erlangen-Nürnberg, Loewenichstrasse 28, 91054 Erlangen, Germany; 4 Geckoella Ltd, Suite 323, 7 Bridge Street, Taunton, TA1 1TG UK; 5 Natural England, Rivers House, East Quay, Bridgwater, TA6 4YS UK; 6 Institute of Geology and Palaeontology, Faculty of Science, Charles University, Albertov 6, 12843 Prague, Czech Republic; 7 Institute of Geology, Czech Academy of Sciences, Rozvojová 269, 16500 Prague, Czech Republic; 8 Instituto de Estudios Andinos “Don Pablo Groeber”, CONICET and Facultad de Ciencias Exactas y Naturales, Universidad de Buenos Aires, Ciudad Universitaria, Pab. 2, C1428EGA Buenos Aires, Argentina; 9 State Key Laboratory of Palaeobiology and Stratigraphy, Nanjing Institute of Geology and Palaeontology and Center for Excellence in Life and Paleoenvironment, Chinese Academy of Sciences, 39 East Beijing Road, Nanjing, 210008 China

**Keywords:** Cephalopoda, Phylogeny, Nautiloidea, Orthoceratoidea, Multiceratoidea, Endoceratoidea, Bayesian phylogenetics, Fossilized birth-death process, Posterior clade probabilities, Tree similarities

## Abstract

**Background:**

Despite the excellent fossil record of cephalopods, their early evolution is poorly understood. Different, partly incompatible phylogenetic hypotheses have been proposed in the past, which reflected individual author’s opinions on the importance of certain characters but were not based on thorough cladistic analyses. At the same time, methods of phylogenetic inference have undergone substantial improvements. For fossil datasets, which typically only include morphological data, Bayesian inference and in particular the introduction of the fossilized birth-death model have opened new possibilities. Nevertheless, many tree topologies recovered from these new methods reflect large uncertainties, which have led to discussions on how to best summarize the information contained in the posterior set of trees.

**Results:**

We present a large, newly compiled morphological character matrix of Cambrian and Ordovician cephalopods to conduct a comprehensive phylogenetic analysis and resolve existing controversies. Our results recover three major monophyletic groups, which correspond to the previously recognized Endoceratoidea, Multiceratoidea, and Orthoceratoidea, though comprising slightly different taxa. In addition, many Cambrian and Early Ordovician representatives of the Ellesmerocerida and Plectronocerida were recovered near the root. The Ellesmerocerida is para- and polyphyletic, with some of its members recovered among the Multiceratoidea and early Endoceratoidea. These relationships are robust against modifications of the dataset. While our trees initially seem to reflect large uncertainties, these are mainly a consequence of the way clade support is measured. We show that clade posterior probabilities and tree similarity metrics often underestimate congruence between trees, especially if wildcard taxa are involved.

**Conclusions:**

Our results provide important insights into the earliest evolution of cephalopods and clarify evolutionary pathways. We provide a classification scheme that is based on a robust phylogenetic analysis. Moreover, we provide some general insights on the application of Bayesian phylogenetic inference on morphological datasets. We support earlier findings that quartet similarity metrics should be preferred over the Robinson-Foulds distance when higher-level phylogenetic relationships are of interest and propose that using a posteriori pruned maximum clade credibility trees help in assessing support for phylogenetic relationships among a set of relevant taxa, because they provide clade support values that better reflect the phylogenetic signal.

**Supplementary Information:**

The online version contains supplementary material available at 10.1186/s12915-022-01284-5.

## Background

The tree of life is fundamental to understanding processes in evolutionary biology. Reconstructing different parts of the tree is therefore a major goal in this research area [1]. Today, evolutionary trees are most commonly reconstructed using DNA data from living representatives of the group of interest. However, in the case of long-extinct clades, morphological data are the only available source for phylogenetic inference. The inclusion of fossil representatives is nevertheless important because they can significantly alter tree topologies and provide the main source of evidence to calibrate evolutionary timelines [2–6]. The analysis of morphological data sets presents a major challenge because they are inherently more complex than molecular data and probabilistic models of character evolution have only recently begun to be employed more frequently [7]. An important recent development is the fossilized birth-death (FBD) model, which directly incorporates the diversification and fossil recovery processes. Furthermore, it accounts for sampled ancestors, i.e., sampled species can give rise to descendant species in the tree, instead of being restricted to sampled leaves [8–10]. Several studies have highlighted the impact of accounting for sampled ancestors on divergence time inferences [10–12].

In Bayesian phylogenetic inference, different model assumptions and their impact on tree topology or other parameters of interest can be tested. Most commonly, these model assessments are done using Bayes factors and stepping-stone analyses [13]. However, although studies have shown that the data selection (i.e., taxa and characters) can significantly impact analyses under the FBD model [14, 15], objective criteria for selecting different sets of taxa or characters are rarely employed. Model testing can only be used to compare different models applied to the same data, but not if different subsets of the data are used to generate the results. Comparing the implications of data selection, such as the inclusion of key taxa or different interpretations of certain characters, is thus challenging. The currently best solution to this problem is to compare the effects of different data sets on the parameters of interest. For example, if the main interest of a study is tree topology, it is possible to compare the resulting trees via commonly used tree distance or similarity metrics [14]. The first question we address in this study is, therefore, how different modifications to the main dataset affect the results. Intuitively, one would assume that the more data the better; however, in many large clades, it is not practical to sample all available data.

Among the results of a Bayesian phylogenetic inference is usually a large number of trees, i.e., the posterior set of trees. Multiple approaches to reconstruct summary trees from the posterior have been developed, the most popular being the maximum clade credibility (MCC) and the majority-rule-consensus (MRC) trees [16]. In both approaches, the support of a clade is measured by its posterior probability, i.e., the proportion of trees in the posterior sample that contain the same clade. Several studies advocated that MRC trees should generally be preferred because MCC trees may contain spurious clades that are poorly supported by the data [17–19]. However, low posterior probabilities are typical for many trees containing fossil data [13, 20, 21]. MRC trees therefore have the potential to provide limited information about tree topology, from a systematic perspective, if uncertainty is high. Additionally, MCC trees often approximately conform to phylogenetic hypotheses despite low clade supports and similar trees are recovered frequently from maximum parsimony analyses [2, 22]. One possible reason for low posterior probabilities are taxa that have unstable positions across optimal trees. For example, if a true clade contains 50 species, then every time even a single species is missing, the clade is considered to be unsupported, thus decreasing posterior probability.

The terms “wildcard taxa” or “rogues” have been introduced for taxa whose phylogenetic position is highly unstable [23, 24]. Their detrimental effect has long been recognized, leading to their frequent exclusion in bootstrap analyses under the maximum parsimony optimality criterion [23]. However, it is not trivial to identify wildcard taxa, because large amounts of missing data do not necessarily imply topological instability and even highly complete taxa can act as wildcards if they carry unusual character state combinations [25]. Nevertheless, several approaches have been developed to detect wildcard taxa [26–29]. Studies that use Bayesian inference do not often include specific treatment of wildcard taxa, despite their potential impacts. The treatment of potentially problematic taxa is typically their removal prior to the main analyses, which does not inform the range of topological uncertainty of these particular taxa. Furthermore, this practice omits phylogenetic information, as these character combinations are not “noise”, but rather real observations that fit somewhere into the context of the tree. An additional question we address with this study is therefore, whether excluding potential wildcard taxa candidates (e.g., those with high amounts of missing data or early representatives) a priori improves posterior clade probabilities. Alternatively, what happens if wildcard taxa are pruned a posteriori from the tree sample?

Cephalopods are an excellent test group for the above questions because of their outstanding fossil record. The overwhelming majority of recent cephalopods belong to the Coleoidea, which includes octopus, squid, and cuttlefish. In contrast, the genera *Nautilus* and *Allonautilus* are the only living representatives of the externally shelled Nautiloidea. Based on molecular evidence, the divergence between recent coleoids and nautiloids has been placed in the Silurian or Devonian periods [30–34]. However, most of these studies show high uncertainties in divergence dates, which is perhaps caused by the small number of closely related crown group nautiloids that share a very recent divergence date [35]. Accordingly, divergence dates within the Cephalopoda rely heavily on fossil calibrations of molecular clocks, which come with their own pitfalls [36, 37]. In addition, the phylogeny of many extinct groups of cephalopods is unclear, which complicates assignment of potential calibration points to modern lineages. To better understand the evolutionary history of this clade, it is therefore crucial to resolve relationships among early fossil representatives of the Cephalopoda.

Classically, cephalopods have been divided into three subclasses: the abovementioned Coleoidea and Nautiloidea, and the exclusively fossil Ammonoidea [38]. The Nautiloidea has often been subdivided into further subclasses, although no consensus exists and members of all these groups are still informally called “nautiloids” [39]. While the phylogenetic relationship between the monophyletic Coleoidea and Ammonoidea is well established [40], the Nautiloidea is paraphyletic. This is well illustrated by the fact that it contains taxa that fall within crown group cephalopods, but also many taxa that are clearly part of the stem group [32], although the precise extents of stem and crown group are unclear. Different, partly incompatible, phylogenetic hypotheses for nautiloid cephalopods have been proposed, but none has received unanimous support [39]. The common feature of all previous hypotheses is that they were not the result of a phylogenetic analysis but were reconstructed rather by intuitive comparison of taxa and their stratigraphic positions. Thus, it may not be surprising that different conclusions were reached depending on the emphasis that was given to certain characters. With a few exceptions, the classification at order level has remained relatively constant since the first attempt of a phylogenetic classification by Flower and Kummel [41]. The phylogeny used by the Treatise of Invertebrate Paleontology [42] did not differ markedly from the former hypothesis, while Dzik [38] proposed a very different phylogeny and was subsequently criticized [43, 44]. His approach consisted of drawing pictograms representing intraspecific variation of each species and arranging them intuitively along the time axis, with the main criterion of morphological continuity, explicitly disregarding their previous systematic position. One of the main problems with his classification was that he drastically reduced the number of species and taxa by applying a very loose species concept, which is not followed by most recent studies. Furthermore, because he did not employ any algorithm to arrange his taxa, his results remain subjective. Most recently, Mutvei [45, 46] and King and Evans [39] proposed new phylogenetic relationships and corresponding classifications, again without any accompanying phylogenetic analyses.

Interestingly, Dzik ([38] p. 12) stated “Phylogenetic trees are most commonly reconstructed intuitively or numerically […]. The adequacy of numerical methods may actually be doubtful”. In comparison with today, numeric methods may have been limited at the time his monograph was published. Modern methods for phylogenetic inference based on morphological characters have massively improved since then and are widely used, especially in vertebrate paleontology and are currently gaining more popularity for invertebrate groups [20, 21, 47, 48]. Many aspects of numerical inference are undoubtedly preferable to “intuitive” phylogenetic classifications, including their repeatability, their testability, and their use of explicit character weights.

**Fig. 1:**
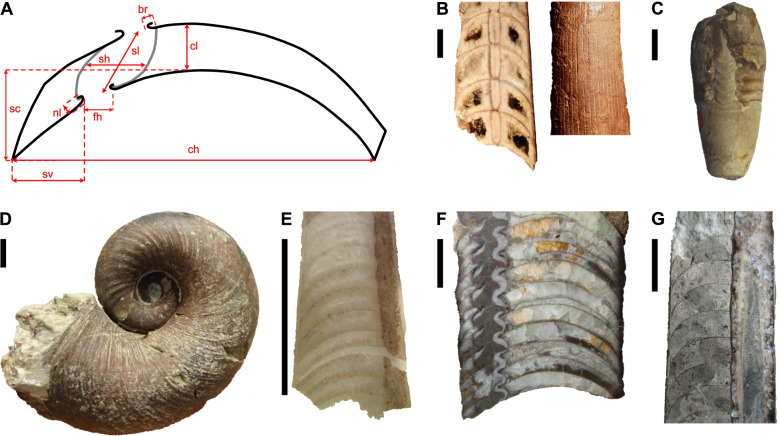
Measurements and examples of specimens. **A** Measurements taken from longitudinal sections. Abbreviations: ch = conch height, sv = siphuncle position, fh = septal foramen height, sh = segment height, sl = segment length, cl = cameral length, sc = septal concavity, nl = septal neck length, br = brim height. **B*** Polygrammoceras lineatum*, Orthocerida, NRM Mo 3100. **C ***Pictetoceras eichwaldi*, Ellesmerocerida (Multiceratoidea), GIT 805-2. **D ***Charactoceras kallholnense*, “Barrandeocerida”, NRM Mo 8735. **E ***Novacaroceras endogastrum*, early Ellesmerocerida, NIGP 73824. **F*** Pseudowutinoceras wuhaiense*, Actinocerida, NIGP 54244. **G*** Dideroceras longispiculum*, Endocerida, NRM Mo 158235. Institutional abbreviations: GIT = Department of Geology, Tallinn University of Technology, Estonia; NIGP = Nanjing Institute of Geology and Palaeontology, China; NRM = Naturhistoriska Riiksmuset, Stockholm, Sweden. Scale bars = 1 cm

Here, we present an attempt to test the phylogenetic relationships between early cephalopods using the fossilized birth-death model based on a large, entirely new character matrix. We restricted our analyses to Cambrian and Ordovician cephalopods, because of the large number of described species (more than 3000 in the Ordovician period alone, [49]) and the fact that except for the Nautilida, all nautiloid orders appeared during this time interval, indicating an important rapid initial diversification [38, 41, 42]. The matrix contains representatives from all major groups of this diverse clade (Fig. 1). Note that new endings for the names of orders and subclasses were recently proposed by King and Evans [39]. This matter requires a thorough discussion elsewhere, and here we use the traditional endings as introduced in the old Treatise of Invertebrate Paleontology Part K [42]. However, we want to highlight that this is subject to change with the upcoming revision of the Treatise Part K [39].

## Results

**Fig. 2:**
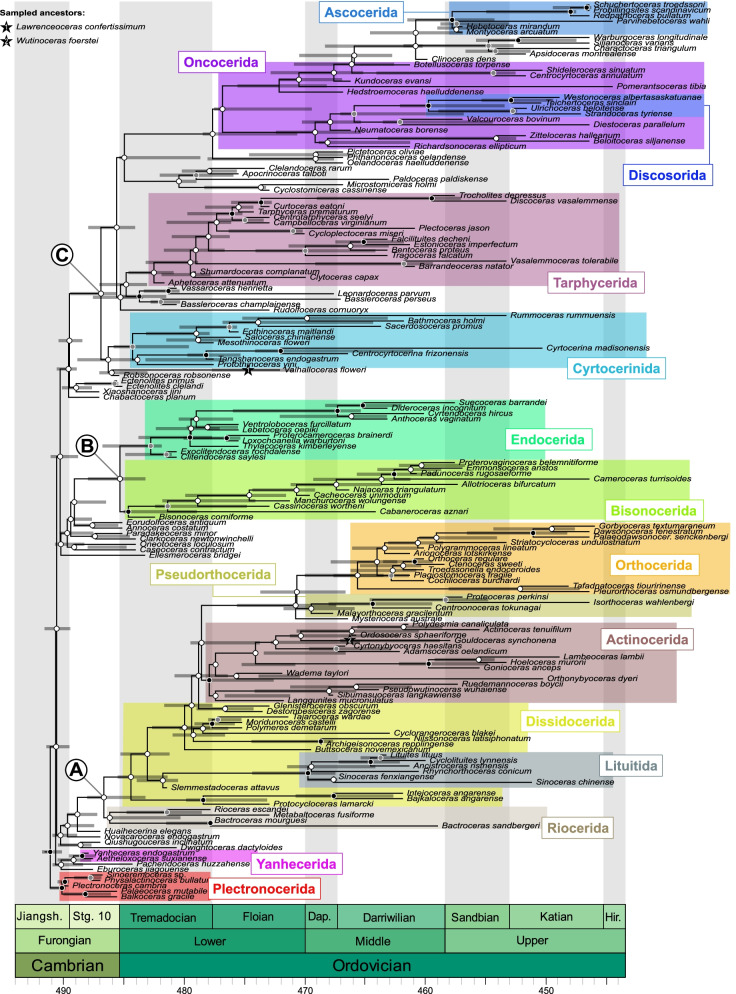
Full maximum clade credibility tree inferred from the main dataset (CtCo). The three major clades correspond to the Orthoceratoidea (A), Endoceratoidea (B), and Multiceratoidea (C). Clade posterior probabilities are shown as circles at nodes, black indicating posterior probability > 0.75, gray between 0.5 and 0.75, and white < 0.5. Colored boxes correspond to established taxonomic groups. Taxa outside these groups mostly belong to the Ellesmerocerida, but also contain some species with uncertain affinities (e.g., members of the Apocrinoceratidae, Uranoceratidae). Note that missing character scorings of some species were complemented with characters from congeneric species. In these cases, OTUs technically correspond to genera. See “Methods” and Additional file 2: Data S1 for details

**Fig. 3:**
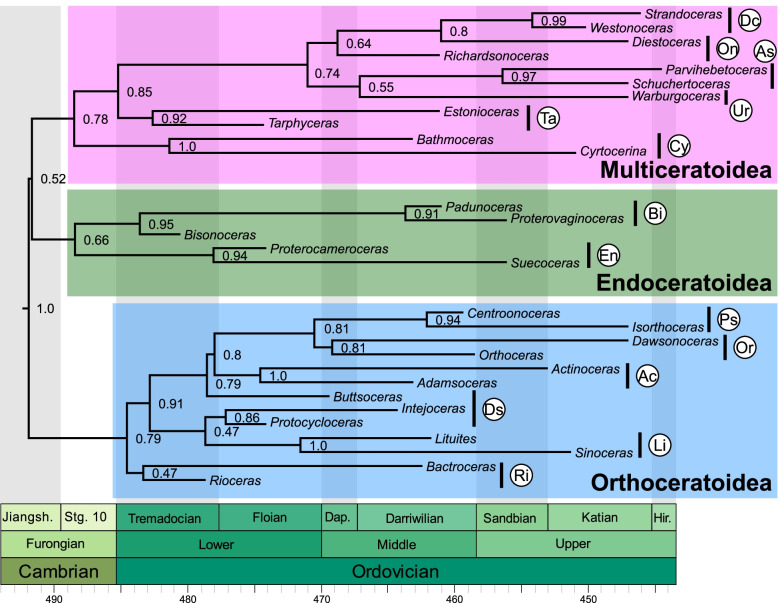
Pruned maximum clade credibility tree. The posterior tree sample was produced using the main dataset (CtCo). For most groups, only one or two representatives were retained, and 144 taxa were pruned from the tree sample. Numbers shown at nodes represent posterior probabilities. Abbreviations: Ac = Actinocerida; As = Ascocerida; Bi = Bisonocerida; Cy = Cyrtocerinida; Dc = Discosorida; Ds = Dissidocerida; En = Endocerida; Li = Lituitida; On = Oncocerida; Or = Orthocerida; Ps = Pseudorthocerida; Ri = Riocerida; Ta = Tarphycerida; Ur = Uranoceratidae

We compiled a comprehensive list of 141 characters with hierarchical relationships including detailed explanations and measurements (see Fig. 1, Additional file 1: Text S1, Figs. S1-S5, Tables S1 and S2). These characters were scored for a total of 173 species of Cambrian and Ordovician cephalopods. We ran 12 different analyses with slightly modified data sets (Table 1). We use MCC trees to report the results instead of MRC trees, as the latter would be uninformative due to a large number of polytomies; furthermore, the MCC trees contain similar topologies across different iterations, suggesting that the phylogenetic signal is consistent, even if weak. The tree topology resulting from the main analysis (CtCo) is shown in Fig. 2, for further trees see Additional file 1: Figs. S6-S17. The tree in Fig. 2 was produced using all species and excluding several controversial characters and thus corresponds to the preferred topology. Most of the section on tree topology refers to this tree. We produced another set of MCC trees by retaining only one or two taxa per order from the same posterior tree sample as the full MCC trees (Fig. 3, Additional file 1: Figs. S18-S23). For these pruned MCC trees, we also excluded all plectronocerids, yanhecerids, and ellesmerocerids, so that they did not obscure clade support values of broader scale relationships between the more nested clades due to their tendency to attach to early branches of any of these clades. This provided a clearer picture about which clades may be regarded as well supported, and those for which alternative relationships must be considered. To compare the impact of excluding these taxa a priori, we conducted another analysis with the same taxa as in the pruned MCC tree of the main dataset (Additional file 1: Fig. S24). We will first report on parameter estimates and uncertainties reflected by the trees, before going into more detail on tree topology. See “Methods” for additional details.

**Table 1:** Overview of analyzed datasets

Dataset	Character criteria	# characters	Taxa criteria	# taxa
CtCo	Controversial characters excluded	135	All taxa	173
CoCo	All characters	141	All taxa	173
IcCo	Characters with > 75% missing data and controversial characters excluded	112	All taxa	173
AmCo	Autapomorphies and controversial characters excluded	122	All taxa	173
IaCo	Characters with > 25% gaps (= inapplicable) and controversial characters excluded	74	All taxa	173
CrCo	Controversial characters excluded and speculatively scored connecting ring type	135	All taxa	173
MaCo	Controversial characters excluded and speculatively scored muscle attachment scars	135	All taxa	173
CMCo	Controversial characters excluded and combined speculatively scored characters	135	All taxa	173
CtDp	Controversial characters excluded	135	Pseudoduplicate taxa excluded	169
CtIc	Controversial characters excluded	135	Taxa with > 40% missing data excluded	135
CtEl	Controversial characters excluded	135	Most ellesmerocerids and Cambrian taxa excluded, except for a few basic morphotypes	140
CtRd	Controversial characters excluded	135	Randomly selected 50% of taxa excluded	87

Criteria for exclusion or inclusion of characters and taxa are listed. Details on which taxa and characters were excluded are listed in Additional file 1

### Parameter estimates

**Fig. 4:**
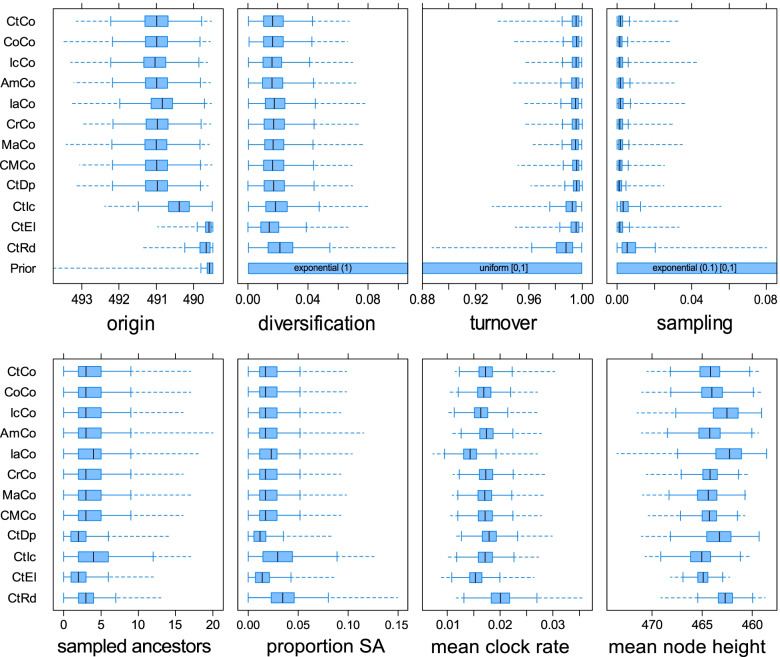
Parameter estimates. As a comparison, the prior distributions of the origin, diversification, turnover, and sampling parameters are shown. Values in brackets denote mean values, while square brackets represent the lower and upper limits of the distributions. The younger origin estimates produced by some datasets are likely a result of too little information content. All other datasets show relatively little influence on parameter estimates. Abbreviations for datasets are shown in Table 1. SA = sampled ancestors

All analyses resulted in comparable parameter estimates that differ from their prior distributions (Fig. 4). Apart from tree topology, different sets of characters appear to have little to no influence on the main parameters of the FBD model. Species selection had a modest effect when their number was decreased (CtIc, CtEl, CtRd; > 30 species excluded), but this mainly resulted in broader ranges of the estimates, indicating more uncertainty. Note that these runs had species removed according to different criteria, and it is thus unclear whether the increased uncertainty was caused by the decrease in species number or the effect of the exclusion criteria. The only exception was the origin parameter, where decreased species sampling resulted in younger age estimates, potentially caused by removing older taxa from the dataset, as the datasets in question—CtIc, CtEl, and CtRd—all involved removing several Cambrian taxa. In any case, the difference was relatively minor (approximately 1 my).

The number of sampled ancestors was comparable and relatively low in all runs. The removal of pseudoduplicates (CtDp) and ellesmerocerids (CtEl) resulted in lower relative numbers of sampled ancestors, possibly because many of the removed taxa are likely candidates to be recovered as sampled ancestors due to their stratigraphic position and similarity with younger taxa.

Divergence time estimates tended to favor relatively old divergence dates, suggesting that the three major clades and even some of the subclades had already diversified during the late Cambrian, considerably prior to their appearance in the fossil record. Mean node heights varied between analyses, with the datasets excluding characters with high proportions of incomplete and inapplicable characters (IcCo and IaCo) or excluding pseudoduplicate or half of the taxa (CtDp and CtRd) recovering on average 2.5 my younger divergence dates, though variation within analyses was up to approximately 10 my. However, as our main focus was on tree topology, divergence dates should be regarded with caution, as further model testing and the inclusion of occurrence data may influence these estimates.

### Posterior probabilities

**Fig. 5:**
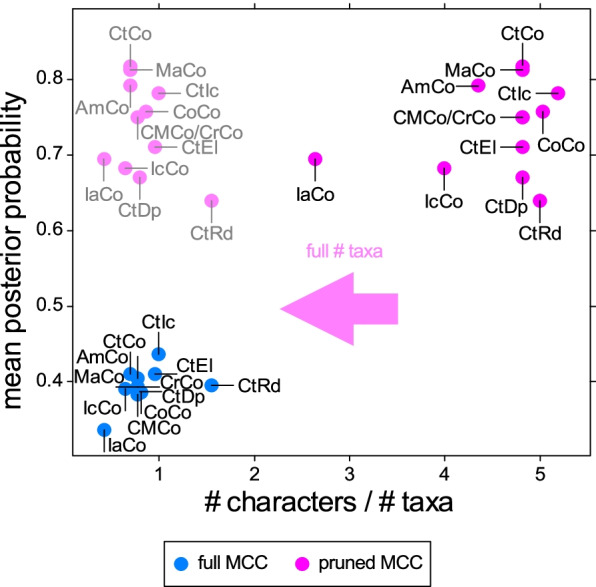
Mean clade posterior probabilities. The mean posterior probability of the MCC trees resulting from each analysis is compared to the proportion of characters to taxa. Transparent dots represent the original number of taxa in the dataset, before pruning the MCC trees. Abbreviations for datasets are shown in Table 1

The posterior probabilities of clades were generally low. When mean clade posterior probabilities of MCC trees from different analyses were compared, there was a trend towards higher mean posterior probabilities when the ratio between characters and species was high (Fig. 5). In other words, lower numbers of species and higher numbers of characters improved clade support. However, note that the pruned MCC trees were constructed by a posteriori pruning of trees generated using a larger number of species. In the full MCC trees, excluding half of the tips from the analyses did result in a slightly lower mean clade support compared to the main analysis, despite increase an in the proportion between characters and species. Excluding incomplete species had the most positive effect, while excluding characters with > 25% inapplicable scorings resulted in the lowest mean clade support. For pruned MCC trees, much higher clade supports were retrieved. The highest mean clade support among the pruned MCC trees was retrieved when controversial characters were excluded, while the exclusion of random tips resulted in the lowest mean clade support.

**Fig. 6:**
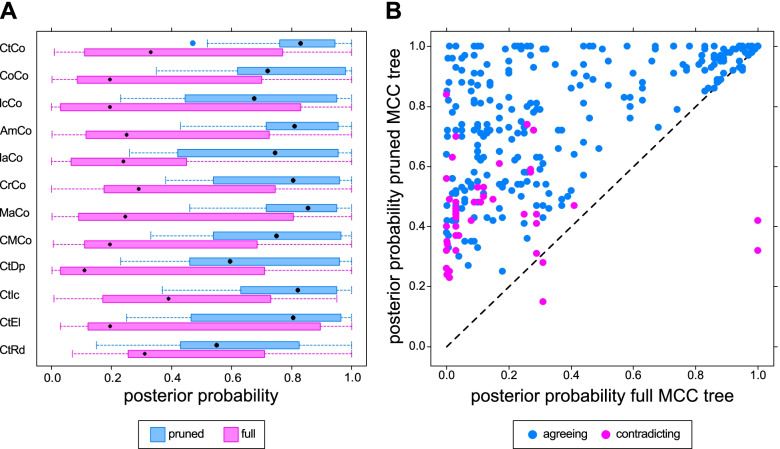
Comparison between equivalent nodes in pruned and full MCC trees. Equivalent nodes are defined as encompassing the minimal clade that contains the same taxa. **A** Distribution of posterior probabilities in each dataset. **B** Direct comparison of all equivalent nodes regardless of dataset. Contradicting nodes contained in full MCC trees taxa that were recovered outside this clade in the corresponding pruned MCC tree. The dashed line represents equal posterior probabilities in full and pruned MCC trees

Equivalent nodes in the pruned MCC trees (i.e., the smallest possible clade in the full MCC tree that includes the same taxa as in the pruned MCC tree) had higher posterior probabilities on average by 0.35 when compared to the full MCC trees (Fig. 6A; see also Additional file 1: Table S3). Some clades reconstructed in the pruned MCC trees contradicted clades contained in the full MCC trees—this mainly happened in clades that had low support (Fig. 6B). Note that the direct comparison of equivalent nodes is impeded in that they may carry different taxonomical meanings. For example, the clade comprising *Adamsoceras* and *Actinoceras* encompasses the entirety of actinocerids in the pruned MCC tree but denotes an internal node within the Actinocerida in the full MCC tree. In addition, contradicting nodes may define very different clades, when two taxa are resolved as sister taxa in the pruned MCC tree but recovered as polyphyletic in the full MCC tree.

In the MCC tree from the analysis that used the same taxa as in the pruned MCC tree of the main analysis (Additional file 1: Fig. S24), posterior probabilities were on average at 0.64, thus higher than in all full MCC trees, but lower than in all pruned MCC trees except for the analysis excluding half of the taxa CtRd (Additional file 1: Table S4). Note that the clades recovered in this tree differ from those retrieved in the pruned MCC tree of the main analysis.

### Tree comparisons

**Fig. 7:**
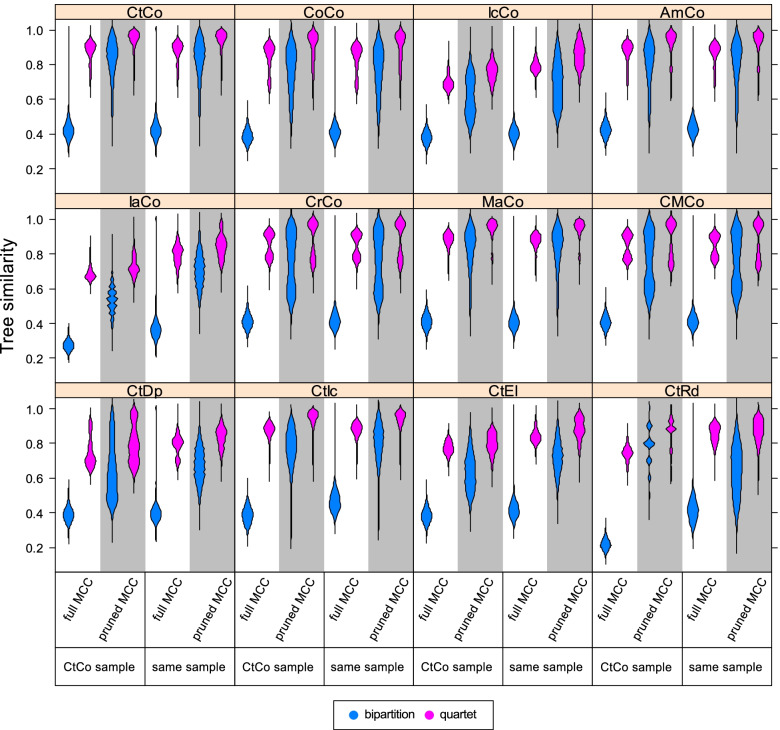
Distributions of tree comparisons. Each set of posterior trees is compared to four different single trees: the full and the pruned MCC tree of the main analysis and the full and the pruned MCC tree resulting from the same posterior tree sample. Comparisons are made with bipartition (blue) and quartet (pink) similarity. Abbreviations for datasets are shown in Table 1. Different datasets generally result in similar trees, bipartition returns lower similarities than the quartet metric and pruned MCC trees exhibit higher similarities to the posterior sample than full MCC trees. Note that while the mathematically possible range is the same for both metrics, the expected mean values for the comparison between two random trees are 0.0 for the bipartition metric, and $$\raisebox{1ex}{$1$}\!\left/ \!\raisebox{-1ex}{$3$}\right.$$ for the quartet metric [22]. See Additional file 1: Table S4 for rescaled quartet similarities

While clade posterior probabilities represent support for a given clade within the same analysis, tree metrics can be used to compare differences between trees (Fig. 7, Additional file 1: Table S4). The results can be summarized as follows: (1) Datasets that were altered by only a small extent resulted in the most similar trees. Excluding considerable amounts of characters or species led to more dissimilar trees being recovered (note that only species present in both trees were considered; see “Methods”). MCC trees that were on average more dissimilar to other MCC trees also showed a wider distribution when they were compared to the corresponding set of posterior trees. This suggests that these trees reflect a higher degree of uncertainty due to a larger number of conflicting tree topologies being recovered. (2) Bipartition similarities were consistently lower than quartet similarities, although this difference was less extreme in the pruned MCC trees. This also applied when quartet similarities were rescaled so that the expected similarity between two random trees had a value of 0.0 as opposed to $$\raisebox{1ex}{$1$}\!\left/ \!\raisebox{-1ex}{$3$}\right.$$ as in unscaled quartet similarities [50]. (3) Pruned MCC trees generally had higher tree similarities than full MCC trees. (4) Comparing a tree or a tree sample to the MCC trees yielded similar results. In other words, the full MCC trees were about as equally similar to each other as to the posterior tree samples. (5) When taxa were pruned a priori, this resulted in much lower similarity values, and particularly quartet similarity was consistently lower for every dataset. Only the IaCo and CtRd full MCC trees had even lower bipartition similarities (Additional file 1: Table S4).

### Leaf stability

**Fig. 8:**
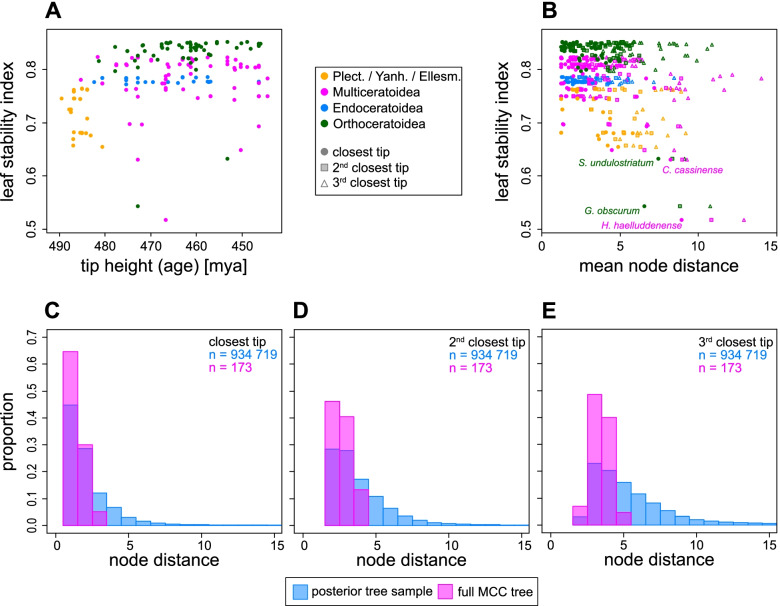
Leaf stability and node distance. All graphs refer to the CtCo analysis and its full MCC tree. **A** Leaf stability index compared to tip height. Note the clade and age dependence. **B** Leaf stability index compared to the mean node distances among the tree posterior of each taxon to its three closest neighbors in the full MCC tree. The four most unstable taxa are labeled. **C** Distribution of node distances to the closest neighbor of each taxon in the full MCC tree compared to the distribution of the node distances between the same taxa among the entire tree posterior. **D** Same, but with the 2nd closest tips. **E** Same, but with the 3rd closest tips

To assess the impact of wildcard taxa, we calculated the leaf stability index [24] of all taxa in the full MCC tree of the main analysis, which shows the dependency of this index to clades and age (Fig. 8A). Older taxa, in particular those belonging to either Plectronocerida, Yanhecerida, or the paraphyletic Ellesmerocerida (see below), had lower leaf stabilities. While the Endoceratoidea and, with two exceptions, also the Orthoceratoidea had relatively narrow distributions of leaf stabilities, the distribution was wider in the Multiceratoidea, with some representatives receiving particularly low scores, e.g., *Hedstroemoceras haelluddenense* with 0.52 or *Cyclostomiceras cassinense* with 0.63 (Fig. 8A, B; Additional file 4: Data S3). We also investigated the average amount of instability of all taxa by calculating node distances of all taxa to their three closest neighboring tips in the MCC tree and comparing these to the node distances of the same taxa in all posterior trees. Although leaf stability and the mean node distance behaved in a similar way, there were also taxa that had low leaf stability but moved only slightly between neighboring nodes and vice versa (Fig. 8B). The distribution of node distances of the closest tips in the full MCC tree deviated only slightly from the distribution of the distances between the same taxa in the entire posterior tree sample (Fig. 8C–E). Importantly, although node distances went up to 43 in a few extreme cases, 95% of the node distances were ≤ 6 for the closest tip, ≤ 8 for the 2nd closest tip, and ≤ 13 for the 3rd closest tip. This indicates that although the topological positions of the taxa were variable, this was mostly restricted within the same region of the tree.

### General tree topology

Three major clades can be identified in the full MCC tree of the main analysis, as well as in the MCC trees based on the other datasets. The interrelationships and composition of those clades vary between analyses and their posterior probabilities are consistently low. However, as we demonstrate above, the trees are more congruent than the posterior probabilities initially suggest. The three clades correspond to the previously defined subclasses Endoceratoidea, Multiceratoidea, and Orthoceratoidea [39, 42, 46]. Note that the name Multiceratoidea is preferred here in favor of Nautiloidea, because we did not include any Nautilida in our dataset and their origin is still debated [32, 39]. Furthermore, the extent of the Multiceratoidea is close to their original definition [46]. It might be practical to restrict the Nautiloidea to the order Nautilida until their ancestral lineage is identified (either the Multiceratoidea or the Orthoceratoidea). Naturally, this would make their ancestral group paraphyletic. The Orthoceratoidea are already paraphyletic because they contain the presumable ancestors of both ammonoids and coleoids [32]. Sister group relationships between endoceratoids, orthoceratoids, and multiceratoids are challenging to resolve and we did not recover a stable topology, although most analyses weakly supported a sister group relationship between Endoceratoidea and Multiceratoidea, which together are the sister group to the Orthoceratoidea. In addition, we recovered a large, mostly paraphyletic assemblage of taxa near the base of the tree in the Cambrian and Early Ordovician. These nodes near the root also reflect the highest degree of uncertainty. This may be due to the fact that Cambrian cephalopods are comparatively poorly known and in many cases studied from longitudinal thin sections only [51, 52]. Furthermore, many early representatives lack specialized structures such as endosiphuncular or cameral deposits, resulting in a considerable number of characters that were scored as inapplicable and thus effectively a decreased number of informative characters when compared with later taxa. Accordingly, the similar character state distributions of these early taxa may suggest that the topological uncertainty among early taxa is at least in part caused by stochastic variation in the analyses rather than representing a genuine evolutionary pattern.

Below, we compare the MCC trees recovered from different analyses, with an emphasis on the analysis that excluded several controversial characters (Fig. 2, S6, CtCo; see “Methods” for details). We report the posterior probabilities (= PP) for the MCC tree recovered from the latter analysis and refer to additional files of MCC trees from other analyses that show alternative topologies (Additional file 1: Figs. S7-S17).

### Late Cambrian and Early Ordovician cephalopods

The Plectronocerida contains some of the oldest known cephalopods [51]. Based on investigations of original material, we consider the Protactinocerida to be synonymous with the Plectronocerida and perceived differences between them are mainly due to oblique sections [52, 53]. We recovered this group either as a monophyletic sister clade to the remaining cephalopods (PP = 0.77; Fig. 2, Additional file 1: Figs. S6-S8, S11-S12, S14), or as a paraphyletic group at the base of the cephalopod tree (Additional file 1: Figs. S9, S13 and S15). The Plectronocerida are traditionally interpreted as the ancestral group of cephalopods, among others, due to their stratigraphic position [32, 44]. Recent claims that plectronocerids are a derived clade and descended from small ellesmerocerids are mostly based on the premise that the plectronocerid siphuncle is more complex and therefore cannot represent the ancestral state [53]. However, complexity alone is not a good indicator of ancestor-descendant relationships. In fact, others have argued the reverse that the plectronocerid siphuncle must be the ancestral state because of its unique structure [44, 54]. Although some trees recovered individual ellesmerocerids in a position as sister group to all other taxa (Additional file 1: Figs. S10, S17), this was recovered only from the analyses excluding inapplicable characters or removing half of the taxa. Deciding whether ellesmerocerids evolved from plectronocerids or vice versa will require ancestral state reconstructions in future studies, although the stratigraphic position currently slightly favors the plectronocerids as ancestral.

The Yanhecerida is a small group of Cambrian cephalopods that was only represented by *Yanheceras* and *Aetheloxoceras* in our analyses. Although our results suggest that the two genera form a monophyletic group (PP = 0.82), it is not clear whether it warrants giving the group the status of an order. The topology is likely to be driven by the conical shape of their diaphragms. However, the three-dimensional structure of diaphragms is poorly studied, and effects of preservation and preparation may cause differences to ellesmerocerids that seem more apparent than real. Generally, yanhecerids appear to be more closely related to ellesmerocerids, contradicting earlier held views that perceived them as more closely related to the Plectronocerida [39], but confirming others that regarded them as originating from ellesmerocerids [51] or even promoted its synonymy with the latter [53].

Cambrian Ellesmerocerida were highly unstable, and therefore, no conclusions about their phylogenetic relationships can be made here other than they likely represent a paraphyletic assemblage of the ancestral lineage leading to Ordovician cephalopods. Our analyses suggest that all post-Cambrian cephalopods are derived from just a few lineages. Multiple families of Cambrian ellesmerocerids have been proposed [51], but similarly to the Yanhecerida, our dataset included only few representatives of most families, and therefore, we cannot comment on the validity of those groups. One pattern consistent to all analyses was that the divergence between the three major clades took place prior to the Ordovician.

The Ellesmeroceratidae are probably the family with the highest number of genera among early Paleozoic cephalopods. As with the order, this is due to its usage as a waste basket taxon for early cephalopods thought to be “primitive.” The phylogenetic position of many taxa assigned to this family is highly unstable on the tree topology, occupying positions near the root and attaching to any of the nested clades, although some consistent, albeit weakly supported, patterns were recovered. Short breviconic taxa such as *Clarkoceras* or *Caseoceras* were frequently placed at the base of the Endoceratoidea clade (PP = 0.01; Fig. 2, Additional file 1: Figs. S6–S16), while taxa with more slender conchs like *Ectenolites* were placed close to the root of the Multiceratoidea (PP = 0.007; Fig. 2, Additional file 1: Figs. S6, S9, S11, S12). In contrast, the Orthoceratoidea was in most cases directly derived from Cambrian taxa such as *Novacaroceras* (PP = 0.004; but see Additional file 1: Figs. S8, S10, S14). Nevertheless, *Ectenolites* and *Novacaroceras* are so similar that this result may be spurious and a consequence of the large uncertainties, as evident from the low posterior probabilities.

The Bassleroceratidae has long been suspected to be ancestral to the Oncocerida and Tarphycerida [41]. Our results tend to confirm this view, although the situation is similar as in the Ellesmerocerida in that the family represents a paraphyletic group with at least two descendant lineages. There appear to be two separate groups that were consistently recovered somewhere near the base of the Multiceratoidea clade. A close relationship between *Bassleroceras*, *Vassaroceras*, and *Leonardoceras*—which share a compressed conch cross section—was retrieved in all analyses (PP = 0.88), while *Lawrenceoceras* was associated with *Valhalloceras* (PP = 0.24), both of which are characterized by a depressed conch cross section. *Robsonoceras* may also belong to this group, as it was mostly recovered near the base of the entire clade containing the Multiceratoidea and the Bassleroceratidae (PP = 0.28), except in cases where the Multiceratoidea was polyphyletic due to an alternative position of the Cyrtocerinida (Additional file 1: Figs. S10, S17). *Rudolfoceras* was more unstable, but often recovered in a more derived position near the base of the Oncocerida or close to the Cyclostomiceratidae (Additional file 1: Figs. S7, S8, S10, S11, S12, S13, S15). No analysis recovered a close relationship between *Dwightoceras* and other bassleroceratids, although its affinities were also generally unstable. In most analyses, the Bassleroceratidae were recovered in a nested position within the Multiceratoidea, as they were placed within the large sister group to the Cyrtocerinida, although with low posterior probability (PP = 0.08; Fig. 2, Additional file 1: Figs. S6-S9, S11-S16). Consequently, if the Bassleroceratidae are kept within the Ellesmerocerida, the latter would be polyphyletic.

The Cyclostomiceratidae is another group of ellesmerocerids that can probably be assigned to the multiceratoid lineage, but their exact relationships remain obscure. Although several datasets recovered the cyclostomiceratids *Cyclostomiceras*, *Microstomiceras*, and *Pictetoceras* together with *Phthanoncoceras* and *Oelandoceras* as a monophyletic group within the Multiceratoidea (Additional file 1: Figs. S7, S11, S13, S14), other subsets did not support a monophyletic Cyclostomiceratidae. In the main analysis, the family was paraphyletic and ancestral to the Oncocerida/Discosorida/Ascocerida clade (Fig. 2), but in other analyses, cyclostomiceratids were distributed between two different branches (Additional file 1: Figs. S8, S9, S12, S15 and S16). In the first case, the Cyclostomiceratidae were related to the Oncocerida and Discosorida, while they were closer to the Cyrtocerinida in the second case. Because of the high degree of uncertainty, which may partly be caused by the low leaf stability of *Cyclostomiceras*, it is not possible to conclude whether the Cyclostomiceratidae is monophyletic or which taxon is their closest relative, but they belonged to the Multiceratoidea in each MCC tree.

### Orthoceratoidea

This taxonomic unit predominantly encompasses forms with straight conchs, though they are not limited to this group. Furthermore, many orthoceratoids possess cameral and endosiphuncular deposits, although both characters are absent in the earliest members. The subclass has already been accepted by different authors in the past, although opinions on its extent varied [39, 55, 56]. Our results suggest that the Orthoceratoidea are a monophyletic group (when their post-Ordovician descendants such as ammonoids and coleoids are ignored) containing the groups traditionally recognized as the Riocerida, Dissidocerida, Lituitida, Orthocerida, Pseudorthocerida, and Actinocerida (PP = 0.29).

The recently established Riocerida [39] were recovered as sister group to all other orthoceratoids. Our dataset included only a few species of riocerids, but several conclusions can be drawn from the recovered tree topologies: Except for *Pachendoceras*, all riocerids consistently showed up near the base of the orthoceratoid clade. Additionally, the poorly known *Metabaltoceras* was recovered within riocerids. Nevertheless, resolving relationships near the orthoceratoid root is challenging. Although riocerids were monophyletic in our main and most other analyses (PP = 0.37; Fig. 2, Additional file 1: Figs. S6, S7, S11, S13, S15), several datasets suggested that they may be a paraphyletic assemblage of early species that were ancestral to other orthoceratoids (Additional file 1: Figs. S9, S12). In some cases, the Riocerida was polyphyletic, with *Rioceras* recovered as sister taxon to endoceratoids (Additional file 1: Figs. S8, S14 and S16) or even cyrtocerinids (Additional file 1: Fig. S10). However, one observation is that cameral and annular endosiphuncular deposits potentially had a single common origin within the Orthoceratoidea, as these character states were highly prevalent in taxa within the sister group to the deposit-free Riocerida (PP = 0.41). Alternatively, it is also possible that they were secondarily lost, at least in some riocerids, if taxa attributed to the Riocerida are paraphyletic or cameral deposits evolved before at least some of the taxa branched off from the rest of the Orthoceratoidea. At least for orthocerids, it is known that reduction and restriction of cameral and endosiphuncular deposits to only the earliest ontogenetic stages is common [57], thus implying that their presence do not necessarily represent irreversible character states. To better understand the evolutionary distribution of cameral and endosiphuncular deposits within the Orthoceratoidea, further analyses involving ancestral state reconstructions are required.

The Dissidocerida evolved from the Riocerida as the first orthoceratoids that developed cameral and endosiphuncular deposits. Accordingly, they are paraphyletic and ancestral with respect to all subsequent groups of orthoceratoids. This is in agreement with the stratigraphic sequence of appearance and also with the established phylogenetic hypothesis [39]. Remarkably, dissidocerids have some of the most complex endosiphuncular deposits, as many members of the group possess different combinations of diaphragms, annuli, rods, and linings [58]. Gains and losses of cameral and endosiphuncular deposits are difficult to evaluate within early orthoceratoids, especially considering the heterochronous growth of the deposits, which means that differentiating between their absence or restriction to apical (i.e., juvenile) parts of the conch is challenging. While the total group of the Dissidocerida was recovered as paraphyletic in all analyses, two distinct monophyletic groups could be repeatedly identified. The first consisted of *Protocycloceras*, *Intejoceras*, and *Bajkaloceras*, which formed an early diverging branch of the Dissidocerida (PP = 0.84). This confirms that the latter two genera do not belong to the Endoceratoidea and that the Intejocerida is polyphyletic [39]. The second monophyletic dissidocerid clade contained *Tajaroceras*, *Moridunoceras*, *Polymeres*, *Cyclorangeroceras*, *Archigeisonoceras*, and *Nilssonoceras* (PP = 0.03). The latter two genera belong to the Geisonoceratidae that were previously considered as members of the Orthocerida [59], but our results suggest that this family may have evolved independently from the Dissidocerida. Because *Troedssonella* was consistently recovered within the Orthocerida and *Buttsoceras* ancestral to the second dissidocerid monophylum (which contains *Tajaroceras* and *Moridunoceras*), the Troedssonellidae is polyphyletic. Other dissidocerids were recovered in various positions along the dissidocerid branch. Notably, *Glenisteroceras* and *Destombesiceras* were recovered as sister group to the clade containing actinocerids, orthocerids, and pseudorthocerids (PP = 0.47), suggesting that they are not discosorids as originally thought [60]. However, *Glenisteroceras* had a relatively low leaf stability index and was in some analyses recovered together with *Apocrinoceras* and *Paldoceras* (Additional file 1: Figs. S10, S12, S16).

One of the monophyletic groups with the highest posterior support within the Orthoceratoidea was the Lituitida (PP = 0.95). Earlier hypotheses placed the group within the Tarphycerida [41, 42], but the relationship of the Lituitida to the Orthoceratoidea has become clearer in recent years [38, 61, 62], and is also supported by our model. One difference to previous hypotheses is that lituitids were suspected to be descendants of the Orthocerida; however, our tree topologies suggest that lituitids branched off earlier than orthocerids, thus deriving either from riocerids or dissidocerids. Even though our analyses are inconclusive in this regard, we prefer the latter interpretation on the basis of the presence of cameral deposits in all lituitids. This is also in line with the interpretation that the first Orthocerida sensu stricto appeared in the Middle Ordovician (e.g., *Malayorthoceras gracilentum*), approximately contemporaneous to the oldest lituitids (e.g., *Sinoceras fenxiangense* or *Rhynchorthoceras* aff. *beyrichi*, [38, 63]). An alternative tree topology suggested the lituitids as a sister group to the family Orthoceratidae. However, this relationship was only recovered in cases with incompletely scored characters removed (Additional file 1: Fig. S8). Lituitids possess some unique peculiarities and a better understanding of those structures (epichoanitic deposits and the possible *syn-vivo* destruction of the connecting ring [64];) may help clarifying their phylogenetic position within the Orthoceratoidea.

The Orthocerida (PP = 0.24) was recovered as monophyletic in the majority of our analyses, often with the Pseudorthocerida as its sister group (PP = 0.29). However, excluding incomplete characters resulted in a tree topology that divided the Orthocerida into two separate independent lineages (Additional file 1: Fig. S8). In this analysis, several members of the Orthoceratidae were recovered as a sister group to the Lituitida (see above), while the remaining orthocerids comprising *Dawsonoceras*, *Polygrammoceras*, *Malayorthoceras*, and others remained in the same position, as sister group to the Pseudorthocerida. Thus, we cannot rule out a closer relationship between Orthoceratidae and Lituitida. Surprisingly, although all analyses recovered *Striatocycloceras* within the Orthocerida, it was one of the most unstable taxa of the entire Orthoceratoidea, which may be due to its relatively large amount of missing data and its isolated stratigraphic position in the Sandbian, which contrasts with the larger numbers of orthoceratids in our sample from the Darriwilian and the Katian.

Although our analyses confirm a monophyletic Pseudorthocerida as sister group to the Orthocerida (PP = 0.81, when excluding *Malayorthoceras*), several taxa were recovered elsewhere, and the order therefore requires an emendation and a reduction in its scope: First, the hypothesis that the Greenlandoceratidae represent the oldest pseudorthocerids was rejected by our analysis [65]. Instead, taxa such as *Sibumasuoceras* and *Langgunites* were recovered as early representatives of the Actinocerida (PP = 0.75). Furthermore, *Clinoceras* was recovered as ancestral to the Ascocerida, indicating that it belongs to the Multiceratoidea. Lastly, our results suggest that *Gorbyoceras* belongs to the Dawsonoceratidae of the Orthocerida instead of being related to the Proteoceratidae. The remaining pseudorthocerids formed a small, but consistent, monophyletic group consisting of *Mysterioceras*, *Centroonoceras*, *Proteoceras*, and *Isorthoceras*. We thus accept the Pseudorthocerida in this reduced extent. Nevertheless, note that we did not include *Pseudorthoceras* or related taxa in our analysis, because they are restricted to younger stratigraphic positions. It is therefore impossible to establish based on our analyses, whether this lineage originates from what is considered here to be the Pseudorthocerida.

The last clade within the Orthoceratoidea is the Actinocerida (PP = 0.75). Already the data collection process revealed that the traditional view on the evolution of this group is not entirely accurate. Early researchers were puzzled by the fact that *Polydesmia*, which they assumed to be the oldest actinocerid, exhibits such a complex, apparently derived siphuncular morphology [66]. Newer studies showed that the corresponding formations are Darriwilian in age [67]. Instead, *Wadema* from the Floian of Australia appears to be the oldest actinocerid, although the age of the corresponding Coolibah Formation is somewhat uncertain [68]. We emphasize the updated stratigraphic position of these taxa here, considering how deeply entrenched the putative ancestral position of *Polydesmia* is in the older literature [42, 66]. The fixation on this genus was perhaps what prevented earlier attempts to identify the ancestral lineage of the Actinocerida. All early attempts to resolve actinocerid relationships relative to other cephalopods had in common that they assumed their ancestors were within the Ellesmerocerida, and a closer relationship to other orthoceratoids was not considered at that time. More recent studies highlighted the similarities between actinocerids and orthocerids, including cameral and endosiphuncular deposits, connecting ring structure and muscle attachment scars [38, 69]. The inclusion of the Actinocerida within the Orthoceratoidea was also supported by our results. We recovered a monophyletic lineage, containing *Sibumasuoceras*, *Langgunites*, *Pseudowutinoceras*, and *Ruedemannoceras* (PP = 0.18), as sister group to the remaining actinocerid clade (PP = 0.45), which contradicts earlier views that assigned the former taxa to the Pseudorthocerida and Discosorida, respectively [65, 70]. Actinocerids have been proposed to be the sister group to the Pseudorthocerida in one of the few small-scale cladistic analyses on Paleozoic cephalopods [71]. We recovered a slightly different topology, with the Actinocerida as sister group to the Orthocerida and the restricted Pseudorthocerida (PP = 0.26). The reality of this relationship depends on whether the Ordovician and the Carboniferous pseudorthocerids belong to the same lineage, and the same applies to the actinocerids. Although the posterior probability was high for the entire actinocerid clade, relationships within the order were less stable, but a monophyletic Gonioceratidae received high support (PP = 0.99).

### Endoceratoidea

Members of this group are famous for their regionally great abundance in Ordovician rocks and because of their large size [72]. While the Endocerida/Endoceratoidea were recognized early on as forming a distinct taxonomic unit, there have been recurring debates as to whether they represent a monophyletic group or two independently evolved lineages [38, 72, 73]. Our results present a mixed picture, on the one hand supporting two separate co-evolving lineages, while on the other hand suggesting that these lineages form a single monophyletic group (PP = 0.36). There is some uncertainty near the root of this clade, and therefore, it is currently impossible to tell whether the characteristic endocones evolved twice independently or whether one type of endocones evolved from the other—this depends on whether endocone-lacking “ellesmerocerids” fall inside the Endocerida or Bisonocerida clade, or are basal to the entire Endoceratoidea clade. We recovered an alternative topology when we excluded taxa with high amounts of missing data (Additional file 1: Fig. S15). In this case, only a single lineage of endoceratoids was supported, thereby invalidating the Bisonocerida. However, this tree topology is likely driven by the exclusion of a large part of the bisonocerids, which are often known from very fragmentary material. The remaining bisonocerid taxa were separated by significant stratigraphical gaps, which decreased the likelihood of recovering them as a monophyletic group and instead moved younger bisonocerids closer to homeomorphic endocerids with smaller stratigraphic distances. This highlights the importance of including even incompletely known species, as the bisonocerids were not the most notorious wildcard taxa, despite their incompleteness.

Several taxa previously assigned to the Ellesmerocerida (*Lebetoceras*, *Loxochoanella*, and *Ventroloboceras*) were consistently recovered within the Endocerida, suggesting that they are early representatives of this clade that either reduced endocones or that endocones of these species may be found in the future.

Disregarding possible early “ellesmerocerid” members of both clades, our analyses support a sister group relationship between the Endocerida (PP = 0.65) and the Bisonocerida (PP = 0.89) [73]. Bisonocerids are mostly known from fragmentary, isolated siphuncles. Despite the consequentially large amount of missing data, the bisonocerids were consistently recovered as monophyletic sister group of the Endocerida. Although this confirms the Bisonocerida as a clade separate from the Endocerida, it does not make the Endoceratoidea polyphyletic as suggested [73]. Instead, both lineages may include early “ellesmerocerid” members, and thus, these taxa would have to be included in the Endoceratoidea. It is notable that one of these species was *Clarkoceras*, which was proposed already to be ancestral to the Bisonocerida [73]. Future studies are necessary to clarify the early evolution within this subclass.

### Multiceratoidea

This clade (PP = 0.28) contains a large group of diverse orders with variable conch shapes and siphuncular structures. Besides several ellesmerocerids (mainly Bassleroceratidae, see above), this group encompassed the Cyrtocerinida, Tarphycerida, Oncocerida, Discosorida, and the Ascocerida. This is very similar to the original definition of the Multiceratoidea [46], with the only exception that the group was defined to include the entire Ellesmerocerida. However, the latter group is paraphyletic or even polyphyletic, and its members are distributed along the ancestral lineages of the Orthoceratoidea, Endoceratoidea, and Multiceratoidea.

The monophyly of the Cyrtocerinida is well supported (PP = 0.73), although their assignment to the Multiceratoidea is somewhat uncertain. While this topology was recovered by most analyses, there were also some alternative topologies that placed the Cyrtocerinida closer to the Endoceratoidea (Additional file 1: Figs. S10 and S17). In any case, the Cyrtocerinida occupied a position somewhere between endoceratoids and early multiceratoids, not too far from the most recent common ancestor of both clades. Internally, the results appear to confirm two lineages, which roughly correspond to the Cyrtocerinidae (PP = 0.46) and the Eothinoceratidae (PP = 0.41), with *Bathmoceras* as the only representative of the Bathmoceratidae evolving from the Eothinoceratidae. This fits quite well with previous ideas on cyrtocerinid evolution [74]. *Rummoceras*, previously assigned to the Cyrtocerinidae was an exception, as it was more unstable and attached to either lineage depending on the dataset.

The Tarphycerida formed a monophyletic group together with several taxa that were originally classified under the Barrandeocerida, such as the Barrandeoceratidae and the Plectoceratidae (PP = 0.46). The Barrandeocerida is polyphyletic, and we support earlier suggestions to abandon the order and to include those taxa in the Tarphycerida [39], with the exception of the Uranoceratidae and the Apsidoceratidae, which were recovered as sister group to the Ascocerida on the Oncocerida branch (PP = 0.55; Fig. 2, Additional file 1: Figs. S6, S7, S9, S11-S15). In comparison with tarphycerids, the latter taxa differ in tending towards a subtriangular conch cross section with a broad venter and narrow dorsum, while also possessing expanded siphuncular segments. This confirms that fully coiled cephalopods appeared multiple times independently during the Ordovician (in addition to the orthoceratoid *Cyclolituites*). The inferred origin of the Tarphycerida confirms previous hypotheses in that they were recovered as sister group to the Oncocerida with the Bassleroceratidae as ancestral group [41]. Within the Tarphycerida, the Estonioceratidae formed a consistent monophyly (PP = 0.66), as well as a clade containing the Tarphyceratidae and the Trocholitidae (PP = 0.90).

The Oncocerida were recovered as descendants of the Bassleroceratidae and closely related to the Tarphycerida, also confirming previous hypotheses [41]. The Oncocerida themselves were paraphyletic, but formed a monophyletic clade together with the Discosorida, Ascocerida, and the aforementioned non-tarphycerid Barrandeocerida (PP = 0.05). However, many oncocerids had rather unstable positions, with several unusual topologies that sometimes placed a mix of oncocerids and other taxa with uncertain affinities somewhere within the Multiceratoidea and in rare cases even within the Orthoceratoidea (Additional file 1: Fig. S10). The reasons for this are unclear, but it is perhaps related to the rather incomplete species sampling, especially when early oncocerids are considered. In addition, many early members of the Oncocerida such as the graciloceratids have relatively unspecialized conch morphologies, and the resemblance to other taxa with thin, empty, and ventral siphuncles is relatively close. Nevertheless, all analyses recovered a consistent monophyletic group consisting of a (paraphyletic) subset of oncocerids and the descendant Discosorida (PP = 0.41). This subset consisted of *Neumatoceras*, *Richardsonoceras*, *Beloitoceras*, *Zitteloceras*, *Valcouroceras*, and *Diestoceras*. The affinities of oncocerids will have to be re-evaluated using a revised set of characters and taxa.

In the past, various taxa were assigned to the Discosorida, which we did not recover as a monophyletic group. However, Late Ordovician forms that share so-called bullettes, which characterize the Discosorida sensu stricto, consistently formed a monophyletic group derived from the Oncocerida (PP = 0.95). This clade includes *Strandoceras*, *Ulrichoceras*, *Teichertoceras*, and *Westonoceras*. The Apocrinoceratidae, consisting of *Apocrinoceras*, *Paldoceras*, and the poorly known *Clelandoceras*? *rarum* (but not *Glenisteroceras* and *Destombesiceras*), were in some analyses recovered as a monophyletic group (Additional file 1: Figs. S7, S8, S12-S15). In other cases, these three taxa were recovered as a paraphyletic group with various positions within the Multiceratoidea, either within the sister group to the Oncocerida-Tarphycerida clade (Additional file 1: Figs. S7, S9) or diverging from the Oncocerida after their split with the Tarphycerida (Fig. 2, Additional file 1: Figs. S6, S11). This means that the inclusion of the Apocrinoceratidae within the Discosorida is doubtful and requires additional investigations. Lastly, several other taxa which have been assigned to the Discosorida in the past need to be removed from that order and instead transferred to the Actinocerida or Pseudorthocerida. *Ruedemannoceras* was once seen as the earliest discosorid, representing a link between the Plectronocerida and the Discosorida [70]. However, investigation of original specimens of plectronocerids revealed that the “siphuncular bulb” is fundamentally different from what is seen in *Ruedemannoceras* and such a relationship is not supported by the morphological data. In addition, similarities in the connecting ring between *Ruedemannoceras* and Discosorida sensu stricto are likely taphonomical artifacts [39, 75]. Therefore, the genus is better placed within the Orthoceratoidea, where it was recovered as an actinocerid. The same applies to *Gouldoceras* and *Pseudowutinoceras*, which have been suspected to be discosorids on different occasions [76, 77]. Although the exact position of the three genera within the Actinocerida was generally unstable, at least some analyses recovered a closer relationship of *Pseudowutinoceras* to *Sibumasuoceras* and *Langgunites* (PP = 0.18; Fig. 2, Additional file 1: Figs. S6-S9, S11-S14), thus supporting a recent suggestion to synonymize the Pseudowutinoceratidae with the Greenlandoceratidae [65], potentially including the Ruedemannoceratidae and the Gouldoceratidae within this group as well. In conclusion, many taxa previously assigned to the Discosorida are in fact unrelated. Further research is needed to clarify the relationship between oncocerids, discosorids, and apocrinoceratids.

The Ascocerida, which exhibit some of the most specialized and derived conch morphologies among Paleozoic cephalopods [78], were recovered as monophyletic in all analyses (PP = 0.97). Although one lineage, the Hebetoceratidae is relatively poorly known and arguments have been brought up that they may evolved independently from the Ascoceratidae [42], our analyses give strong support for the monophyly of the order, with the Hebetoceratidae (PP = 0.32) as the sister group to the Ascoceratidae (PP = 0.95). While the Ascocerida were suspected to be descendants of orthocerids such as *Clinoceras* [79], more recent research suggested the alternative hypothesis of an origin from the Uranoceratidae, which was in turn derived from an unspecified part of the Orthocerida instead of being part of the Barrandeocerida [78]. Yet another hypothesis suggested that the Ascocerida were derived from the Tarphycerida, but this was mainly a result of accepting the aforementioned relationship to the Uranoceratidae while seeking the ancestors of the latter within the Barrandeocerida, which was merged with the Tarphycerida [39]. Our results resolve this apparent conflict in recovering a consistent sister group relationship between Ascocerida and a clade containing the Uranoceratidae and the Apsidoceratidae, while at the same time retaining *Clinoceras* as an early member of this monophyletic clade (PP = 0.28). *Clinoceras* was mostly positioned early on the Ascocerida branch, shortly after its divergence from the Uranoceratidae and Apsidoceratidae; however, other datasets recovered it on the early uranoceratid branch. The origin of this Uranoceratidae-Apsidoceratidae-Ascocerida clade is more surprising, because it was recovered in the majority of the analyses as descendant or sister group of the Oncocerida (Fig. 2, Additional file 1: Figs. S6, S7, S9, S11-S15, S17). However, the picture is not quite clear, as some analyses favored a closer relationship to the Orthoceratoidea (Additional file 1: Figs. S8, S10, S16), with *Hedstroemoceras* as possible link to *Clinoceras*. Because the analyses recovering an orthoceratoid origin of the Ascocerida involved either removing a significant number of characters or species, or speculative coding of characters, we here favor an oncocerid origin of the group, although we note that both *Clinoceras* and *Hedstroemoceras*, the apparent key species for this relationship, need to be restudied to further test this hypothesis.

## Discussion

### Posterior probabilities and consensus trees

Low clade supports suggest high uncertainties in a clade. However, we repeatedly recovered similar—though not identical—clades in analyses of different subsets of data, although we would expect to frequently sample alternative topologies when uncertainty is this high. Additionally, quartet similarities suggested high congruence between the trees (mean quartet similarity of posterior trees to MCC tree of main analysis = 0.85; Additional file 1: Table S4). When using pruned MCC trees, the posterior probabilities of clades were much higher. Therefore, the species retained in the pruned MCC trees had much more stable positions relative to each other than suggested by the full MCC trees. For example, the Multiceratoidea had a posterior probability of 0.79, suggesting that the multiceratoids retained in the pruned MCC tree are more closely related to each other than to any other taxa in the pruned MCC tree, even though the posterior probabilities in the full MCC trees do not seem to lend strong support to this relationship. Because the species retained in the pruned MCC tree are mostly members of well-established taxonomic groups that already received moderate to strong support in the full MCC trees, the uncertainty in tree topology is not as high as it may appear at first. The question is, what causes the low clade posterior probabilities? A part of the reduced posterior probabilities likely results from the way the software BEAST summarizes trees that contain sampled ancestors (see [14] for an explanation), but this effect is probably minor, as the number of sampled ancestors was generally very low in our analyses.

Wildcard taxa can have a detrimental effect on the estimation of tree topology because they obscure otherwise stable phylogenetic relationships [23–26]. Importantly, they not only lower posterior probabilities of immediate neighboring nodes, but on all associated nodes, even if other relationships remain highly stable. This problem is primarily not linked to the inference—if conflicting evidence exists, then alternative topologies should be sampled with corresponding frequencies—but can mainly be attributed to the way posterior tree space is summarized in summary trees. Wildcards result in low posterior probabilities even when the number of wildcards is relatively small. As shown by the leaf stabilities and node distances (Fig. 8), there is a continuous distribution between taxa that are highly unstable and those that shift by a few nodes. More unstable taxa are obviously more problematic for phylogenetic inference (at least for the relationships between higher taxa) because it creates a conflict between two or more distant branches in the summary tree, consequently decreasing posterior probability for all involved clades towards the root. Fortunately, only a minority of the taxa included here appear to attach frequently to very distant branches. While several taxa here showed wildcard behavior, this was mostly restricted to local clades. Interestingly, taxa that were identified as the most unstable using leaf stability indices were also the most susceptible to modifications of the dataset, while taxa with higher leaf stabilities tended to be recovered in similar positions across different datasets. Our example illustrates that posterior probabilities in consensus trees may not fully capture the information contained in a set of trees, as even a small subset of locally unstable taxa can obscure otherwise stable patterns (see also [24, 26, 80, 81]). Another issue is that taxa and nodes that lie close to the center of the tree (which is in our case near the root that contains more balanced splits, i.e., edges that split the taxa roughly in half) contribute disproportionately to lower posterior probabilities, because the number of species that need to be contained in the same clade is higher compared to a distal node that contains only two sister species.

This problem is challenging to overcome. Excluding potential wildcard taxa in the CtEl and CtIc analyses did not lead to notably increased posterior probabilities. However, the wildcards may inform the analysis despite being difficult to place themselves. We demonstrate that it is possible to use a subset of tips of interest to reconstruct a pruned MCC tree based on the tree posterior obtained from the complete dataset. Although pruned MCC trees rely on the somewhat arbitrary selection of taxa, it ensures that only phylogenetic relationships of interest are considered. Our goal was to resolve relationships between major groups (orders) of early cephalopods, and thus, representatives of specific lineages of each order were selected for the pruned MCC trees according to a number of criteria (see “Methods”). This approach results in consensus trees that contain clade support values that better reflect the phylogenetic signal of the involved groups. Nevertheless, we stress that pruned MCC trees are not meant to replace full MCC trees and careful investigations on the distribution of trees in the posterior sample are necessary. We still recommend carrying out phylogenetic inferences on an extensive set of species. As shown by our CtEl and CtRd analyses, excluding species a priori leads to decreased posterior probabilities and slightly different, potentially less accurate topologies being recovered, even in the pruned MCC trees. Furthermore, using the same taxa as in the pruned tree of the CtCo analysis directly in another run resulted in a very different tree topology with lower posterior probabilities (Additional file 1: Fig. S24), which may indicate the importance of including all character and taxon information to minimize homoplasious transformations being reconstructed as homologous.

### Tree similarities

Much of the discussion on clade posterior probabilities also applies to the bipartition tree similarity metric used in this study. Although bipartition similarities seem to suggest that there is little in common between the topologies of the MCC trees, most of the clades can be recognized regardless of the analyzed dataset. Quartet similarities reveal much higher similarities between the trees, confirming that the underlying structures of the trees have much in common despite low bipartition similarities (note that two random trees have an expected bipartition similarity of 0.0 and a quartet similarity of $$\raisebox{1ex}{$1$}\!\left/ \!\raisebox{-1ex}{$3$}\right.$$ [22]). The same is true if the MCC trees are compared to the entire posterior sample. The distributions of the quartet similarities are congruent, regardless of whether the posterior tree sample is compared to the MCC tree from the same analysis or the MCC tree of the main dataset. This becomes even more apparent when the posterior is compared to the pruned MCC trees, where in most cases, a large part of the distribution is concentrated at high values and a relatively long tail towards lower values. Our results confirm that the use of bipartition tree metrics, such as the still widely used Robinson-Foulds-Distance, conveys an incomplete image of actual distributions of tree space and should always be used with caution [22, 50], although some more sophisticated bipartition metrics such as information-theoretic generalized Robinson-Foulds metrics circumvent some of these problems [50]. Moreover, since clade posterior probabilities are ultimately based on bipartitions, they may convey a similarly incomplete picture of clade support, particularly when the clades in question contain a large number of taxa.

### Implications on Cephalopod evolution, phylogeny, and systematics

The phylogenetic relationships suggested here are an important step forward, because contrary to previous studies, they represent the results of a quantitative approach to examine the early radiation of cephalopods using a large quantity of morphological data, while at the same time incorporating the stratigraphic distribution of taxa. Our results are in many cases consistent with previous ideas on early cephalopod evolution, but also refine these hypotheses, resolve apparent controversies, and reveal some surprising relationships. Although uncertainty is high, particularly concerning the affinities of early-diverging taxa near the root, most relationships are robust against alterations in character and taxa selection. We thus provide a framework for a modern phylogenetic classification of early Paleozoic cephalopods, consisting of the subclasses Endoceratoidea, Multiceratoidea, and Orthoceratoidea. At least two other subclasses are thought to originate from the Orthoceratoidea, the Coleoidea, and the Ammonoidea [40]. In our framework, the Nautiloidea consists only of the order Nautilida, possibly originating from the Orthoceratoidea or the Multiceratoidea [32]. Identifying the origin of the Nautilida will be important because it has major implications on the extent of the cephalopod crown group. If the cephalopod crown group originated around the Silurian-Devonian boundary as suggested by molecular studies [30–34], the extant Nautilida lineage can only have originated from within the Orthocerida, since the origin of the extant Coleoidea from that group is currently undoubted [82]. In this case, most or all taxa studied here would actually belong to the stem group. However, transitional fossils between orthocerids and nautilids are currently lacking, and paleontological studies therefore favor an origin from members of the Multiceratoidea (Tarphycerida or Oncocerida) [39]. If this is the case, then the crown group would include a much larger number of taxa and the stem group would only consist of plectronocerids, yanhecerids, and some Cambrian ellesmerocerids. However, in order to solve this issue, the ancestral lineage(s) of Silurian and Devonian nautilids need to be traced among Ordovician taxa. Molecular divergence dates of cephalopods typically have large confidence intervals, and the influence of fossil calibrations (which heavily depend on the phylogenetic assignment of fossil lineages) has not been extensively tested [30–34].

**Fig. 9:**
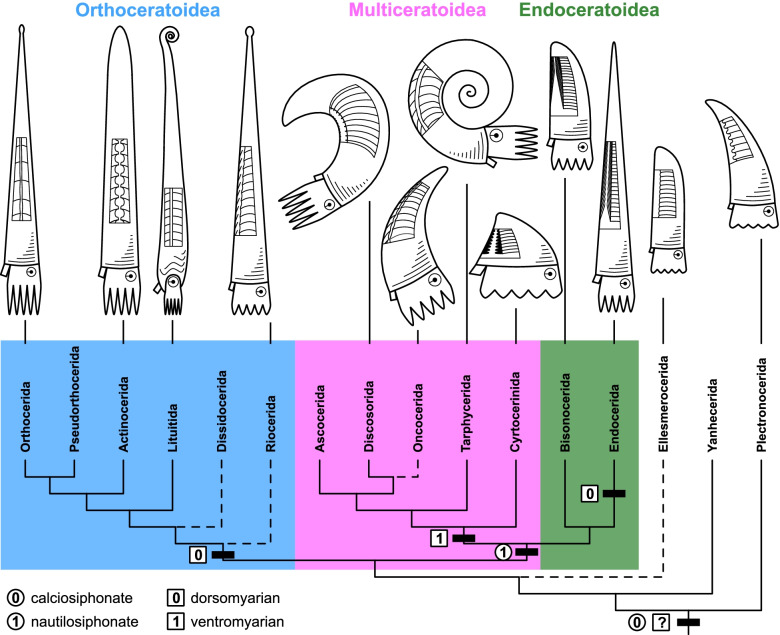
Simplified cladogram of early Paleozoic cephalopods. Colored names at the top and boxes represent subclasses, and other names are on order level. Dashed lines represent paraphyletic groups. The evolution of connecting ring type (circles) and muscle attachments (squares) is plotted on the tree. Note that this figure represents a simplification and some exceptions from these patterns occur. Drawings of soft parts are speculative and shell proportions not to scale. Orientations do not necessarily represent assumed positions during life

Previous studies have placed much emphasis on a rather small set of characters. In particular, the connecting ring ultrastructure and muscle attachments have been attributed with high relevance for the systematics of early cephalopods [39, 45]. Our results support the importance of these groups of characters but reveal a slightly different evolutionary pathway (Fig. 9). In the case of the connecting ring ultrastructure, conclusions were made on entire orders despite only a small number of species being investigated [45]. This is especially problematic in the case of ellesmerocerids, which represent a paraphyletic or even polyphyletic group. Ellesmerocerids were described as nautilosiphonate, despite the only species investigated being Middle Ordovician representatives [46], which were recovered by our analyses as multiceratoids. It is also important to highlight that ventromyarian, dorsomyarian and oncomyarian muscle attachments are not homologous, as the former two terms describe the relationship between the size of the dorsal and ventral retractors, while oncomyarian refers to an increase in retractor muscle number. One of the few ellesmerocerids with known “oncomyarian” muscle attachments, *Paradakeoceras minor*, is actually dorsomyarian, because the area of the annular elevation is larger above the dorsum than above the venter [83]. Furthermore, longitudinal striae on internal molds of phragmocones have been identified as oncomyarian tracks, but similar markings can also be produced by drag bands [84]. Future studies will therefore need to clarify whether drag bands and possible longitudinal tracks of oncomyarian muscle attachments can be unequivocally distinguished from each other. Muscle attachments are still poorly known in the earliest cephalopods. In contrast, the most parsimonious solution would be that the ancestral state is dorsomyarian, as already assumed earlier ([42], but see [39, 46, 83]). Better preserved material will be required to resolve this question.

The proposed classification scheme discussed in this article is summarized in Table 2. Some of the groups recognized here are explicitly paraphyletic (Ellesmerocerida, Oncocerida, Riocerida, and Dissidocerida; Fig. 9), even though this is generally to be avoided in modern phylogenetic classifications. As mentioned above, this extends to the Orthocerida, the presumable ancestors of the Ammonoidea and Coleoidea, and potentially also the Tarphycerida—if they should turn out to be ancestral to the Nautilida in future studies. The reason for retaining these taxonomic units despite their paraphyly is that they form coherent morphological groups and our results do not alter their original phylogenetic concepts. In addition, many species of the relevant groups have uncertain affinities and cannot be assigned to any of the descendant lineages with confidence. Assigning Paleozoic cephalopods into exclusively monophyletic groups would require (1) high confidence in the phylogeny of the entire group at a low taxonomic level and (2) a major reclassification that involves introducing numerous new names and taxonomic ranks. Because several affinities remain uncertain even after this study and we included only a fraction of the known diversity, the latter step is not attempted here, as future studies may overturn this classification, creating further instability in the already complex systematics of early cephalopods. These challenges are not unique to cephalopods, but more generally to intermediate taxa that occupy evolutionary positions close to the origin of a clade within a paraphyletic parent grouping.

**Table 2:** Proposed classification scheme of the Cephalopoda

**Class Cephalopoda Cuvier, 1797**	
Order Plectronocerida Flower, 1964	
Order Yanhecerida Chen et al., 1979	
Order Ellesmerocerida (*) Flower in Flower & Kummel, 1950	
**Subclass Orthoceratoidea Teichert, 1967**	
Order Riocerida (*) King & Evans, 2019	
Order Dissidocerida (*) Zhuravleva, 1964	
Family Bajkaloceratidae Balashov, 1962	
Family Intejoceratidae Balashov, 1960	
Family Geisonoceratidae Zhuravleva, 1959	
Order Lituitida Starobogatov, 1983	
Order Orthocerida Kuhn, 1940	
Order Pseudorthocerida Flower & Caster, 1935	
Order Actinocerida Teichert, 1933	
Family Greenlandoceratidae Shimizu & Obata, 1935	
Family Ruedemannoceratidae Flower, 1940	
Family Gouldoceratidae Stait, 1984	
**Subclass Endoceratoidea Teichert, 1933**	
Order Endocerida Teichert, 1933	
Order Bisonocerida Evans & King, 2012	
Family Padunoceratidae Balashov, 1960	
**Subclass Multiceratoidea Mutvei, 2013**	
Family Bassleroceratidae (*) Ulrich, Foerste, Miller & Unklesbay, 1944	
Family Cyclostomiceratidae Foerste, 1925	
Family Apocrinoceratidae Flower in Flower & Teichert, 1957	
Family Clinoceratidae Flower, 1946	
Family Uranoceratidae Hyatt in Zittel, 1900	
Family Apsidoceratidae Hyatt, 1884	
Order Cyrtocerinida Flower, 1964	
Order Tarphycerida Flower in Flower & Kummel, 1950	
Order Oncocerida (*) Flower in Flower & Kummel, 1950	
Order Discosorida Flower in Flower & Kummel, 1950	
Order Ascocerida Kuhn, 1949	
**[Subclass Nautiloidea Agassiz, 1847]**	
**[Subclass Ammonoidea Zittel, 1884]**	
**[Subclass Coleoidea Bather, 1888]**	

Under this scheme, several orders have no corresponding subclass, and several families are without order. These relationships will have to be solidified in the future and potentially assigned to other groups or new names will need to be established. Note that only families that differ in their systematic assignment from previous publications are listed here. The detailed content of the orders may differ from previously published studies, please consult the text and Additional file 1: Fig. S6-S17 for details. Groups marked with (*) are likely paraphyletic but are here retained for convenience. Subclasses in square brackets were not included in the analyses but are here listed for completeness. Class and subclass level are highlighted in bold

The phylogeny presented here may be further refined by future studies testing additional models or including more data. One promising avenue would be to incorporate models that can handle continuous characters directly [85], which have recently been developed for BEAST2 [86].

### Implications for phylogenetic analyses of fossil datasets

Our results suggest that small deviations in our dataset do not have a major impact on tree topology. Although we do not know, which of our analyses recovered the tree that is closest to the “true” tree, we are able to make some recommendations for the selection of characters and taxa in this or similar empirical datasets. To establish how widely these conclusions apply to other datasets, they should be further tested, ideally using simulation studies. Including a small number of controversial characters does not change the main topological patterns recovered by the analysis, but instead may increase uncertainty. When in doubt, it is probably safer to exclude them. If strongly conflicting topologies are recovered, it may be a good idea to reassess the morphological merits of these controversial characters first. It also suggests that there is some tolerance for incorrectly scored characters, which will not affect tree topology to a large degree if it affects only a small proportion of characters and taxa. It is conceivable that incorrectly scored or coded characters may have a larger impact in smaller datasets (see also [87]). Excluding taxa with incomplete character sampling can increase posterior probabilities of clades, but this does not guarantee that we will recover more correct clades [25]. This is exemplified by the Bisonocerida, which are mostly known from fragmentary material. This group was recovered as a monophyletic group with high posterior probabilities in the analysis of the main dataset, but not when incomplete species were excluded, potentially because the taxa were separated by large stratigraphic gaps. On the other hand, some incompletely known taxa were more prone to jump around the tree and thus increased topological uncertainty. Remarkably, excluding pseudoduplicates, which were selected to fill a few of the taxonomically underrepresented stratigraphic intervals in the character matrix, led to alternative topologies being recovered in the MCC tree. While pseudoduplicates had only a minor impact on parameter estimates (apart from the number of sampled ancestors, which can be explained by a tendency for pseudoduplicates to be recovered as sampled ancestors due to their identical character scorings), their removal led to a marked increase in topological uncertainty, as demonstrated by posterior probabilities (Figs. 5 and 6) and tree similarities (Fig. 7, Additional file 1: Table S4) compared to some analyses that excluded a larger amount of data. This highlights the importance of even sampling through time, as the pseudoduplicate species provide some important constraints on some diversification events. However, the number of pseudoduplicates was minimal in comparison to the total number of sampled species and total diversity. If these proportions were larger and unevenly distributed among lineages, it is conceivable that this approach may introduce biases in divergence time estimates and possibly also topology. Nevertheless, if used sparingly, it may be an efficient tool to approximate certain lineages while reducing the workload of character scoring. No other sampling approach led to increased clade posterior probabilities, thus supporting the idea that including as much data as possible improves node support, which should also lead to tighter confidence intervals for parameter estimates, even if some species contain high amounts of missing data.

We furthermore recommend the inclusion of characters containing high amounts of missing data or gaps, as excluding them entirely led to increased uncertainties in the form of wider ranges of tree similarities and decreased posterior probabilities. Depending on the research question, it may be advisable to produce, besides the full MCC tree, a number of pruned MCC trees that contain only a smaller fraction of the taxa, as this will more reliably show large-scale phylogenetic relationships between groups of interest. However, the selection of these taxa must be made carefully. It should be recognized that pruned taxa are part of the same diversification process, and they need to be considered when postulating monophyly or sister group relationships. This approach can also be combined with other approaches that show the position of omitted taxa in consensus trees [28, 88].

## Conclusions

Early cephalopod evolution is characterized by a late Cambrian or Early Ordovician split into three major clades: Endoceratoidea, Multiceratoidea, and Orthoceratoidea. This is supported by a comprehensive phylogenetic analysis that clarifies relationships between early members of the Cephalopoda. While some uncertainties persist, our study represents a significant step forward from previous classification attempts [38, 39, 41, 42, 45, 46], because it incorporates a large quantity of morphological data instead of using only isolated characters, which were subjectively attributed to be of high phylogenetic relevance. Our study can form the basis for a range of studies investigating evolutionary patterns of cephalopods. It also has important implications for the identification of the cephalopod crown group, depending on whether the *Nautilus* lineage originated from the Orthoceratoidea as suggested by molecular studies [32] or from the Multiceratoidea as inferred from morphological data [39].

Furthermore, we have shown that uncertainties commonly reflected by summary trees inferred from morphological data can be caused by variable branching patterns between closely related taxa, analogous to the negative impact on clade support of wildcard taxa [23, 26, 27, 29]. At the same time, we have provided arguments to include as many characters and taxa as possible in phylogenetic studies using the FBD model. To avoid uncertain topological placement of ancestral taxa obscuring the strength of clade supports, we propose the additional implementation of pruned MCC trees, which only reflect relationships between taxa that are regarded as most relevant to the study. This approach provides posterior clade probabilities that better reflect the phylogenetic signal between the depicted clades. It is important to keep in mind that conclusions about sister group relationships or monophyly need to consider taxa that were not included in the pruned MCC tree, but these trees can provide a better understanding of the evolutionary patterns of major lineages. Nevertheless, future studies should investigate possible alternatives to posterior clade probabilities that more accurately reflect uncertainties in large-scale phylogenetic relationships.

Lastly, our results demonstrate that Bayesian inference, in particular the FBD model, is well suited for the analysis of morphological data. We encourage researchers to use these tools, in particular for invertebrate paleontology, despite the past reluctance to adopt phylogenetic methods [89].

## Methods

### Morphological character matrix

A total of 141 morphological characters were newly defined and compiled for all taxa by a thorough investigation of the material and a review of the literature. We redefined many characters to keep them as objective as possible, while at the same time not neglecting the careful work that has been done since the early eighteenth century. The dataset comprises 14 characters on general conch shape, 8 characters on shell ornamentation, 15 characters on the shape of the body chamber, 11 characters on muscle attachment scars, 12 characters on the septa, 5 characters on cameral deposits, 29 characters on the siphuncle, 30 characters on endosiphuncular deposits, 12 characters on the embryonic shell, and 5 characters on the ontogeny. We made extensive use of contingent (or reductive) coding, which means that characters are hierarchically dependent on each other. Despite potential problems, this is currently the best available option for this study [90–92]. Although there has been recent progress in the development of methods dealing with inapplicable characters, most of these are not yet available for Bayesian inference, as they require different algorithms or coding strategies [92–94]. Another recently developed alternative method using structured Markov models is computationally intensive for large hierarchies and needs further testing in empirical datasets [95]. To facilitate future research and adaptations of the dataset, we organized the character definitions in a hierarchical way, for example:

1. Character A (description)
  1.1 Character B (description); inapplicable if character 1. is scored as state 0

In addition to making character state dependencies immediately obvious, this approach also allows for an intuitive representation of complex hierarchical relationships between characters on multiple levels.

Because of the comparatively low number of discrete characters, nautiloid taxonomy is based to a significant degree on measurements and shell proportions. The standard procedure for such continuous characters to be included in phylogenetic analyses has traditionally been to discretize them. Although studies have shown that including continuous characters can improve phylogenetic results, there are still challenges as to how they should be best included [85, 96, 97]. While we primarily collected continuous data, we discretized them because models of continuous character evolution were not yet implemented in the software BEAST2, though they will soon be available [86]. Nevertheless, in order to make the discretization process as transparent as possible, all continuous characters and their measurements are listed in Additional file 3: Data S2, and the distributions of continuous characters with break points are shown in Additional file 1: Figs. S3-S5. Since the distributions were mostly continuous with little natural break points, we based our discretization strategy on the following criteria:

1. Visual inspection of the distributions of each continuous character with identification of peaks.
2. Knowledge of the taxonomic distributions of these continuous characters, particularly outside of the sampled taxa.
3. Biologic or geometric relevance of the characters (e.g., structure A smaller, larger or similar to structure B).

Further detailed definitions and discussions for all characters can be found in Additional file 1: Text S1.

We tested different sets of characters to evaluate their influence on tree topology (Table 1, Additional file 1: Text S2). The first of these sets consisted of the inclusion of controversial characters. These characters are considered to be controversial because differences in these characters have been attributed partly or fully to taphonomy, which would invalidate their use for phylogenetic analysis. For example, while the differentiation of the connecting ring has been used in the past to distinguish between higher taxonomic units, the extent of diagenetic alteration is unclear and should be interpreted with caution [39]. Furthermore, we tested the removal of characters with high amounts of missing data or gaps, and the removal of autapomorphic characters. The latter was done to test whether removing autapomorphies influenced tree topology, as studies have shown its importance for tip-dating, though many datasets do not include them [98]. All character sets used the complete set of species. The dataset that excluded controversial characters was taken as the main dataset, and consequently, all character sets except for the complete set also excluded these characters. In addition, rather than testing every possible combination of character and taxon sets, we tested every taxon set with the character set excluding controversial characters. We tested three slightly modified datasets, in which we scored certain characters more speculatively. The reasoning behind the speculatively scored datasets was that these characters have been given high relevance for the phylogenetic relationships between nautiloid cephalopods [39, 45]. The type of the connecting ring has been investigated only in a limited number of species [45]. Similarly, muscle attachment scars are generally rarely preserved and thus also known in relatively few taxa [39]. Nevertheless, where known, these character states appear to be consistent with the higher-level classification [39, 45]. To account for this, we scored those characters in the speculative datasets in a way that assumes that species of the same order have identical states for those characters. We only applied those speculative scorings where they could be applied with some certainty. For example, species with unclear affinities and many early species of the Ellesmerocerida were scored as missing data. However, we emphasize that this approach is not recommended as a practice and should generally be avoided. Our specific purpose here is to test the phylogenetic signal of some characters that have historically been deemed more important to cephalopod systematics (i.e., having strong phylogenetic signal) in comparison with our standard dataset. All character matrices are available in the additional files.

### Species selection

The dataset includes in total 173 species of cephalopods from the Cambrian and Ordovician, listed in Additional file 2: Data S1. The taxa were selected to represent as much of the Cambro-Ordovician cephalopod diversity as possible. Thus, at least one representative species of most families was included, covering most of the duration of that taxon. Particular attention was paid to early representatives of their respective groups, as these are potentially highly informative in the analyses because they are expected to be closer to the ancestral morphotype of a particular clade. Other criteria were availability, accessibility, and completeness of the material. Generally, we included no more than one species of the same genus, but to increase the information content of the data set, we included character data from species that can be assigned to the same genus with confidence. Thus, although we used species as operational taxonomic units, most taxa are representative of an entire genus, although for some species, the genus attribution may be questionable (see Additional file 2: Data S1), and we thus refer to species. In addition, four species of especially long-ranging genera were duplicated together with their characters to represent the stratigraphic range of a certain morphotype more correctly. These additional species are here termed “pseudoduplicates”: *Bactroceras mourguesi*, *Bassleroceras perseus*, *Ectenolites primus*, and *Sinoceras fenxiangense.* Except for *B. perseus*, all pseudoduplicates are stratigraphically older than their template species. The gaps separating the pseudoduplicates are between 10 and 14 my, except for *E. primus*, which occurs in the time interval directly prior to *Ectenolites clelandi*. The latter is still included because the genus is thought to be one of the few genera to survive the Cambrian–Ordovician boundary [51, 99]. Thus, the criteria for the inclusion of pseudoduplicates were either the long range of a genus and corresponding gaps between template and pseudoduplicate species, or the significance of a genus surviving a particular time interval. Furthermore, pseudoduplicates were included where they are potentially evolutionary relevant, i.e., taxa that have previously been suggested to be ancestral to one or more descendant lineages. For this reason, we did not include any Late Ordovician pseudoduplicates, because they are unlikely to inform major evolutionary relationships. The age of the pseudoduplicates ranges from the late Cambrian (*E. primus*) through the Early Ordovician (*B. mourguesi*) to the Middle Ordovician (*B. perseus*, *S. fenxiangense*). Although pseudoduplicates may introduce bias in sampling proportion, this effect is here considered minimal, since the overall sampling proportion is very small and the number of pseudoduplicates is also kept to a minimum.

As for the character sets, we used different sets of species to evaluate the effect of species selection (Table 1, Additional file 1: Text S3). In one analysis (CtDp), pseudoduplicates were removed to test whether they had any influence on tree topology. Furthermore, we excluded species that had large amounts of missing data in one analysis (CtIc), to test whether removing incomplete taxa increased support for clades. For a similar reason, we tested another taxon set (CtEl) excluding most Ellesmerocerida, Plectronocerida, and Yanhecerida. These late Cambrian and Early Ordovician species are generally similar in morphology, which leads to additional “wildcard taxa” that may increase uncertainty, if they are prone to attach to any of the early branches. Thus, the branching order may change without a large detrimental effect on posterior probability. Only three ellesmerocerid species were included in the CtEl set, because they represent typical yet distinct morphotypes present in these groups and are known from comparatively complete material: *Bassleroceras champlainense* (exogastric longicone), *Ectenolites clelandi* (endogastric longicone), and *Clarkoceras newtonwinchelli* (endogastric brevicone). All ellesmerocerids, plectronocerids, and yanhecerids fall into one of these categories and furthermore share a ventral siphuncle with concave or tubular segments [42, 100], with the exception of the specialized plectronocerid siphuncle [52–54]. Further differences between them are often relatively subtle. Note that while the family Cyclostomiceratidae is usually also classified within the Ellesmerocerida, we did not exclude them for this analysis because they share some unique characteristics that set them apart from other ellesmerocerids [101]. Lastly, as the character/taxon ratio of our dataset is rather low compared to what it usually recommended [102–104], we tested another set where half the species were randomly removed (CtRd) to see the general influence of taxon sampling on resolution. Since the focus was on the reduced number of species (and consequently, increased character/taxon ratio) instead of the randomization, we did not perform additional iterations of taxon removal, which would be expected to result in somewhat different topologies and tree similarity values. The results from this run should therefore be interpreted with some caution. Nevertheless, it is consistent with all our character and species selection choices in that the criteria are explicit with using only a single threshold value (e.g., proportion of characters with missing data). For a more in-depth analysis investigating the effects of any of these criteria, multiple runs with different threshold values (or randomizations) would be necessary, but for the purpose of testing the sensitivity of the results to modifications of the dataset, we consider this approach reasonable.

We did not include any nectocaridids in the analyses, since their interpretation as cephalopods [105–108] has been previously refuted [32, 40, 53, 109–113], and is not accepted by the majority scientific consensus. Besides the arguments brought forward by earlier critiques, we want to add that if they were cephalopods, it would require a major ontogenetic re-organization of the body axis prior to the acquisition of a shell. The functional anterior side of squids is anatomically the ventral side, and the arms homologous to the molluscan foot [114]. By contrast, there is no evidence in *Nectocaris* for a similar developmental organization and we cannot imagine a plausible evolutionary pathway that would link basal mollusks to nectocaridids, involving the transformation of the foot into arms (which in embryonic cephalopods are situated ventrally—posterior with reference to the ancestral molluscan orientation—to the head region and move forward to enwrap the mouth [114]), an elongation and flattening of the mantle cavity and a tilt in the dorsoventral body axis. Research postulating a nectocaridid origin of cephalopods has focused on the homologization of nectocaridid and cephalopod characters [105, 107], but these are at odds with the known homologies between cephalopods and basal mollusks. Besides, even if nectocaridids were accepted as cephalopods, the soft-bodied nature of their fossils makes it impossible to include them in the analysis, since there is no overlap in characters with contemporary cephalopods, even if Late Ordovician nectocaridids with a diffuse “internal shell field” were included [108].

We also did not include the potential early Cambrian cephalopod recently reported from Newfoundland [113], because we consider its identification unconvincing and further material is needed for clarification. The specimens are only known from disconnected longitudinal and cross sections, the former of which shows septa and the latter a putative siphuncle. The only evidence linking these two specimen types together are manganese enrichments, none of which are known from any other cephalopod [113]. Because of this and several other peculiarities mentioned in the original description, future studies will have to confirm whether these specimens are conspecific before it can be accepted as a cephalopod. In any case, the oblique sections of the specimens prevent the scorings of many morphological characters defined here. Late Cambrian cephalopods also resemble each other much more closely than any resembles the potential early Cambrian cephalopod, and therefore, it is reasonable that the last common ancestor of the late Cambrian cephalopods occurred only shortly before *Plectronoceras*.

### Bayesian total-evidence dating

We used the software BEAST 2.6.3 for the Bayesian tip-dating analyses [115]. To examine the impact of different datasets and to maintain consistency, we applied the same model for all our analyses. As a tree prior, we used the fossilized birth-death (FBD) model for all analyses, which explicitly models the processes of diversification and fossil sampling, and allows sampled species to give rise to sampled descendant species (sampled ancestors) [8–10]. We follow the parametrization by Heath et al. [8], i.e., net diversification rate (*d* = *λ* − *μ*), turnover (*r* = *μ* / *λ*) and sampling proportion (*s* = *ψ* / (*μ* + *ψ*)). To model character evolution, we used the Mkv model with invariant site correction [116]. Characters were partitioned according to their number of states but with the corresponding exchangeability values increased to half the number of states to correct for artificial upweighting of multistate characters [20]. We accounted for heterogeneous rates with eight discrete gamma distributed rate categories, which have previously been suggested to be optimal for morphological data sets [117]. Species were assigned stratigraphic ages with reference to the Ordovician stage slices [118] according to the literature and the RNames database [119]. For Cambrian taxa, we subdivided their age into four separate time intervals, which correspond to the *Ptychaspis*-*Tsinania* (late Jiangshanian), *Quadraticephalus*, *Acaroceras*-*Sinoceremoceras*, and *Mictosaukia* zone (Stage 10) in North China and their equivalents [51]. Fossil ages were allowed to vary between the upper and lower limits of the corresponding stage slices and Cambrian zones to account for stratigraphic uncertainty [120]. Although these stratigraphical intervals have no absolute age limits, there are between two to four stage slices per stage with absolute ages known [118]. We therefore divided each stage into time-equivalent time bins according to the number of stage slices. For example, the Dapingian ranges between 470.0 mya and 467.3 mya and consists of three stage slices (Dp1-3). Accordingly, we assigned the boundary between Dp1 and Dp2 to an age of 469.1 mya, and the boundary between Dp2 and Dp3 to an age of 468.2 mya. Where stratigraphic uncertainty encompassed multiple stage slices, tips were allowed to vary accordingly. In various cases, species occurred in the same horizon at the same locality, and they can thus be assumed to be contemporaneous. To ensure that the algorithm did not explore impossible states, where tips from the same fossil site are separated by several million years, we implemented fossil site operators, which synchronizes the age of tips from the same fossil site [121].

,We placed an informative exponential prior on the origin, with the mean equivalent to the upper limit of the time interval of the oldest doubtless known fossil cephalopod *Plectronoceras cambria* (mean 0.1, offset 489.5 mya). This prior is justified because cephalopods are exceedingly rare at that time, but become abundant in slightly younger strata, while still sharing very similar morphological characteristics and thus probably being very closely related [51, 99]. This makes it conceivable that even if cephalopods originated earlier, they did not diversify to a significant degree prior to the Cambrian Stage 10. Nevertheless, the exponential prior allows for older dates to be sampled and should infer an older origin time if there is strong evidence for this in the data. We placed further exponential priors on diversification rate (mean = 1.0) and sampling proportion (mean = 0.1, limited between 0.0 and 1.0). The prior on turnover rate was set to a uniform distribution between 0.0 and 1.0. We accounted for heterogeneous clock rates with a relaxed log-normal clock with an exponential prior (mean = 1.0) on mean clock rate and a gamma distribution on the standard deviation.

Each analysis was run for three independent runs of 200,000,000 generations, sampling every 10,000 generations with 10% of the samples discarded as burn-in. The analyses on datasets with lower numbers of species (CtIc, CtEl, and CtRd) were quicker to converge and we thus only used two independent runs. The log and tree files of separate runs were checked for convergence in Tracer [122] and combined in LogCombiner [115]. The combined tree files were then used to retrieve annotated MCC trees in TreeAnnotator [115]. Because high uncertainties near the root of the tree sometimes resulted in negative branch lengths, we chose the option “keep target heights” in TreeAnnotator, which takes node heights directly from the MCC tree. Note that this approach results in some cases where the node height in the MCC tree is outside highest posterior density intervals.

We produced another set of MCC trees where we retained only a small number of representative species from each group to provide clade support values that better reflect the phylogenetic signal of those groups. The species were selected to represent distinct lineages and stratigraphic ages of the respective groups, by inspecting the MCC tree obtained from the analyses and choosing sets of species with a most recent common ancestor as close as possible to the base of the clade under investigation. Thus, we ensured that the phylogenetic and stratigraphic range of the retained species of a particular group was as large as possible. Further criteria for the selection of species were completeness and topological stability. We pruned all other species from the posterior set of trees before producing these MCC trees in TreeAnnotator. Where a species was not included in a particular dataset, they were replaced by another species from the same group. We refer to these trees as “pruned MCC trees,” in contrast to full MCC trees, which contained the complete set of species. Note that no new analyses were conducted for the pruned MCC trees, and they are based on the same tree posterior as the full MCC trees.

To compare the impact of a posteriori and a priori pruning of taxa, we performed another small analysis, which was essentially the same as CtCo, but with the set of taxa retained in the pruned MCC tree. Because the much smaller number of taxa took a lot less time to converge, this analysis was only run once for 100,000,000 generations.

### Tree distances

To explore the impact of different datasets, we calculated the tree distances of the MCC trees recovered from the Bayesian analyses using quartet distances [22], which overcome several disadvantages of the commonly used bipartition metric Robinson-Foulds distance [22, 123]. Because the latter is still widely used, we also used bipartition distance as a comparison. We use the term similarity here instead of distance because a value of 1.0 represents two trees that are resolved exactly the same, while 0.0 represents complete dissimilarity, thus making comparisons more intuitive, but note that two random trees are expected to result in a bipartition similarity of 0.0 and a quartet similarity of $$\raisebox{1ex}{$1$}\!\left/ \!\raisebox{-1ex}{$3$}\right.$$ [22]. Both metrics were normalized, meaning that the number of partitions that were resolved was divided by the maximum number of partitions, thus avoiding differences that are due to having variable numbers of taxa. We also calculated rescaled quart et similarities, which have an expected value of 0.0 for two random trees. All MCC trees were treated as fully resolved point estimates (with sampled ancestors treated as zero-length branches), and therefore, quartet similarity corresponds to the number of quartets recovered in both trees divided by the total number of possible quartets. Since not all trees contained the same number of tips, only quartets of taxa that were shared between trees were considered. In addition to comparing the MCC trees betw een each analysis, we compared the MCC tree to the corresponding posterior tree sample. The distribution of the similarity metrics provides a measure of the overall similarity of the MCC tree to the trees contained in the full posterior. We furthermore compared each posterior tree sample to the pruned MCC tree from the same analysis and to the full and the pruned MCC trees obtained from the analysis excluding controversial characters (CtCo). Tree comparisons were done in R [124] using the tqDist algorithm provided by the Quartet package [22, 125, 126].

### Leaf stability

We calculated the leaf stability index (lsDif) [24] for each taxon in the CtCo analysis using RogueNaRok [26] to see if any taxa disproportionally affect tree topology. However, leaf stability indices alone are difficult to interpret and provide little information on the extent of the instability, i.e., how far the taxa move between trees. For this purpose, we calculated node distances between each taxon and its three closest neighboring tips on the full MCC tree of the CtCo analysis using the R package adephylo [127]. These distances were then compared to the distances between the same taxa in the entire posterior tree sample. Although it would be possible to calculate the distances between all possible taxon pairs, this is not anticipated to yield useful insights. Long distances may convey different phylogenetic meanings, e.g., if a tree consists of the three clades A, B, and C, similarly long distances may occur between taxa in A and B as between taxa in A and C. In contrast, short distances always indicate that the two taxa are situated in the same region of the tree. Nevertheless, this does not provide information on how the branches are resolved, except for the minimum distance of one node, which always indicates a monophyletic sister group relationship between two taxa, or that one taxon is a sampled ancestor of a second terminal taxon (as sampled ancestors are treated as zero-length branches). The number of measured node distances (= 3) was chosen as it creates quartet statements, in line with the quartet similarity metric, which is preferred over bipartition metrics [22].

## Supplementary Information

**Additional file 1: Text S1.** Character definitions, including hierarchical relationships and detailed discussions and justifications of each character. **Text S2.** Character sets, list of all characters that were excluded for each analysis. **Text S3.** Taxon sets, list of all taxa that were excluded for each analysis. **Text S4.** Supplementary references cited in Text S1. **Fig. S1.** Measurements. **Fig. S2.** Illustrations of characters. **Fig. S3.** Distribution of conch parameters (1/3). **Fig. S4.** Distribution of conch parameters (2/3). **Fig. S5.** Distribution of conch parameters (3/3). **Fig. S6.** Full MCC tree of the CtCo analysis. **Fig. S7.** Full MCC tree of the CoCo analysis. **Fig. S8.** Full MCC tree of the IcCo analysis. **Fig. S9.** Full MCC tree of the AmCo analysis. **Fig. S10.** Full MCC tree of the IaCo analysis. **Fig. S11.** Full MCC tree of the CrCo analysis. **Fig. S12.** Full MCC tree of the MaCo analysis. **Fig. S13.** Full MCC tree of the CMCo analysis. **Fig. S14.** Full MCC tree of the CtDp analysis. **Fig. S15.** Full MCC tree of the CtIc analysis. **Fig. S16.** Full MCC tree of the CtEl analysis. **Fig. S17.** Full MCC tree of the CtRd analysis. **Fig. S18.** Pruned MCC trees (1/6). **Fig. S19.** Pruned MCC trees (2/6). **Fig. S20.** Pruned MCC trees (3/6). **Fig. S21.** Pruned MCC trees (4/6). **Fig. S22.** Pruned MCC trees (5/6). **Fig. S23.** Pruned MCC trees (6/6). **Fig. S24.** MCC tree of an analysis containing the same taxa as the CtCo pruned MCC tree. **Table S1.** Details of measurements and their abbreviations. **Table S2.** Details of conch parameters and their abbreviations. **Table S3.** Equivalent nodes in full and pruned MCC trees. **Table S4.** Tree similarities.

**Additional file 2: Data S1.** Species list. This Excel file lists all species used in the analyses and includes a list of references. It furthermore lists corresponding stratigraphic horizons and their age ranges, and traditional views on the systematic assignment of each species (family and order level).

**Additional file 3: Data S2.** Measurements and conch parameters. Here, all measurements and the corresponding conch parameters for continuous characters are listed. Note that the measurements do not always represent raw values but are proportionally adjusted in cases where not all measurements could be taken from the same specimen at the same position. One specimen is always taken as reference specimen, this specimen is explicitly cited with figure and repository number. Repository numbers printed in bold represent that the specimens have not been tracked, i.e., the corresponding collections may have been moved or reorganized. Underlined repository numbers indicate that the measurements have been taken from the literature. In cases, where additional specimens were used for measurements, the values are underlined. Values that carry some uncertainty, e.g., due to poor preservation, are printed in bold. Institutional abbreviations are listed at the bottom end.

**Additional file 4: Data S3.** Leaf stability indices and node distances. This Excel file contains leaf stability indices and node distances obtained from the CtCo analysis. Abbreviations: END = Endoceratoidea; MUL = Multiceratoidea; ORTH = Orthoceratoidea; PEY = Plectronocerida/Ellesmerocerida/Yanhecerida; AgeMCC = age of the taxon in the CtCo full MCC tree; lsDif = leaf stability index (difference); lsEnt = leaf stability index (entropy); lsMax =leaf stability index (maximum); ct1mean = mean node distance to the closest tip in the MCC tree; ct2mean = mean node distance to the 2^nd^ closest tip in the MCC tree; ; ct3mean = mean node distance to the 3^rd^ closest tip in the MCC tree); ct1max = maximum node distance to the closest tip in the MCC tree; ct2max = maximum node distance to the 2^nd^ closest tip in the MCC tree; ; ct3max = maximum node distance to the 3^rd^ closest tip in the MCC tree.

**Additional file 5: Data S4.** Nexus files. This zip-file contains all character matrices used for the analyses, including alternative character scorings.

**Additional file 6: Data S5.** BEAST files. All xml-files used for the analyses and the corresponding summary trees are combined in a single zip-file.

**Additional file 7: Data S6.** R-script. This file contains the script file that was used to calculate node distances of the closest tips.

## Data Availability

All data generated or analyzed during this study are included in this published article [and its supplementary information files].

## Abbreviations

FBD: Fossilized birth-death
MCC: Maximum clade credibility
MRC: Majority-rule consensus
PP: Posterior probability
