# Supplementary material for: Early cephalopod evolution clarified through Bayesian phylogenetic inference
Source: BMC Biol. 2022 Apr 14;20:88. doi: 10.1186/s12915-022-01284-5 (PMC9008929; doi:10.1186/s12915-022-01284-5)
Supplement: Supplementary file 1 — Additional file 1: Text S1. Character definitions, including hierarchical relationships and detailed discussions and justifications of each character. Text S2. Character sets, list of all characters that were excluded for each analysis. Text S3. Taxon sets, list of all taxa that were excluded for each analysis. Text S4. Supplementary references cited in Text S1. Fig. S1. Measurements. Fig. S2. Illustrations of characters. Fig. S3. Distribution of conch parameters (1/3). Fig. S4. Distribution of conch parameters (2/3). Fig. S5. Distribution of conch parameters (3/3). Fig. S6. Full MCC tree of the CtCo analysis. Fig. S7. Full MCC tree of the CoCo analysis. Fig. S8. Full MCC tree of the IcCo analysis. Fig. S9. Full MCC tree of the AmCo analysis. Fig. S10. Full MCC tree of the IaCo analysis. Fig. S11. Full MCC tree of the CrCo analysis. Fig. S12. Full MCC tree of the MaCo analysis. Fig. S13. Full MCC tree of the CMCo analysis. Fig. S14. Full MCC tree of the CtDp analysis. Fig. S15. Full MCC tree of the CtIc analysis. Fig. S16. Full MCC tree of the CtEl analysis. Fig. S17. Full MCC tree of the CtRd analysis. Fig. S18. Pruned MCC trees (1/6). Fig. S19. Pruned MCC trees (2/6). Fig. S20. Pruned MCC trees (3/6). Fig. S21. Pruned MCC trees (4/6). Fig. S22. Pruned MCC trees (5/6). Fig. S23. Pruned MCC trees (6/6). Fig. S24. MCC tree of an analysis containing the same taxa as the CtCo pruned MCC tree. Table S1. Details of measurements and their abbreviations. Table S2. Details of conch parameters and their abbreviations. Table S3. Equivalent nodes in full and pruned MCC trees. Table S4. Tree similarities. [file 12915_2022_1284_MOESM1_ESM.pdf]

Early cephalopod evolution clarified through Bayesian phylogenetic inference

BMC Biology 2022

Supplementary information: Additional file 1

Alexander Pohle*, Björn Kröger, Rachel C. M. Warnock, Andy H. King, David H. Evans, Martina Aubrechtová, Marcela Cichowolski, Xiang Fang & Christian Klug

*Corresponding author. Email: alexander.pohle@pim.uzh.ch

This PDF file contains information on character definitions, character and taxon sets from the main article and additional figures and tables.

Text S1: Character definitions

This list contains definitions of all characters and their states, including references. The reasoning behind the respective coding scheme is explained for every character. It also includes discussions on alternative coding schemes. Measurements used for the delimitation of characters are shown in Fig. 1A (main article) and Additional file 1: Fig. S1 and corresponding abbreviations and calculations of character ratios are listed in Additional file 1: Table S1 and S2. Some character definitions are supplemented with further illustrations, shown in Additional file 1: Fig. S2.

The ventral side of the conch is identified as outlined by the Treatise on Invertebrate Palaeontology [1] and supplemented by Niko & Mapes [2]. Nevertheless, there are some cases where the identification of the ventral side remains challenging, especially among members of the Orthocerida. Since neutral coding with respect to the conch orientation is impossible because of the homology requirement, taxa are scored with the ventral side as indicated by previous studies. However, we are confident that the ventral side is correctly identified in the overwhelming majority of the taxa. Furthermore, many characters are independent of orientation and at least part of the phylogenetic signal should be preserved, even if the orientation was misidentified. Naturally, other researchers may adjust our character scorings in the future if more or better information on the orientation of certain taxa becomes available.

Ontogenetic changes are common for many characters; some of these are explicitly coded in characters 55–59. Otherwise, most characters are taken at a nearly adult position of the phragmocone, because adult modifications may significantly alter the shape of the terminal phragmocone [1,3]. These modifications are then treated as separate characters. In particular – but not exclusively, this applies to the Ascocerida, which have a significantly modified adult conch, which does not closely resemble any of the other taxa [1,4,5].

We make extensive use of contingent coding, i.e., coding characters as inapplicable depending on the character state of another character [6]. This is visualised in the character list with hierarchical levels. We also use this as a numbering scheme to refer to particular characters. For convenience, a continuous numbering is additionally supplied in brackets for each character.

Character dependencies are explicitly stated in such cases. Character models (CM) can be either unordered or ordered, which is explicitly stated for each character. Characters, that are essentially continuous were discretised and the distribution of the values are shown in Additional file 1: Fig. S3-S5.

1. Transverse ornamentation (#1)

0) smooth
1) lirae
2) ribs

Category: Shell ornamentation
CM: unordered

This character describes the external ornamentation of the shell. “Smooth” does not imply that growth lines are absent, but rather that they are only weakly visible and often discontinuous. “Lirae” are defined as distinctly raised growth lines, while “ribs” are raised even more and may be additionally broadened. Importantly, ribs do usually not leave traces on internal moulds, in contrast to annulations. The distinction between these categories is somewhat arbitrary since there is a continuum in the variation of this character. This

terminology essentially follows Kröger [7], but his “ridges” are here referred to as “ribs”. Additionally, since we did not observe any striae (grooves) in our dataset, they are not included here. Lastly, while there are minute differences in the cross section shape of these lirae or ribs [see 8], these are undocumented in the vast majority of taxa and are therefore not included here.

2. Frilled growth lines (#2)

0) absent
1) present

Category: Shell ornamentation
CM: unordered

Usually termed as “frilled collars”, they are defined as growth lines that are narrowly undulated such as in *Zitteloceras* Hyatt, 1884 [9]. Although they appear to be always raised (i.e., state 1 or 2 in character 1), there is no logical objection to the presence of smooth frills and therefore, they are not treated as subordinate to character 1.

3. Growth line direction (#3)

0) transverse
1) dorsapertural
2) ventrapertural

Category: Shell ornamentation
CM: unordered

The general direction of the growth lines is coded in relation to the central growth direction of the conch (see Additional file 1: Fig. S2A).

4. Growth lines course (#4)

0) straight
1) curved

Category: Shell ornamentation
CM: unordered

This character describes the general course of the growth lines seen from the lateral side. The lateral side is chosen here because straight growth lines may appear curved from the ventral side, if they are oblique in their direction, for example. Sinuses are explicitly not included here, because they are considered as modifications of the general course of the growth lines. Because there is a relationship between the direction of the growth lines and

dorsal and ventral sinuses, we do not distinguish between saddles and lobes in this character.

5. Hyponomic sinus (#5)

0) absent
1) present

Category: Body chamber
CM: unordered

The hyponomic sinus is positioned on the venter and defined as a lobe at the aperture [1]. This is also visible in more posteriorly positioned growth lines. Note that only distinct changes in the course of the growth lines are defined as sinus, even though in the literature, growth lines that slope uniformly in dorsapertural direction are sometimes also described as hyponomic sinus. Because the hyponomic sinus is the most widely distributed apertural modification, it is coded in more detail below.

5.1. Relative hyponomic sinus length (#6)

0) short (≤ 0.2)
1) deep (> 0.2)

Category: Body chamber
CM: ordered (discretised); effectively unordered

The sinus length is measured in adapical direction at the aperture or one particular adapertural growth line if the former is not preserved. The distance is measured between the most adapical point of the hyponomic sinus and the most adapertural point of the sinus according to the same definition of its margin as above. The relative sinus length (RHL) is then calculated as follows:

RHL = hl/hw

Where hl = hyponomic sinus length (in mm) and hw = hyponomic sinus width (in mm). The distribution of this parameter among taxa in our dataset is shown in Additional file 1: Fig. S3A.

Inapplicable if character 5 is scored as state 0.

5.2. Relative hyponomic sinus width (#7)

0) broad (< 0.9)
1) narrow (≥ 0.9)

Category: Body chamber
CM: ordered (discretised); effectively unordered

The sinus width is the distance between the left and right adapertural margin of the hyponomic sinus (if present, the most adapertural point of the adjacent apertural projections). If there is no clear definition of the margin of the hyponomic sinus, only the part of the growth line that is visible from the ventral side is considered. Thus, the maximum width of the hyponomic sinus encompasses the entire ventral view. This means that the maximum value that this character can attain is the same as the conch width. Accordingly, the relative hyponomic sinus width (RHW) is calculated as follows:

RHW = hw/cw

Where hw = hyponomic sinus width (in mm) and cw = conch width (in mm). The distribution of this parameter among taxa in our dataset is shown in Additional file 1: Fig. S3B.

Inapplicable if character 5 is scored as state 0.

5.3. Hyponomic sinus shape (#8)

0) round
1) sharp

Category: Body chamber
CM: unordered

This character describes the shape of the midventral plane of symmetry of the hyponomic sinus. At this point, the growth lines may produce a U- (state 0) or V-shape (state 1) [10].

Inapplicable if character 5 is scored as state 0.

6. Dorsal sinus (#9)

0) absent
1) present

Category: Body chamber
CM: unordered

In analogy with character 5, a sinus is only counted as present if there is a distinct change in the course of the growth lines.

7. Lateral sinuses (#10)

0) absent
1) present

Category: Body chamber
CM: unordered

As in characters 5 and 6, only distinct changes in the course of the growth lines are counted as sinuses.

8. Annulations (#11)

0) absent
1) present

Category: General conch shape
CM: unordered

In contrast to ribs (character 1), annulations involve the entire shell wall and thus leave traces on internal moulds. This is different from the Treatise [1], where no distinction between ribs and annulations was made, although they represent fundamentally different ways in shell construction [11]. Note that annulations may occur in combination with transverse ornamentation.

8.1. Annulation direction (#12)

0) transverse
1) dorsapertural
2) ventrapertural

Category: General conch shape
CM: unordered

As with growth lines (see character 3), the direction of the annulations is coded in relation to the central growth axis (see Additional file 1: Fig. S2A).

Inapplicable if character 8 is scored as state 0.

8.2. Annulation distance (#13)

0) short
1) medium
2) long

Category: General conch shape
CM: unordered

The distance between annulations is coded in relation to the distance between chambers. “Medium” represents the state where annulation distance is approximately equal to cameral length (character 65).

Inapplicable if character 8 is scored as state 0.

9. Longitudinal ornamentation (#14)

0) absent
1) present

Category: Shell ornamentation
CM: unordered

This character defines whether longitudinal ornamentation is present.

9.1. Longitudinal element strength (#15)

0) weak
1) distinct

Category: Shell ornamentation
CM: unordered

Distinct ornamentation is defined as being raised and clearly visible. Distinct longitudinal ornamentation is thus comparable in strength to lirae or ribs in transverse ornamentation (character 1).

Inapplicable if character 9 is scored as state 0.

9.2. Longitudinal element distance (#16)

0) narrow
1) wide

Category: Shell ornamentation
CM: unordered

The distance between longitudinal ornamentation elements is difficult to quantify, because it is unclear whether the number of elements is always constant throughout the ontogeny or whether additional elements are inserted during growth. Possibly, there is some variation in this mechanism among taxa. Here, we consider the distance as “wide” if the elements are separated by interspaces that are at least several times larger than the width of the elements themselves. Although there certainly exists some grey area in this regard, the distinction between these two types appears to work in most cases quite well.

Inapplicable if character 9 is scored as state 0.

9.3. Longitudinal element variation (#17)

0) uniform
1) variable

Category: Shell ornamentation
CM: unordered

Longitudinal ornamentation may be uniform, i.e., all ridges have the same strength and shape. However, there are also cases where the longitudinal ornamentation consists of interchanging minor and major elements or otherwise variable longitudinal ornamentation.

Inapplicable if character 9 is scored as state 0.

10. Transverse ventral outline (#18)

0) angular
1) narrow
2) round
3) flattened
4) concave

Category: General conch shape
CM: unordered

For characters 10–12, the cross section of the conch is divided into ventral, dorsal and lateral outline by horizontal and vertical lines through the middle of the cross section. The ventral outline consists of the lower half of this section. The boundary between different shapes is somewhat arbitrary but is drawn where the outline visibly diverges from a regular circle or ellipse (Additional file 1: Fig. S2B).

11. Transverse dorsal outline (#19)

0) angular
1) narrow
2) round
3) flattened
4) concave

Category: General conch shape
CM: unordered

Compare character 10 and Additional file 1: Fig. S2B for a detailed explanation. The dorsal outline consists of the upper half of the section. Note that there is a partial character correlation between involute conchs and a concave dorsal outline because of the imprint zone.

12. Transverse Lateral outline (#20)

0) angular
1) narrow
2) round
3) flattened
4) concave

Category: General conch shape
CM: unordered

Compare character 10 and Additional file 1: Fig. S2B for a detailed explanation. The lateral outline can be examined on the left or right half of the section. Concave lateral sides, i.e., lateral grooves are an autapomorphy of *Oneotoceras* Ulrich in Butts, 1926. Angular lateral sides are an autapomorphy of *Gonioceras* Hall, 1847.

13. Suture shape (#21)

0) straight
1) lobate

Category: Septa
CM: unordered

The sutures mark the position where septa and shell wall meet [1]. Treating each lobe of the suture line separately would result in putting increased weight on the suture line. With the coding applied here, a single step is required to change from a lobate to a straight suture or vice versa, a scenario we consider reasonable, since nautiloid sutures are

generally much simpler than ammonoid sutures. Note that a distinct ventral lobe or saddle in taxa with ventral siphuncles is here explicitly excluded from the suture, because it results from the position of the siphuncle rather than from an inflection of the septa [12–14]. The siphuncular suture is treated separately as character 67.2. There is also some co-variation with the conch cross section (character 64), since elliptical cross sections tend to produce lateral lobes [15,16], but it is challenging to completely disentangle these two characters.

13.1. Ventral lobe (#22)

0) absent
1) present

Category: Septa
CM: unordered

A ventral lobe consists of a single lobe on the venter in central position. Paired lobes are always treated as lateral lobes. If a ventral lobe is present, according lateral or ventrolateral saddles are invariably also present, thus the latter feature is not coded.

Inapplicable if character 13 is scored as state 0.

13.2. Dorsal lobe (#23)

0) absent
1) present

Category: Septa
CM: unordered

In analogy to character 13.1, lateral or dorsolateral saddles are present if a dorsal lobe is present.

Inapplicable if character 13 is scored as state 0.

13.3. Lateral lobes (#24)

0) absent
1) present

Category: Septa
CM: unordered

Lateral lobes are always combined with dorsal and ventral saddles, or if ventral and/or dorsal lobes are present, they are combined with dorsolateral/ventrolateral saddles. Lateral lobes are the most variable modification of the suture line in early Palaeozoic cephalopods and are thus characterized in more detail here.

Inapplicable if character 13 is scored as state 0.

13.3.1. Lateral lobe position (#25)

0) ventrolateral
1) midlateral
2) dorsolateral

Category: Septa
CM: unordered

This character describes the position of the lateral lobe in relation to the dorsum and venter. The position is taken at the point of maximum suture depth (see character 13.3.2). The boundaries between states are somewhat subjective, but only those that distinctly deviate from midlateral are scored as state 0 or 2.

Inapplicable if character 13.3 is scored as state 0.

13.3.2. Relative lateral lobe depth (#26)

0) shallow (≤ 0.15)
1) deep (> 0.15)

Category: Septa
CM: ordered (discretised); effectively unordered

When seen from the lateral side and the dorsal and ventral margins of the conch are connected by a straight line, the point of maximum suture depth is located where the distance between this line and the suture is the highest. This distance represents the absolute lateral lobe depth (ld). Accordingly, the relative lateral lobe depth (RLD) is calculated as follows:

$$RLD = ld/ch$$

Where ch = conch height. The outlier value shown in Additional file 1: Fig. S3C is represented by a single species in the dataset, namely *Gonioceras groenlandicum* Troedsson, 1926. The large value is in this case influenced by the extremely depressed cross section of the species, and we therefore do not

introduce a new character state for it. The distribution of this parameter among taxa in our dataset is shown in Additional file 1: Fig. S3C.

Inapplicable if character 13.3 is scored as state 0.

13.3.3. Ventral saddle shape (#27)

0) round
1) angular

Category: Septa
CM: unordered

As lateral lobes are always connected with ventral saddles, this character describes the shape of these saddles. Sharp saddles are defined as sutures that relatively abruptly change their course.

13.3.4. Dorsal saddle shape (#28)

0) round
1) sharp

Category: Septa
CM: unordered

See character 13.3.3.

Inapplicable if character 13.3 is scored as state 0.

14. Suture direction (#29)

0) dorsapertural
1) transverse
2) ventrapertural

Category: Septa
CM: unordered

This character describes the direction of the suture in relation to the central growth axis. The midventral and middorsal points of the sutures will be taken as reference points (see Additional file 1: Fig. S2A).

15. Conch truncation (#30)

0) absent
1) present

Category: General conch shape
CM: unordered

Although there has been some debate, most researchers today acknowledge that conch truncation occurred during life in ascocerids [5]. Although technically not based on a direct observation, this character can alternatively be seen as the mutation that enabled the truncation and was probably the same in all ascocerids, e.g., a highly conical septum of truncation. Conch truncation was also proposed for several post-Ordovician cephalopods unrelated to ascocerids, such as *Sphooceras* and certain oncocerids [17,18], but these are not considered in the current study.

16. Mature conch inflation (#31)

0) absent
1) present

Category: General conch shape
CM: unordered

As with the previous character, this is unique to derived ascocerids and characterizes the sudden inflation of the conch at maturity [1].

17. Ascoceroid septa (#32)

0) absent
1) present

Category: Septa
CM: unordered

This character represents another ascocerid apomorphy and describes the complex folding of their septa [1]. Note that while it would be possible to score ascocerids in more detail, the aim of the present study is to resolve relationships among major groups, thus internal relationships of ascocerids are less important. The monophyly of the Ascocerida (with the possible exception of the Hebetoceratidae) has been firmly established [19].

18. Relative body chamber length (#33)

0) short (≤ 2)
1) medium (> 2 ≤ 4)
2) long (> 4)

Category: Body chamber
CM: ordered (discretised)

The body chamber essentially defines the shape of the soft body. While some studies have compared the length of the body chamber with the total length of the conch, we compare the length of the body chamber with the height of the base of the body chamber. Thus, we calculate the relative body chamber length (RBL) as follows:

RBL = bl/bh

Where bl = body chamber length and bh = body chamber base height. We consider this as biologically more relevant, since it represents whether the soft body was elongated and slender or rather stout. The distribution of this parameter among taxa in our dataset is shown in Additional file 1: Fig. S3D.

19. Adult aperture cross section (#34)

0) more compressed
1) as phragmocone
2) more depressed

Category: Body chamber
CM: unordered

In most taxa, the cross-section shape of the conch is the same in the juvenile as in the adolescent aperture, although there are many exceptions. With this character coding, we make sure that not too much weight is put on the shape of the general cross section, but rather emphasize the ontogenetic change at maturity. As in character 64, compressed conchs are higher than wide, while depressed conchs are wider than high.

20. Adult aperture shape (#35)

0) open
1) contracted

Category: Body chamber
CM: unordered

An open adult aperture has the largest diameter of the conch at or near the peristome. By contrast, a contracted aperture has its largest diameter at some point adapically of the peristome. Note that constricted apertures are coded separately in character 21, since they can occur additionally in otherwise predominantly contracted or open apertures.

20.1. Position of maximum gibbosity (#36)

0) adapical
1) body chamber base
2) adapertural
3) terminal

Category: Body chamber
CM: unordered

The position of maximum gibbosity is the position of the largest conch diameter in taxa with contracted apertures. It defines whether the reduction in shell diameter starts already in the phragmocone (state 0), approximately at the base of the body chamber (state 1), at some point in the body chamber (state 2) or nearly at the aperture (state 3).

Inapplicable if character 20 is scored as state 0.

20.2. Contraction strength (#37)

0) weak (≤ 0.2)
1) medium ($< 0.2 \leq 0.4$)
2) strong (< 0.4)

Category: Body chamber
CM: ordered (discretised)

Contraction strength (CS) represents how strongly the body chamber (and thus the soft body) diminishes in size towards the aperture. Since this is also strongly linked to the length of the body chamber, we calculate this character as the difference between the maximum diameter and the diameter at the aperture, divided by the longitudinal distance of these two points:

$$CS = (mb-ah)/(bl-mp)$$

Where mb = maximum height of body chamber, ah = aperture height, bl = body chamber length, mp = position of maximum gibbosity measured from the body chamber base. Note that mp = 0 if character 20.1 is scored as states 0 or 1, because this character only describes the contraction of the body chamber itself. The distribution of this parameter among taxa in our dataset is shown in Additional file 1: Fig. S3E.

Inapplicable if character 20 is scored as state 0.

20.3. Contraction type (#38)

0) linear
1) non-linear

Category: Body chamber
CM: unordered

If the contraction is linear, the diameter diminishes gradually towards the peristome. If the contraction is non-linear, the rate of contraction increases, creating a convex outline. Linear contraction occurs, for example, in *Actinoceras* Bronn, 1837 [20], while non-linear contraction is present in certain oncocerids [21], although it is most extreme in the Silurian families Trimeroceratidae and Hemiphragmoceratidae [17], which are not included here.

Inapplicable if character 20 is scored as state 0.

21. Apertural constriction (#39)

0) absent
1) present

Category: Body chamber
CM: unordered

In contrast to a contracted body chamber (character 20), a constricted body chamber increases in diameter again after the constriction [1].

22. Body chamber impressions (#40)

0) absent
1) present

Category: Body chamber
CM: unordered

This character describes an autapomorphy of *Orthoceras* Brugière, 1789 and *Ctenoceras* Noetling, 1884. These genera are unique in having peculiar longitudinal imprints on their body chambers [8,22].

23. Nepionic cameral deposits (#41)

0) present
1) absent

Category: Cameral deposits
CM: unordered

Cameral deposits are here regarded as being of organic origin, in agreement with most studies in the past decades [23–30]. However, note that Mutvei [31–33] has repeatedly considered cameral deposits as being formed post-mortem. The distinction between nepionic and ephebic cameral deposits is mostly made for practical reasons, see also detailed explanation to annular deposits (character 37). Knowledge of cameral deposits is quite poor in general; thus, we restrict this character to only a few sub-categories. All subcategories are assigned to nepionic cameral deposits, but they apply also to ephebic cameral deposits.

23.1. Hyposeptal deposits (#42)

0) absent
1) present

Category: Cameral deposits
CM: unordered

Hyposeptal deposits cover the adapical surfaces of the septa [1].

Inapplicable if character 23 is scored as state 0.

23.2. Episeptal deposits (#43)

0) absent
1) present

Category: Cameral deposits
CM: unordered

Episeptal deposits cover the adapertural surfaces of the septa and in advanced stages may produce mural deposits, which cover the mural part of the septum. Episeptal and hyposeptal deposits may contact each other and form a pseudoseptum [1].

Inapplicable if character 23 is scored as state 0.

23.3. Epichoanitic deposits (#44)

0) absent
1) present

Category: Cameral deposits
CM: unordered

Epichoanitic deposits are an autapomorphy of lituitids, in which cameral deposits penetrate or destroy the connecting ring and cover the septal necks [7,25,34,35].

Inapplicable if character 23 is scored as state 0.

24. Ephebic cameral deposits (#45)

0) present
1) absent

Category: Cameral deposits
CM: unordered

See characters 23 and 37 for more details.

25. Septal flap (#46)

0) absent
1) present

Category: Siphuncle
CM: unordered

This character is unique to plectronocerids, in which a part of the dorsal septal neck is straight and extremely elongated into a tongue-shaped structure [36–40]. This feature is indicated in some species by the presence of a "siphuncular bulb" [16,41].

26. Septal neck transition (#47)

0) abrupt
1) gradual

Category: Siphuncle
CM: unordered

The transition between septum and septal necks is not always sharp. In some cases (e.g., in many ellesmerocerids), the septum grades slowly into the neck without sharp boundary (Additional file 1: Fig. S2C). All cases, where the boundary of the septal neck can be more or less clearly discerned, are treated as state 0, although the distinction between the two states is sometimes challenging.

27. Septal neck tip (#48)

0) pointed
1) excavated

Category: Siphuncle
CM: unordered
Remark: controversial

The excavated tip of the septal neck has been documented by Flower [3] in *Protero-cameroceras* and *Adamsoceras*. The reality of this character seems questionable and further research may find it diagenetic or pathologic in origin.

28. Ventral segment adnation (#49)

0) absent
1) present

Category: Siphuncle
CM: unordered

This character is regarded as present, if the connecting ring is adnate to the ventral shell wall. Technically, this would be possible even in dorsal siphuncles if they were widely enough expanded. Due to constructional constraints, this never happens. Nevertheless, there is no logical *a priori* objection to this character state combination. Thus, this character is not treated as subordinate to the siphuncular position.

29. Connecting ring shape (#50)

0) straight
1) annular
2) wedged
3) adapically widened
4) adaperturally widened

Category: Siphuncle
CM: unordered

Different shapes of the connecting ring can be seen in median section [3]. In the simplest case, the connecting ring has a uniform thickness from the adapertural to the adapical end of the segment, the connecting ring is thus considered straight. Annular connecting rings are thickest in the middle part of the segment, while in wedged connecting rings, the middle part is comparatively thin, and the ends thickened. Alternatively, the connecting ring may be either adapically or adaperturally widened.

30. Connecting ring thickness (#51)

0) thin
1) thick

Category: Siphuncle
CM: unordered

The thickness of the connecting ring is often cited as an important character in nautiloid taxonomy. However, there is usually no definition, what constitutes a “thin” or a “thick” connecting ring and furthermore, recrystallisation might impair the comparability between different specimens. Thus, the value of this character – or at least its states that are given in the literature – are sometimes questionable. Here, we apply a conservative approach and define connecting rings as “thin”, when they are thinner or equal in thickness to the septa and as “thick”, when they are thicker than the septa.

31. Connecting ring differentiation (#52)

0) homogenous
1) layered
2) discosorid
3) eyelet

Category: Siphuncle
CM: unordered
Remark: controversial

This character is somewhat questionable, as it is unknown, to what extent diagenesis alters the structure of the connecting ring. It is also unclear, whether thin “homogenous” connecting rings are layered as well, but because the layers are thin, they only seem to be homogenous. The character states are mostly adopted from Flower [3].

32. Connecting ring type (#53)

0) calciosiphonate
1) nautilosiphonate

Category: Siphuncle
CM: unordered

The ultrastructure of the siphuncle was studied in detail by Mutvei [33,39,42–48]. Although there are 13 different structural types of the connecting ring according to him [48], we include only the two main types that he described for his superorders Calciosiphonata

and Nautilosiphonata [47]. The distinction between the two types is the presence of a calcified-perforate inner layer of the connecting ring in calciosiphonate taxa, whereas nautilosiphonates instead supposedly have a fibrous-chitinous inner layer, which is not preserved in fossils. The taxonomic consequences that Mutvei implied based on the structure of the connecting ring are not generally accepted by all nautiloid workers, partly because diagenetic alterations are insufficiently known [30] and partly because he did not provide a comprehensible evolutionary pathway that would phylogenetically justify his classification. Nevertheless, we use this character here and base it on the presence of the calcified-perforate inner layer of the connecting ring. Note that the structure of the connecting ring of discosorids was regarded as unique by Flower & Teichert [49]. However, this could not be confirmed by more recent studies and appears questionable from today's perspective [30,45]. Therefore, the discosorid connecting ring is not included here as a distinct character state.

33. Bullettes (#54)

0) absent
1) present

Category: Siphuncle
CM: unordered

Bullettes are an autapomorphy of derived Discosorida. They consist of a thickened part of the connecting ring around the septal necks [45,49]. We here consider only distinctly enlarged bullettes. Several other taxa have been proposed to contain smaller bullettes, but these may also represent taphonomical artifacts [49,50].

34. Siphuncular diaphragms (#55)

0) absent
1) present

Category: Endoiphuncular deposits
CM: unordered

Although diaphragms have been frequently reported in ellesmerocerids, their morphological structure, variation, composition and function remain poorly studied. It is unclear whether all endosiphuncular deposits have a common origin and whether one type evolved into another. At least for endocones, it has been proposed that they evolved from diaphragms [51]. However, since an independent origin of all endosiphuncular deposits seems equally plausible, all types of deposits are here treated as separate. Compared to endocones and annular deposits, diaphragms are much more poorly studied.

34.1. Diaphragm shape (#56)

0) shallow
1) conical
2) W-shaped

Category: Endoiphuncular deposits
CM: unordered

Diaphragms may have different shapes. We consider diaphragms that are straight to slightly concave as “shallow” and those that are distinctly elongated in adapical direction as “conical”. “W-shaped” diaphragms are present in some Cambrian cephalopods [36]. However, note that there is no sharp transition between these two states. Furthermore, the three-dimensional morphology of these structures is poorly known and the influence of paramedian or oblique sections has not been studied.

Inapplicable if character 34 is scored as state 0.

34.2. Diaphragm spacing (#57)

0) narrow
1) wide

Category: Endoiphuncular deposits
CM: unordered

Diaphragms may be spaced at different intervals. Here, we consider diaphragms that are spaced approximately equal to the septa as “wide” and those that are spaced at smaller intervals as “narrow”.

Inapplicable if character 34 is scored as state 0.

34.3. Diaphragm inclination (#58)

0) directly transverse
1) ventrapertural

Category: Endoiphuncular deposits
CM: unordered

In some cases, the diaphragms are inclined, i.e., in median section, the ventral contact area with the siphuncle is positioned more adaperturally than the dorsal contact area. In other cases, the diaphragms are straight (see Additional file 1: Fig. S2A).

Inapplicable if character 34 is scored as state 0.

35. Endocones (#59)

0) absent
1) present

Category: Endoiphuncular deposits
CM: unordered

Endocones are here defined as conical endosiphuncular structures that are tightly packed. They differ from conical diaphragms in that they are much more closely spaced, more regularly shaped and at least a part of them is perforated at the tip. Flower [16] also suggested that endocones and diaphragms are different in composition, but this has not been tested with modern methods. The endosiphuncular linings found in some members of the Dissidocerida are here not treated as endocones because they are almost parallel to the siphuncle and do not meet in the centre to form an endosiphocone – although it is possible that this happens in the most apical parts of the siphuncle (but to our knowledge has not been demonstrated yet). There is also enough evidence that shows that these linings are probably more closely related to annular deposits [12,52]. Endocone-like structures found in some Discosorida and Actinocerida are explicitly not included here, since those result from adapically prolonged and fused annular deposits.

35.1. Imperforate endocones (#60)

0) absent
1) present

Category: Endoiphuncular deposits
CM: unordered

Taxa which have this character scored as "absent", possess a continuous tube (endosiphotube) through the adapical tips of their endocones. In taxa, which have this character scored as "present", the endosiphotube is traversed by regularly spaced, imperforate endocones. Previous authors have termed these structures "complex" diaphragms or endocones [51,53], which we accept – however, the character is given a different name to represent the character state transition more intuitively. In contrast, taxa with "simple" diaphragms are treated as having diaphragms present (character 34), although it is not entirely clear, whether these are homologous to the diaphragms of ellesmerocerids [51].

Inapplicable if character 35 is scored as state 0.

35.2. Endosiphocone position (#61)

0) ventral
1) central
2) dorsal

Category: Endoiphuncular deposits
CM: unordered

The endosiphocone is defined as the space within the last preserved endocone. This position also applies to the position of the endosiphotube, if present. There may be some ontogenetic change associated with this character, but the extent of this is unclear in many taxa. We therefore only score those taxa as ventral or dorsal, if the endosiphocone is closely positioned to the ventral or dorsal margins of the siphuncle.

Inapplicable if character 35 is scored as state 0.

35.3. Endosiphocone cross section shape (#62)

0) circular
1) crescentic
2) depressed
3) compressed

Category: Endoiphuncular deposits
CM: unordered

This character codes the different shapes of endosiphocones that are seen in cross section. Kröger [54] indicated that there is a large intraspecific variation in this character and thus, its taxonomic use might be questionable. Nevertheless, we include this character here until more studies confirm this suggestion, since it affects only a relatively small number of taxa included in the study. It should also be noted that a crescentic cross section of the endosiphotube may result from protruding large calcareous crests.

Inapplicable if character 35 is scored as state 0.

35.4. Endosiphoblades (#63)

0) absent
1) present

Category: Endoiphuncular deposits
CM: unordered
Remark: controversial

Endosiphoblades longitudinally traverse endocones in radial arrangement. Variations in endosiphoblade number, shape and position are not included here, because they show large (intraspecific?) variation and consequently, their taxonomic relevance and partly even their biological origin have been questioned by some authors [25,51,54,55].

Inapplicable if character 35 is scored as state 0.

35.5. Ventral conchiolin crest (#64)

0) absent
1) present

Category: Endoiphuncular deposits
CM: unordered

Mutvei [55] originally defined the term conchiolin crest. They are longitudinal endosiphuncular structures that protrude from the siphuncular wall into the siphuncle [51]. Conchiolin crests may be positioned ventrally, dorsally, or both. We include both positions as separate characters, because if both are present, they usually differ in size, shape, and/or number. However, ventral crests appear to be always singular and simple in shape, thus only size is defined as a subcharacter. Note that ontogenetic trajectories and intraspecific variation of crests are usually not well documented and future research will doubtlessly refine these characters.

Inapplicable if character 35 is scored as state 0.

35.5.1. Ventral crest size (#65)

0) small
1) large

CM: unordered

Small ventral crests are defined as reaching less than halfway into the siphuncle. In contrast, large ventral crests reach at least close to the centre of the siphuncle and surrounding calcareous crests.

Inapplicable if character 35.5 is scored as state 0.

35.6. Dorsal conchiolin crest (#66)

0) absent

1) present

Category: Endoiphuncular deposits
CM: unordered

See character 35.5 for details on conchiolin crests. While dorsal crests are less common than ventral crests, they appear to be more variable [51].

Inapplicable if character 35 is scored as state 0.

35.6.1. Dorsal crest size (#67)

0) small
1) large

Category: Endoiphuncular deposits
CM: unordered

The same definition as in character 35.5.1 is applied here. In case of multiple differently sized dorsal crests, the size of the largest dorsal crest is taken.

Inapplicable if character 35.6 is scored as state 0.

35.6.2. Dorsal crest number (#68)

0) one
1) two
2) three

Category: Endoiphuncular deposits
CM: unordered

This character codes the number of dorsal crests.

Inapplicable if character 35.6 is scored as state 0.

35.6.3. Dorsal crest shape (#69)

0) simple
1) bifurcating

Category: Endoiphuncular deposits
CM: unordered

This character is mainly used for *Allotrioceras* Flower, 1955, in which the dorsal crest is conspicuously bifurcating. In other taxa, the conchiolin crests are seen as simple straight lines in cross section.

Inapplicable if character 35.6 is scored as state 0.

35.7. Calcareous crests (#70)

0) absent
1) present

Category: Endoiphuncular deposits
CM: unordered

Biologically, the presence of calcareous crests is probably dependent on the presence of conchiolin crests [51]. Nevertheless, as the true biological processes behind the formation of crests are unknown and it is theoretically possible that calcareous crests are developed without conchiolin crests being present, this character is here treated as being on the same hierarchical level as conchiolin crests. However, it should be noted that this coding might lead to unintended weighting (or unaccounted for correlation) of crests in general, since they are to our knowledge invariably correlated. On the other hand, it could be argued that there are at least two genetic changes required for the evolution of crests: 1) the invagination of the siphuncular epithelium and the resulting formation of conchiolin crests and 2) the precipitation of calcareous material on the conchiolin crests, resulting in sharp boundaries between calcareous crests and “regular” endocones. As the position, size, number, and shape of the calcareous crests is directly dependent on the conchiolin crests, we do not code them separately. We consider this as a balanced approach that does not imply too much weight on crests, while still providing accurate description of the morphology and required evolutionary changes.

Inapplicable if character 35 is scored as state 0.

35.7.1. Infula (#71)

0) absent
1) present

Category: Endoiphuncular deposits
CM: unordered

The infula is a term applied to a longitudinal continuous band that is arcuate in cross section and positioned at the boundaries between calcareous crests and endocones [3,51].

Inapplicable if character 35.7 is scored as state 0.

35.8. Lamellar endocones (#72)

0) absent
1) present

Category: Endoiphuncular deposits
CM: unordered
Remark: controversial

This character is restricted to the Intejocerida, in which endocones have characteristic longitudinal lamellae [56,57]. It may be argued that the lamellae represent a multiplication of conchiolin crests, but this has not been demonstrated yet. Furthermore, lamellar endocones may also represent a taphonomical artifact [25].

Inapplicable if character 35 is scored as state 0.

36. Nepionic annular deposits (#73)

0) absent
1) present

Category: Endoiphuncular deposits
CM: unordered

In contrast to endocones, annular deposits originate from the septal necks and grow inwards, as indicated by lamellar growth lines [3,31,58]. From there, they may successively grow larger or fuse with preceding deposits. This indicates that the underlying formation mechanisms are fundamentally different from those of endocones, since only part of the siphuncular epithelium precipitates these deposits. This may be related to the fact that the septal necks are non-permeable and the connecting ring is semi-permeable; subsequent growth would then increasingly block off parts of the connecting ring, enabling more deposits to be precipitated on non-permeable surfaces. In any case, while there may be an underlying common mechanism in the formation of siphuncular deposits, homology of endocones and annular deposits is doubtful at best, we therefore treat them as separate characters on the same hierarchical level. As for annular deposits themselves, detailed studies on their composition and ultrastructure are missing; it is thus not possible to rule out homology of annular deposits found in different taxa, even though they may have arisen independently. Consequently, all deposits that originate from the necks and grow inwards are included here. Note that other types of siphuncular deposits that originate from the septal necks such as parietal or pendant deposits [59] are also included here, since they represent only modifications of annular deposits. Exempt from annular

deposits are the discrete actinosiphonate lamellae of some oncocerids, which do not show growth retardation compared to the chambers and current research indicates that there was only a single depositional cycle continuously along the inside of the connecting ring (see character 40).

36.1. Dorsoventral distribution of annular deposits (#74)

0) ventral
1) equal
2) dorsal

Category: Endoiphuncular deposits
CM: unordered

This character codes whether annular deposits tend to be concentrated on either side of the siphuncle or whether they are more or less symmetrically distributed.

Inapplicable if character 36 is scored as state 0

36.2. Annular deposit extent (#75)

0) septal necks
1) prolonged
2) fusing

Category: Endoiphuncular deposits
CM: unordered

Annular deposits may have different timings in their cessation of growth, resulting in differences in their longitudinal extent within siphuncular segments. Since annular deposits grow progressively less towards the aperture, it is important to assess the maximum extent of the deposits in an apical part of the conch. Taxa with this character scored as septal necks (state 0) have annular deposits that are essentially restricted to the septal necks, while deposits that are in contact with adjacent adapical or adapertural deposits are fusing (state 2). Otherwise, the deposits are considered as prolonged (state 1).

Inapplicable if character 36 is scored as state 0.

36.2.1. Perispatium (#76)

0) absent
1) present

Category: Endoiphuncular deposits
CM: unordered

A perispatium describes the space between prolonged or fused annular deposits that do not touch the connecting ring. If the annular deposits are in contact with the connecting ring, this character is considered as absent (state 0).

Inapplicable if character 36.2 is scored as state 0.

36.2.2. Annular deposit elongation (#77)

0) adapertural
1) symmetric
2) adapical

Category: Endoiphuncular deposits
CM: unordered

By definition, annular deposits that are elongated in adapertural or adapical direction are called parietal deposits (state 0 or 2) [1]. Annular deposits in their original definition apply only to structures that are scored as symmetric here (state 1).

Inapplicable if character 36.2 is scored as state 0.

36.2.3. Engrafts (#78)

0) absent
1) present

Category: Endoiphuncular deposits
CM: unordered

This character represents an autapomorphy of the Wademidae and consists of a series of wedge-shaped structures in the middle of siphuncular segments and adjacent to the connecting ring that are apparently of different composition than the annular deposits [60–62].

Inapplicable if character 36.2 is scored as state 0 or 1.

36.2.4. Canal system (#79)

0) absent
1) present

Category: Endoiphuncular deposits
CM: unordered

Beginning with Teichert [58], who provided the first detailed study of the actinocerid siphuncular morphology, much taxonomic emphasis has been put on the canal system of actinocerids. A canal system consists of a central canal and branching radial canals and is considered an autapomorphy of the Actinocerida. However, a modern revision of these structures including 3-dimensional reconstruction and assessment of diagenetic effects is missing. It is thus possible that future work will change the concept of the canal system in actinocerids.

Inapplicable if character 36.2 is scored as state 0 or 1.

36.2.4.1. Canal system type (#80)

0) dendroid
1) reticulate
2) single-arc
3) double-arc
4) straight

Category: Endoiphuncular deposits
CM: unordered
Remark: controversial

We follow Flower [63] and the Treatise [1] in recognizing five types of radial canals. Dendroid canals consist of numerous branching tubes. Reticulate canals consist of double arcs with complex and irregular branching, mainly concentrated on the central canal. Single-arc [also: curved, 1] canals leave the central canal at the septal foramen and curve towards the apex, meeting the perispatium mid-segment (sometimes these radial canals extend over more than one segment). Double-arc [also: arched, 1] canals are somewhat similar to single-arc canals, but additionally possess a curved canal in adapertural direction. Straight canals are relatively simple and consist of a straight line perpendicular to the central canal and meet the perispatium mid-segment. See Flower [63] and the Treatise [1] for more details and examples. As mentioned above, revision might lead to a different interpretation of these structures.

Inapplicable if character 36.2.4 is scored as state 0.

36.3. Lamellar annular deposits (#81)

0) absent
1) present

Category: Endoiphuncular deposits
CM: unordered

This character is defined as longitudinal ridges on annular deposits. They were mainly described from the actinocerid family Wademidae but are also commonly present in actinocerids [60], although documentation of this feature is often missing in the literature, as it is difficult to recognize in longitudinally sectioned specimens.

Inapplicable if character 36 is scored as state 0.

37. Ephebic annular deposits (#82)

0) absent
1) present

Category: Endoiphuncular deposits
CM: unordered

Technically, this character is dependent on the state of character 36, since siphuncular deposits always grow from the apex towards the aperture, thus a species could only be scored as having ephebic annular deposits present, if nepionic annular deposits are also present. The reasons to code this character not as hierarchically subordinate to character 36 are practical: in many species (especially orthocerids), annular deposits are restricted to apical parts, while some species are only known from more adult fragments. If ephebic annular deposits were to be coded as being hierarchically dependant on the state of nepionic deposits, this would result in a loss of information, since ephebic deposits would have to be scored as "?", if only adult conchs without deposits were known. However, if nepionic deposits were truly absent, scoring ephebic deposits as "absent" would be logically incorrect, since they are dependent on the state of nepionic deposits, i.e., in this case inapplicable. For this reason, we decided to code this character on the same hierarchical level as character 36. In principle, there is also no fundamental logical objection to ephebic deposits being present with nepionic deposits being absent; this character state combination simply never occurred in any taxon (quite possibly because of physiological constraints or selective pressures).

38. Endosiphuncular lining (#83)

0) absent
1) present

Category: Endoiphuncular deposits
CM: unordered

This character is present if a continuous endosiphuncular deposit covers the connecting ring and septal necks, almost parallel to the siphuncle. Although sometimes reminiscent of endocones, they have been shown to more closely resemble elongated and fused annular deposits [12,52]. However, since an independent origin of linings and annuli cannot be excluded, we treat them as separate characters here. In addition, the simultaneous occurrence of annuli, linings and rods in some taxa would make scoring these character combinations challenging.

39. Endosiphuncular rod (#84)

0) absent
1) present

Category: Endoiphuncular deposits
CM: unordered

An endosiphuncular rod consists of a siphuncular deposit that is continuous only along the ventral side of the siphuncle [52]. Evans [12] suggested that rods and annular deposits are derived from endosiphuncular linings by the suppression of the linings in certain areas of the siphuncle. For example, the suppression of the lining on lateral and dorsal sides would lead to a rod, while suppression on the connecting ring would lead to annular deposits. Various combinations of rods, linings and annuli also occur and can be explained with this hypothesis [12]. However, coding the characters in a way that allows for the equal possibility of both this model and a model where rods, linings and annuli evolve independently is challenging. We suggest that the latter model requires less assumptions and that the model of Evans [12] can be assessed *a posteriori* by looking at the character transformations in the resulting phylogenetic tree. A strong phylogenetic signal would probably still lead to an evolutionary sequence of taxa that is congruent with this hypothesis.

40. Actinosiphonate lamellae (#85)

0) absent
1) present

Category: Siphuncle
CM: unordered

These structures have also been described as actinosiphonate deposits [1,3]. Note that despite their name, they are never associated with actinocerids, but are only found in some

but not all Oncocerida. They consist of numerous longitudinally raised lamellae that are directed from the connecting ring towards the central axis of the siphuncle [1]. Whether actinosiphonate lamellae are part of the connecting ring itself or rather represent structurally different deposits that were deposited on the connecting ring is disputed [42,64]. Except for the siphuncular structure of *Octamerella* Teichert & Sweet, 1962, which was studied in detail by Mutvei [42,44,47], the homology, origin, and composition of actinosiphonate lamellae are unclear and require a modern revision. Nevertheless, they are distinct from annular deposits in that they do not progressively diminish in size towards the aperture but are constantly developed throughout the siphuncle, although there may be ontogenetic differences [65].

40.1. Actinosiphonate type (#86)

0) discrete
1) continuous

Category: Siphuncle
CM: unordered

Different structures of actinosiphonate lamellae were described first by Flower [65] and largely followed by the Treatise [1]. Although these authors differentiated between additional types of actinosiphonate lamellae (e.g., bipectinate), we consider their morphology to be insufficiently understood and restrict this character to the two most basic types: discrete and continuous. The latter are developed throughout the entire length of the siphuncle, while discrete lamellae are mainly restricted to the septal necks. The homology of these two types is unclear and distinction between restricted annular deposits and discrete actinosiphonate lamellae may be challenging in some cases.

Inapplicable if character 40 is scored as state 0.

41. Endosiphuncular ridges (#87)

0) absent
1) present

Category: Siphuncle
CM: unordered

This character represents an autapomorphy of the Cyrtocerinida [47,66,67]. Somewhat similar to actinosiphonate lamellae, they consist of raised structures that are directed towards the centre of the siphuncle. However, endosiphuncular ridges are not continuous from one segment to the next but are restricted to a single siphuncular segment [47]. The ridges have been considered as part of the connecting ring but there appears to be a clear

boundary between ridges and connecting ring and they additionally differ in their composition [47]. The extremely thickened connecting rings of many cyrtocerinids are likely homologous to the ridges of *Bathmoceras*, although the detailed ultrastructure of the former has not been investigated [47,66,67].

41.1. Ridge length (#88)

0) short
1) long

CM: unordered

Endosiphuncular ridges vary somewhat in shape. Probably the most significant difference is their length, which is coded in relation to the siphuncular segment, i.e., "short" ridges occupy less than one segment, while "long" ridges span more than one segment.

Inapplicable if character 41 is scored as state 0.

42. Ventral retractor differentiation (#89)

0) absent
1) present

Category: Muscle attachment scars
CM: unordered

Traditionally, muscle scars have been described as one of four types: oncomyarian, pleuromyarian, dorsomyarian or ventromyarian [30,68,69]. While pleuro-, dorso- and ventromyarian describe the position of the main retractors, oncomyarian is rather a description of the number and shape of the retractors. In terms of position, oncomyarian retractors are mostly positioned ventrally, so there is an overlap between oncomyarian and ventromyarian. The position of the retractor muscles does not imply homology or ancestry. On the contrary, it seems likely that ventro- and dorsomyarian retractors are derived from different muscle groups, since the "transitional" form, i.e., pleuromyarian retractors, only appears in derived taxa of the Tarphycerida and the Nautilida [30]. Therefore, a migration of the retractors to the dorsal side did almost certainly not occur and has never been suggested. Instead, dorsal attachments likely correspond to the dorsal aponeurosis in *Nautilus* [1] and are enlarged and became functional retractors in taxa with the dorsomyarian type. The pleuromyarian type can be easily derived from a migration of the ventral retractors [30]. In summary, while these terms have their merits for the description of muscle attachment scars, they do not represent homologous features and are therefore not useful for phylogenetic analysis. Therefore, we employ a different scheme here that puts more emphasis on probable homologous regions of the periphract. Ventral retractor

differentiation describes whether the ventral (or the homologous lateral) retractors are discernible at all. In taxa, which have the character state “absent” (mostly dorsomyarian type), the periphract consists of a continuous band of essentially uniform width with the possible exception of the dorsal aponeurosis.

42.1. Relative ventral retractor width (#90)

0) narrow (≤ 0.3)
1) broad (> 0.3)

Category: Muscle attachment scars
CM: ordered (discretised); effectively unordered

This character (RRW) describes the width of the ventral retractor (rw) relative to the conch height at the base of the body chamber (bh):

RRW = rw/bh

The extent of the retractor is defined as the adapertural lobe of the annular elevation. This means that this character does not describe the width of the main retractors itself, but the width of all retractors that are positioned within the annular lobe. We code this in this way because the annular elevation is often better preserved and individual retractors are commonly difficult to recognize. The annular lobe is thus considered as a homologous character, but with a varying number of retractors. Note that the boundaries of the annular lobe are not always sharp. Since retractors are generally paired, but sometimes the annular elevation shows only a single lobe (fused ventromyarian, see also character 42.4.1), only half of the width of the retractor is taken in such cases, in order to make ventromyarian and pleuromyarian muscle attachments comparable. The distribution of this parameter among taxa in our dataset is shown in Additional file 1: Fig. S3F.

Inapplicable if character 42 is scored as state 0.

42.2. Relative ventral retractor length (#91)

0) short (≤ 0.5)
1) long (> 0.5)

Category: Muscle attachment scars
CM: ordered (discretised); effectively unordered

This character (RRL) describes the length of the retractors in adapical direction (rl) relative to the height at the base of the body chamber (bh):

RRL = rl/bh

The adapical end of the retractors corresponds to the adapical end of the annular elevation, i.e., the last septum. The distribution of this parameter among taxa in our dataset is shown in Additional file 1: Fig. S3G.

Inapplicable if character 42 is scored as state 0.

42.3. Number of retractor pairs (#92)

0) few
1) multiple

Category: Muscle attachment scars
CM: unordered

The exact number of retractors is not always easily discernible because of preservation. However, there are some taxa that possess numerous well-developed muscle attachment scars; these taxa have previously been described as having the oncomyarian type of muscle attachments. Note that we take only the muscle attachment scars on the body chamber itself as evidence for multiple muscle scars, but not longitudinal tracks on the phragmocone, because there is some uncertainty whether they are homologous to oncomyarian muscle attachments [70].

Inapplicable if character 42 is scored as state 0.

42.4. Ventral retractor position (#93)

0) ventral
1) lateral

Category: Muscle attachment scars
CM: unordered

As stated above, we regard the retractors of ventromyarian and pleuromyarian as homologous. Correspondingly, this character describes their position. Note that we regard all retractors as lateral, which have two clearly separated lobes of the annular elevation.

Inapplicable if character 42 is scored as state 0.

42.4.1. Ventral periphract shape (#94)

0) fused

1) bifid

Category: Muscle attachment scars
CM: unordered

Ventromyarian muscle attachments have previously been divided into bifid and non-bifid [30]. We regard the latter as a fused, i.e., there is no discernible midventral indent of the periphract.

Inapplicable if character 42.4 is scored as state 1.

43. Periphract index (#95)

0) narrow (≤ 0.1)
1) broad (> 0.1)

Category: Muscle attachment scars
CM: ordered (discretised); effectively unordered

This character (PPI) essentially describes the minimum width of the annular elevation, i.e., the distance between ventral and dorsal annular lobes in longitudinal direction (= pw), relative to the height at the base of the body chamber (bh):

PPI = pw/bh

The terms periphract and annular elevation have been used interchangeably in the past [1,31]. The distribution of this parameter among taxa in our dataset is shown in Additional file 1: Fig. S3H.

44. Dorsal aponeurosis differentiation (#96)

0) absent
1) present

Category: Muscle attachment scars
CM: unordered

As indicated in character 42, ventral and dorsal retractors are likely not homologous and should be coded separately. The dorsal lobe corresponds to the dorsal aponeurosis in *Nautilus* and is also present in many taxa that were previously considered as ventromyarian [1,71]. Analogous to character 42, taxa that have this character coded as "absent", have an annular elevation of more or less constant width dorsally.

44.1. Relative dorsal aponeurosis width (#97)

0) narrow (≤ 0.3)
1) broad (> 0.3)

Category: Muscle attachment scars
CM: ordered (discretised); effectively unordered

In contrast to the ventral annular lobe, we measure the entire width of the lobe, since the dorsal aponeurosis appears to be never separated into two distant lobes. Other than that, the measurement is identical to character 42.1:

RAW = aw/bh

Where RAW = relative dorsal aponeurosis width, aw = aponeurosis width (in mm) and bh = body chamber base height. The distribution of this parameter among taxa in our dataset is shown in Additional file 1: Fig. S3I.

Inapplicable if character 44 is scored as state 0.

44.2. Relative dorsal aponeurosis length (#98)

0) short (≤ 0.1)
1) long (> 0.1)

Category: Muscle attachment scars
CM: ordered (discretised); effectively unordered

The treatment of this character is identical to character 42.2, but on the dorsal side:

RAL = al/bh

Where RAL = relative dorsal aponeurosis length, al = aponeurosis length (in mm) and bh = body chamber base height. The distribution of this parameter among taxa in our dataset is shown in Additional file 1: Fig. S4A.

Inapplicable if character 44 is scored as state 0.

44.3. Dorsal periphract shape (#99)

0) fused
1) bifid

Category: Muscle attachment scars

CM: unordered

The treatment of this character is identical to character 42.4.1, but on the dorsal side. Since the dorsal aponeurosis is never completely separated, this character does not need an additional hierarchical level.

Inapplicable if character 44 is scored as state 0.

45. Apex shape (#100)

0) blunt
1) conical

Category: Embryonic shell
CM: unordered

The shape of the apex may be categorised into blunt or conical states. The apex is considered to be blunt (state 0), if the conch converges convexly towards the tip of the apex. Contrarily, if the conch converges at a constant angle towards the tip, it is considered a conical (state 1). Spherical apices are here treated as blunt and having a nepionic constriction present (character 54). See also Kröger & Isakar [72] for a discussion of this character.

46. Apex curvature (#101)

0) endogastric
1) straight
2) exogastric

Category: Embryonic shell
CM: unordered

The apex may differ significantly in its curvature from the rest of the conch. We therefore code this character separately.

47. Cicatrix (#102)

0) absent
1) present

Category: Embryonic shell
CM: unordered

The cicatrix is a longitudinal depression on the apical surface of the embryonic conch and is also present in modern *Nautilus* (Stenzel 1964). Largely neglected by earlier workers, the presence of a cicatrix has been credited much phylogenetic value in some more recent studies [72–76]. Nevertheless, the knowledge of the taxonomic distribution of this character is very incomplete, in particular among Ordovician taxa.

48. Initial chamber height (#103)

0) small (< 10 mm)
1) large (≥ 10 mm)

Category: Embryonic shell
CM: ordered (discretised); effectively unordered

The initial size of the conch may vary significantly between different taxa. The diameter is measured at the maximum diameter of the first chamber. In some cases, the embryonic conch is unknown, but conch fragments with very small diameters have been reported, making it safe to assume that the embryonic conch was also small. The distribution of this parameter among taxa in our dataset is shown in Additional file 1: Fig. S4B.

49. Initial chamber length index (#104)

0) short (< 0.5)
1) long (≥ 0.5)

Category: Embryonic shell
CM: ordered (discretised); effectively unordered

The length of the initial conch (il) may significantly differ from the length of subsequent chambers. For this character, it is taken in proportion to the initial chamber diameter (ih; character 48), since this reflects the overall shape of the first chamber.

ILI = il/ih

The initial part of the conch is in some taxa filled entirely by the siphuncle. For consistency, we consider this portion as part of the first chamber. The extent of the initial chamber is therefore from the adapical tip of the conch to the position of the first (adapicalmost) septum. The distribution of this parameter among taxa in our dataset is shown in Additional file 1: Fig. S4C.

50. Caecum position (#105)

0) ventral
1) central

2) dorsal

Category: Embryonic shell
CM: unordered

The caecum is the initial portion of the siphuncle [1]. The position of the siphuncle may change significantly after the initial part of the conch. As the initial conch is generally small, this character is not divided into as many states as the conch in later ontogenetic stages (character 67). In the cases where the caecum entirely fills the initial chamber (see character 51), the position of the first septal foramen is taken.

51. Pre-septal cone (#106)

0) absent
1) present

Category: Embryonic shell
CM: unordered

In some cases, the initial chamber is entirely occupied by the siphuncle. This type is commonly referred to as the *Nanno*-type apex [1] and here treated as the presence of a pre-septal cone [51].

51.1. Initial siphuncle index (#107)

0) small (< 0.25)
1) moderate (≥ 0.25 < 0.75)
2) large (≥ 0.75)

Category: Embryonic shell
CM: ordered (discretised)

This character represents the size of the initial part of the siphuncle or caecum (is) as compared to the height of the initial conch:

$$ISI = is/ih$$

Where ISI = initial siphuncle index. Since a pre-septal cone entirely fills the initial chamber, this character is inapplicable for these apices. The distribution of this parameter among taxa in our dataset is shown in Additional file 1: Fig. S4D.

Inapplicable if character 51 is scored as state 1.

51.2. Caecum attachment (#108)

0) free
1) direct

Category: Embryonic shell
CM: unordered

This character codes whether the initial siphuncular segment is directly attached to the apical end of the conch or whether it ends freely [1]. The attachment of the siphuncle by means of a prosiphon as in ammonoids is unknown in nautiloids [1]. Because a siphuncle that entirely occupies the initial chamber is inherently directly "attached" to the shell wall of the initial chamber, this character is only logically applicable if no pre-septal cone is present.

Inapplicable if character 51 is scored as state 1.

52. Caecum shape (#109)

0) tubular
1) bulbous

Category: Embryonic shell
CM: unordered

The caecum may have different shapes. In some cases, it is distinctly expanded, while in others, the siphuncle is essentially tubular from the earliest stages on.

53. Apex ornamentation (#110)

0) smooth
1) longitudinal
2) transverse
3) reticulate

Category: Embryonic shell
CM: unordered

The ornamentation of the apex may differ from the ornamentation of the adult conch [74,76]. Since apex characters are known only in a limited number of species, we restrict the coding of its ornamentation to only one character, in contrast to the adult conch (see characters 1–9). There is also less variability in this character compared to the adult conch.

54. Nepionic constriction (#111)

0) absent
1) present

Category: Embryonic shell
CM: unordered

The nepionic constriction is a distinct transverse groove near the apex of the conch, which is usually interpreted as the timing of hatching [77,78]. Commonly, this character can be seen only in well-preserved specimens, but this applies almost in general to the embryonic conch.

55. Ontogeny siphuncle diameter (#112)

0) narrowing
1) constant
2) widening

Category: Ontogeny
CM: unordered

The relative diameter of the siphuncle changes markedly during ontogeny in some taxa [1,4]. Since this usually applies to both the septal foramen and the segment, we code only one character. Note that while character 69 describes the size of the siphuncle at a nearly adult stage without taking abrupt adult modifications into account, these are explicitly included here. Nevertheless, we do not distinguish between different rates of ontogenetic changes.

56. Ontogeny siphuncle position (#113)

0) ventrad
1) constant
2) dorsad

Category: Ontogeny
CM: unordered

Ontogenetic changes may also occur in the position of the siphuncle. This character describes the direction of this ontogenetic change.

57. Ontogeny septal neck shape (#114)

0) increasing curvature
1) constant curvature

2) decreasing curvature

Category: Ontogeny
CM: unordered

During ontogeny, septal necks may change from cyrtochoanitic to orthochoanitic or vice-versa. The former case is here coded as increasing curvature (state 0) and the latter as decreasing curvature (state 2) of the septal necks.

58. Ontogeny cameral length (#115)

0) decreasing
1) constant
2) increasing

Category: Ontogeny
CM: unordered

In many taxa, relative cameral length exhibits considerable variability, both intraspecific and within single phragmocones [12,25,54,79]. In this character, we consider only distinct trends during the ontogeny of cameral lengths. Cases that show only a slight decrease or increase are considered as constant. Note that ontogenetic trends have not been investigated for many taxa and reinvestigating them for all studied taxa is out of the scope of this study. Therefore, for many taxa this character is based on a rather slim dataset. Septal crowding in mature specimens is not included here. Taxa with irregular changes in cameral length but without obvious trend are treated as constant.

59. Ontogeny expansion rate (#116)

0) decreasing
1) constant
2) increasing

Category: Ontogeny
CM: unordered

Intraspecific variability and ontogenetic change of the conch expansion rate are similar in magnitude to character 58. As in that character, we consider only distinct trends or marked changes. Note that adult modifications such as constrictions and contractions are already covered by characters 16, 20 and 21. However, these can be seen as special cases of decreasing expansion rates, as in all three cases the expansion rate becomes negative. Furthermore, these are explicit adult modifications and therefore, the separate treatment of these characters is justified in our opinion. Nevertheless, this has the side effect that all

taxa that have a constriction or contraction are invariably scored with state 0 (decreasing) for this character as well.

60. Maximum conch size (#117)

0) very small (≤ 0)
1) small (> 0 ≤ 0.75)
2) moderate (> 0.75 ≤ 1.75)
3) large (> 1.75)

Category: General conch shape
CM: ordered (discretised)

This character essentially represents body size. The maximum diameter of the conch (MCS) is an approximate representation of the size of the soft body. However, as some species are strongly compressed or strongly depressed, we use the square-root of the product of maximum conch height (mh) and maximum conch width (mw) as an average size representation:

$$MCS = \log \sqrt{(mh*mw)}$$

We use the maximum size of reported specimens. Note that this may be more accurate in species with high numbers of available material and species that are only represented by a single specimen may show smaller size than their true largest representatives. Body size varies between tiny Cambrian forms with diameters of only about 4 mm [80] to giant endocerids with diameters of almost 300 mm [81]. These large differences justify the inclusion of this character in the phylogenetic analysis. The log-distribution of the entire dataset shown in Additional file 1: Fig. S4E is close to a normal distribution, the cut-off values are therefore chosen relatively arbitrarily at 0, 0.75 and 1.75, which correspond in a circular conch to an approximate cross section of 11 mm, 27 mm and 85 mm, respectively. The distribution of this parameter among taxa in our dataset is shown in Additional file 1: Fig. S4E.

61. Adult phragmocone form (#118)

0) endo-cyrtocone
1) orthocone
2) exo-cyrtocone
3) gyrocone
4) coiled evolute
5) coiled involute

Category: General conch shape
CM: ordered

This character mainly describes the conch curvature, since this is a clear morphocline that can be treated as an ordered character. Although terms such as breviconic or longiconic have also been applied, these are mainly related to expansion (character 63). As conch curvature may drastically change during ontogeny (most notably in lituitids), we code the adult and juvenile conch separately (see character 62). Endo-cyrtocones have a concave venter and a convex dorsum, orthocones are straight, exo-cyrtocones have a convex venter and a concave dorsum, gyrocones are more strongly curved and consist of several whorls that are not in contact with each other, evolutes have whorls in contact but without an imprint zone and involutes are so strongly coiled that they develop an imprint zone. Note that curvature partly depends on expansion rate and the boundaries are chosen rather arbitrarily. For orientation of the ventral and dorsal side see introductory remarks at the beginning of the appendix.

61.1. Divergent body chamber (#119)

0) absent
1) present

Category: Body chamber
CM: unordered

A divergent body chamber may occur in coiled conchs. In such cases, the body chamber or part of the last whorl is not in contact with the preceding whorl [82,83].

Inapplicable if character 61 is scored as states 0-3.

62. Juvenile phragmocone form (#120)

0) endo-cyrtocone
1) orthocone
2) exo-cyrtocone
3) gyrocone
4) evolute
5) involute

Category: General conch shape
CM: ordered

This character is scored in accordance with character 61. Note that this character does not include the shape of the apex, but the general shape of the phragmocone of juvenile to sub-adult ontogenetic stages. The boundary between juvenile and adult phragmocone are drawn somewhat arbitrarily, but as a rule of thumb, if there is a difference in curvature of those parts of the phragmocone that are closer to the aperture when compared to those

closer to the apex, they should be scored differently. Ultimately there is no satisfactory quantitative description of curvature alone, since it is also dependent on expansion rate. However, a rough idea can be obtained by comparing the relation between the distance of the septa measured directly on the venter when compared to those on the dorsum. This is only comparable when expansion rate is roughly the same in both measurements and it cannot differentiate between gyroconic, evolute and involute conch forms.

62.1. Umbilical perforation (#121)

0) small
1) large

Category: General conch shape
CM: unordered

An umbilical perforation is present if there is an open space in the coiling axis within the innermost whorls in coiled conchs [1]. At least a very small umbilical perforation is probably always present. Here, we consider those that are at least twice the size of the initial chamber as large (state 1).

Inapplicable if character 62 is scored as states 0-3.

63. Expansion rate (#122)

0) slow (<10°)
1) moderate (≥ 10° < 20°)
2) rapid (≥ 20°)

Category: General conch shape
CM: ordered (discretised)

Expansion rate is one of the basic characters of the overall conch shape, yet several factors hamper its comparability across taxa. First, the methodology differs between authors and in many cases, it is not even provided. In orthocones, it is possible to measure the angle directly; however, since most specimens are broken at their apical end and thus do not provide their endpoint, the application of a standard goniometer is challenging. Alternatively, the angle may be calculated, although measuring errors might have a large effect, especially in short fragments with a small expansion rate [8,84]. In several studies, the expansion rate is given as the distance per 1 mm diameter increase [36,85–87]. It is possible to convert this expansion rate into angular measurements in degree; however, this results in large error margins when the angle is large and the distance consequently short. Besides, both scales are not directly comparable because angular data is linear in contrast to the distance per 1 mm diameter increase, which was mainly used in older Chinese publications. A second issue is that the expansion rate may change during ontogeny (see

character 59) and measuring or calculating it across a longer distance of the conch may obscure ontogenetic differences. Third, curved conchs are problematic to measure, since their ventral and dorsal margins are not straight. Lastly, the expansion rate may not be the same laterally and dorsoventrally. This will always be the case if we assume a constant conch width index (character 64) other than 1.0, i.e., either a compressed or depressed cross section. Consequently, data from the literature had to be always double-checked and supplemented with own measurements. These measurements were done either from the authors' photographs or published figures in a computer vector drawing program. In order to make the results as comparable as possible, we mostly took measurements of the lateral expansion rate, since this is independent of curvature. In cases where the cross section was essentially circular, it was also acceptable to measure the dorsoventral expansion rate. In a few curved specimens it was only possible to measure them in lateral view (because of incomplete preservation or lacking figures); in these cases, we took the angle in degrees between the tangent lines through opposite points on venter and dorsum that lie perpendicular to the central growth axis. The distribution of this parameter among taxa in our dataset is shown in Additional file 1: Fig. S4F.

64. Conch width index (#123)

0) strongly compressed (≤ 0.7)
1) slightly compressed ($> 0.7 < 0.95$)
2) circular ($\geq 0.95 \leq 1.05$)
3) slightly depressed ($> 1.05 < 2.0$)
4) strongly depressed (≥ 2.0)

Category: General conch shape
CM: ordered (discretised)

This character describes the shape of the cross section as proportion between conch width and conch height. The parameter essentially corresponds to the whorl width index (WWI) commonly used for ammonoids [88]; however, the term "whorl" is obviously inaccurate for many nautiloids, which commonly do not have coiled conchs consisting of whorls. It is calculated as follows:

$$CWI = ch/cw$$

Where CWI = conch width index, ch = conch height (in mm) and cw = conch width (in mm). The distribution of this parameter among taxa in our dataset is shown in Additional file 1: Fig. S4G.

65. Relative cameral length (#124)

0) extremely short (< 0.1)
1) short ($\geq 0.1 < 0.2$)

2) moderate ($\geq 0.2 < 0.4$)
3) long (≥ 0.4)

Category: Septa
CM: ordered (discretised)

Although cameral length is often variable within a single conch, there are clear differences between taxa, suggesting a strong phylogenetic signal. As an example, many Cambrian plectronocerids possess extremely short chambers [36], while some orthoceratoids exhibit much longer chambers [8,14]. This character is essentially taken from Kröger & Aubrechtová [89], with the difference that it is called here "relative cameral length" (RCL) instead of "relative cameral height". The latter is misleading because it concerns a character in longitudinal direction, while "height" applies to the dorsoventral direction [90]. Accordingly, it is calculated as follows:

RCL = cl/ch

Where cl = cameral length and ch = conch height. The distribution of this parameter among taxa in our dataset is shown in Additional file 1: Fig. S4H.

66. Septal concavity index (#125)

0) shallow (≤ 0.15)
1) moderate ($> 0.15 \leq 0.25$)
2) deep (> 0.25)

Category: Septa
CM: ordered (discretised)

The septal concavity index (SCI) is the length of the free part of the septum in adapical direction (sc) in proportion to the corresponding conch height (ch).

SCI = sc/ch

This character has rarely been used in the taxonomy of nautiloids [29,90]. The distribution of this parameter among taxa in our dataset is shown in Additional file 1: Fig. S4I.

67. Relative septal foramen position (#126)

0) ventral (< 0.1)
1) subventral ($\geq 0.1 < 0.4$)
2) central ($\geq 0.4 < 0.6$)
3) subdorsal ($\geq 0.6 < 0.7$)
4) dorsal (≥ 0.7)

Category: Siphuncle
CM: ordered (discretised)

The position of the siphuncle is an important taxonomic criterion. However, there have been different definitions of how to measure this position. The size of the siphuncle and the segment expansion may also play a role. Aronoff [90] defined it as the distance between the segment axis and the venter, measured perpendicular to the latter, proportional to the corresponding conch diameter (segment position ratio = SPR). Note that this method theoretically could lead to SPR > 1.0, because the venter is not parallel to the central conch axis. This approach was followed for example in Frey [91] and slightly modified by Evans [12], while in other publications, it becomes evident that – although often not explicitly stated – the distance was probably taken from the shell wall to the siphuncular wall (either septal neck or connecting ring) perpendicular to the central growth axis [89,92,93]. In older publications, the position of the siphuncle is often only given as a relative term (see state names of this character), without giving a quantitative measure. While all these methods have their own advantages and disadvantages and probably lead to very similar results, it is important to state a precise definition of this character that is comparable across taxa and as independent as possible from other characters such as siphuncle size and segment shape. With Aronoff's [90] approach, a siphuncle would never be marginal and larger siphuncles would tend to show a more central position, even if the connecting ring is close to the shell wall. On the other hand, using the siphuncular wall instead of the central siphuncular axis would tend to show more ventrally positioned large siphuncles, even if they are perfectly central. Thus, we propose a new definition that is independent of the size of the siphuncle. We took the distance between the ventral shell wall and the septal foramen, perpendicular to the central conch axis and divided this by the conch height minus the diameter of the septal foramen. As a formula, this relation is expressed as

RSP = sv/(ch-fh)

Where sv = distance of the septal foramen from the ventral shell wall, ch = conch height and fh = siphuncle height at the foramen. It is equivalent to sv/(sv+sd), where d is the dorsal distance of the septal foramen. Using this approach has the advantage of producing a ratio that can vary in principle between 0 (ventral marginal) and 1 (dorsal marginal), thus giving an intuitive and direct representation of the position that is independent from the size of the siphuncle or the shape of the segment. The distribution of this parameter among taxa in our dataset is shown in Additional file 1: Fig. S5A.

67.1. Ventral foramen adnation (#127)

0) absent
1) present

Category: Siphuncle
CM: unordered

If present in a marginal siphuncle, the septal foramen is adnate to the shell wall, i.e., the siphuncle lies completely marginal and the septal necks are not visible mid-ventrally. The decision to code this separately from the siphuncular position is justified by the difference in construction of the septal neck in a ventrally open foramen, since it is not continuous throughout the 360° around the siphuncle as in more centrally positioned siphuncles. Furthermore, there are also practical reasons: it is not always clear whether a septal foramen is contiguous to the shell wall, because of imperfect preservation or sectioning; this would result in many cases where the character state would be unknown. Also, in terms of position the difference between "marginal" and "submarginal" siphuncles is minimal and using these as different character states in character 67 might put too much weight on it compared to other character states. Nevertheless, this small difference comes with constructional implications because septal necks and connecting ring are continuous with the shell wall. The coding employed here puts more emphasis on the switch between submarginal and marginal character states, while switches between either of these two and other states are treated equally.

Inapplicable if character 67 is scored as state 1-4.

67.2. Siphuncular suture (#128)

0) saddle
1) straight
2) lobe

Category: Siphuncle
CM: unordered
Remark: controversial

In taxa with ventral siphuncles, the sutures may show distinct ventral lobes or saddles that are not part of the generally straight or sinuous suture (i.e., there is an abrupt change in the course of the suture line). This feature has been interpreted as being a taphonomic consequence of the close proximity of the siphuncle to the shell wall [12–14]. Nevertheless, the variation of this character between taxa with both saddles and lobes [12,47] suggests that there is also some genetic control of this character. In each case it should be carefully considered if and how the specimens were taphonomically altered.

Inapplicable if character 67 is scored as states 1-4.

68. Siphuncle width index (#129)

0) compressed (≤ 0.9)
1) circular (> 0.9 < 1.1)
2) depressed (≥ 1.1)

Category: Siphuncle
CM: ordered (discretised)

In most cases, the siphuncle is circular in cross-section. However, there are exceptions, although these are not always clearly indicated in the literature. Therefore, there is some uncertainty in the distribution of this character among taxa. Note that ventral circular siphuncles may appear compressed on the inclined surface of the septum. For this reason, we measure the siphuncle width and height perpendicular to its growth axis. The calculation of this index (SWI) is as follows:

SWI = fh/fw

Where fh = septal foramen height and fw = septal foramen width. The distribution of this parameter among taxa in our dataset is shown in Additional file 1: Fig. S5B.

69. Relative septal foramen height (#130)

0) narrow (< 0.15)
1) medium (≥ 0.15 < 0.3)
2) wide (≥ 0.3)

Category: Siphuncle
CM: ordered (discretised)

The size of the siphuncle varies strongly between taxa and has long been considered to be important for phylogenetic classification [1,25,30,94,95]. Here, we take the ratio of the height of the septal foramen (fh) to the conch height (ch) as a representative character:

RSH = fh/ch

Kröger & Aubrechtová [89] used the term "relative siphuncular diameter" with the same definition; however, we believe that the term employed here is more precise. The distribution of this parameter among taxa in our dataset is shown in Additional file 1: Fig. S5C.

70. Siphuncle compression ratio (#131)

0) elongated (< 0.8)
1) quadratic (≥ 0.8 < 1.2)
2) slightly broadened (≥ 1.2 < 3)

3) strongly broadened (≥ 3)

Category: Siphuncle
CM: ordered (discretised)

This character is adopted from Aronoff [90], who defined it as the ratio between segment height and segment length. Note that he excluded the septal necks from the segment length. While this may be applicable in species with septal necks of similarly short length, it would produce negative values in certain endocerids that are characterized by macrochoanitic septal necks. We therefore explicitly include the septal necks in the siphuncular segment to make this character comparable across taxa. Also note that segment length is not necessarily equal to cameral length depending on expansion rate, conch curvature and siphuncle position. The calculation of the Siphuncle compression ratio (SCR) is as follows:

SCR = sh/sl

Where sh = siphuncular segment height and sl = siphuncular segment length. The distribution of this parameter among taxa in our dataset is shown in Additional file 1: Fig. S5D.

71. Relative siphuncular shape (#132)

0) concave (< 0.95)
1) tubular (≥ 0.95 < 1.05)
2) slightly expanded (≥ 1.05 < 1.5)
3) strongly expanded (> 1.5)

Category: Siphuncle
CM: ordered (discretised)

The shape of the siphuncular segments is highly variable across taxa. This shape can almost entirely be described by this character, which is the relation between the mid-segment height of the siphuncle (sh) and the height at the septal foramen (fh). Depending on the shape of the connecting ring, mid-segment height is measured at the point where the siphuncle has its minimum (concave segments) or maximum (convex segments) height. The Relative siphuncular shape (RSS) is thus calculated with the following formula:

RSS = sh/fh

The connecting ring is excluded from the segment because its shape is already described in character 29. A similar ratio was defined by Aronoff [90] as “constriction ratio” and by Kröger & Aubrechtová [89] also as “relative siphuncular shape”. However, both studies defined it as the reciprocal value (foramen height/segment height). We argue that using segment height/ foramen height is more intuitive, because siphuncles with convex

segments are usually referred to as "expanded" or "widened", implying larger values. The distribution of this parameter among taxa in our dataset is shown in Additional file 1: Fig. S5E.

71.1. Adapical segment adnation (#133)

0) absent
1) present

Category: Siphuncle
CM: unordered

Similar to character 72.1.1, but on the opposite side, i.e., the adapical end of the connecting ring is touching the adapertural surface of the preceding septum. Traditionally, "area of adnation" refers mainly to this character [1].

Inapplicable if character 71 is scored as state 0 or 1.

71.2. Siphuncular segment shape (#134)

0) straight
1) biconvex
2) planoconvex

Category: Siphuncle
CM: unordered

This character describes the shape of the siphuncular segment. Although it is somewhat overlapping with character 71 (relative siphuncular shape), we consider these two characters as distinct, because, for example, straight siphuncular segments may also be slightly constricted at the septal foramen or planoconvex segments cannot be described by their relative siphuncular shape alone.

Inapplicable if character 71 is scored as state 0 or 1.

71.2.1. Position of maximum segment height (#135)

0) adapertural
1) middle
2) adapical

Category: Siphuncle
CM: unordered

This character describes the shape of convex siphuncular segments in more detail. In the case of biconvex segments, terms such as “amphora-shaped” (state 0) or “globular” (state 1) have been applied.

Inapplicable if character 71.2 is scored as state 0 or 1.

71.2.2. Position of highest segment convexity (#136)

0) ventral
1) equal
2) dorsal

Category: Siphuncle
CM: unordered

This character describes whether convex siphuncular segments are expanded more towards the venter, dorsum or equally.

Inapplicable if character 71.2 is scored as state 0 or 1.

72. Septal neck index (#137)

0) achoanitic (= 0.0)
1) microchoanitic (> 0.0 ≤ 0.2)
2) mesochoanitic (> 0.2 ≤ 0.4)
3) hemichoanitic (> 0.4 ≤ 0.8)
4) holochoanitic (> 0.8 ≤ 1.2)
5) macrochoanitic (> 1.2)

Category: Siphuncle
CM: ordered (discretised)

Many different terms have been applied to the shape of the septal necks; nine names are listed in the Treatise [1]. However, these terms do not imply homology, because some refer to the length (e.g., holochoanitic), while others refer to the shape of the septal necks (e.g., cyrtochoanitic). Therefore, we divide them into two categories. This character represents the first category, which only describes the length of the septal necks. The septal neck index (SNI) represents the ratio between the length of the septal neck (nl) and the siphuncular segment (sl), measured parallel to the siphuncular axis [1,29]:

SNI = nl/sl

The terms microchoanitic and mesochoanitic are here newly introduced. The distribution of this parameter among taxa in our dataset is shown in Additional file 1: Fig. S5F.

72.1. Septal neck shape (#138)

0) loxochoanitic
1) orthochoanitic
2) suborthochoanitic
3) cyrtochoanitic

Category: Siphuncle
CM: ordered

This character describes the shape of the septal necks. Some septal necks are extremely short, making differentiation between orthochoanitic or cyrtochoanitic septal necks virtually impossible [72]. Therefore, we treat this character as inapplicable, if the septal necks are extremely short or absent (achoanitic). The definitions of the terms loxo-, ortho-, subortho- and cyrtochoanitic follow Kröger & Isakar [72] and refer to the angle of the septal necks compared to the siphuncle axis. We do not use the term "recumbent", because this can be scored as cyrtochoanitic in addition to the presence of adaperturally adnate connecting rings (character 72.1.1, state 1) and a high brim-neck ratio (character 72.1.2, state 2).

Inapplicable if character 72 is scored as state 0.

72.1.1. Adapertural segment adnation (#139)

0) absent
1) present

Category: Siphuncle
CM: unordered

Siphuncular segments may be adaperturally adnate in some taxa, i.e. the adapertural end of the connecting ring is touching the adapical surface of the following septum. Logically, the septal necks must be cyrtochoanitic in such cases and they are typically termed "recumbent" [1].

Inapplicable if character 72.1 is scored as state 0-2.

72.1.2. Brim-neck ratio (#140)

0) necks longer (< 0.9)
1) equal (≥ 0.9 ≤ 1.1)
2) brims longer (> 1.1)

Category: Siphuncle
CM: ordered (discretised)

The shape of cyrtochoanitic septal necks can be further characterised by the ratio between the height of the brims (br) and the length of the necks (nl) as the brim-neck ratio (BNR):

BNR = br/nl

Brims are defined as the recurved part of the septal necks that run parallel to the septum [1]. The distribution of this parameter among taxa in our dataset is shown in Additional file 1: Fig. S5G.

Inapplicable if character 72.1 is scored as state 0-2.

72.2. Neck inward projection (#141)

0) absent
1) present

Category: Siphuncle
CM: unordered

This character refers to a modification of the distal neck that consists of a projection towards the inside of the siphuncle. Some necks of this type have been termed sub-holochoanitic, although only if they were slightly less than holochoanitic in length [1]. However, similar modifications of the septal necks also occur in shorter necks, e.g., in *Bathmoceras* Barrande, 1867 [47,96]. Some macrochoanitic septal necks that are inclined inwards when they overlap preceding septal necks are also added to this category, although their inclusion may be doubtful. This character only occurs in orthochoanitic septal necks; nevertheless, it is logically also applicable to loxo- or subortho- or cyrtochoanitic necks. Only achoanitc septal necks are excluded from this character.

Inapplicable if character 72 is scored as state 0.

Text S2: Character sets

Below, we list all characters that were excluded from the complete list of characters in different analyses. Only one analysis included all characters (CoCo). For all other analyses, controversial characters were excluded as well, though they are not explicitly listed here to make the character categories clearer. Abbreviations are given in Table 1 of the main article. Values in brackets denote relevant exclusion criteria. The character sets Cr, Ma and CM represent speculatively scored connecting ring type (character 32), muscle attachments (characters 42-44) or both. See nexus files included in Additional file 5: Data S4 for details.

Ct: Excluding 6 controversial characters:

- 27. Septal neck tip
- 31. Connecting ring differentiation
- 35.4. Endosiphoblades
- 35.8. Lamellar endocones
- 36.2.4.1. Canal system type
- 67.2. Siphuncular suture

Ic: Excluding 23 incomplete characters (>75% missing data):

- 32. Connecting ring type (87%)
- 42. Ventral retractor differentiation (82%)
- 42.1. Relative ventral retractor width (83%)
- 42.2. Relative ventral retractor length (82%)
- 42.3. Number of retractor pairs (83%)
- 42.4. Ventral retractor position (82%)
- 42.4.1. Ventral periphract shape (83%)
- 43. Periphract width index (83%)
- 44. Dorsal aponeurosis differentiation (83%)
- 44.1. Relative dorsal aponeurosis width (83%)
- 44.2. Relative dorsal aponeurosis length (83%)
- 44.3. Relative dorsal periphract shape (83%)
- 45. Apex shape (82%)
- 46. Apex curvature (78%)
- 47. Cicatrix (90%)
- 49. Initial chamber length index (88%)
- 50. Caecum position (86%)
- 51. Pre-septal cone (78%)
- 51.1. Initial siphuncle index (88%)
- 51.2. Caecum attachment (91%)
- 52. Caecum shape (90%)
- 53. Apex ornamentation (91%)
- 54. Nepionic constriction (83%)

Am: Excluding 13 autapomorphic characters:

- 13.3.4. Dorsal saddle shape

- 17. Ascoceroid septa
- 23.2. Episeptal deposits
- 35.5.1. Ventral crest size
- 35.6.1. Dorsal crest size
- 35.6.2. Dorsal crest number
- 35.6.3. Dorsal crest shape
- 36.2.3. Engrafts
- 36.3. Lamellar annular deposits
- 40.1. Actinosiphonate type
- 41.1. Ridge length
- 42.4. Ventral retractor position
- 44.3. Dorsal periphract shape

<u>Ia: Excluding 65 inapplicable characters (>25% gaps):</u>

- 5.1. Relative hyponomic sinus length (57%)
- 5.2. Relative hyponomic sinus width (57%)
- 5.3. Hyponomic sinus shape (57%)
- 8.1. Annulation direction (87%)
- 8.2. Annulation distance (85%)
- 9.1. Longitudinal element strength (90%)
- 9.2. Longitudinal element distance (90%)
- 9.3. Longitudinal element variation (90%)
- 13.1. Ventral lobe (62%)
- 13.2. Dorsal lobe (62%)
- 13.3. Lateral lobes (62%)
- 13.3.1. Lateral lobe position (72%)
- 13.3.2. Lateral lobe depth (72%)
- 13.3.3. Ventral saddle shape (73%)
- 13.3.4. Dorsal saddle shape (73%)
- 20.1. Position of maximum gibbosity (73%)
- 20.2. Contraction strength (73%)
- 20.3. Contraction type (73%)
- 23.1. Hyposeptal deposits (72%)
- 23.2. Episeptal deposits (72%)
- 23.3. Epichoanitic deposits (72%)
- 34.1. Diaphragm shape (81%)
- 34.2. Diaphragm spacing (83%)
- 34.3. Diaphragm inclination (81%)
- 35.1. Imperforate endocones (90%)
- 35.2. Endosiphocone position (90%)
- 35.3. Endosiphocone cross section (90%)
- 35.4. Endosiphoblades (91%)
- 35.5. Ventral conchiolin crests (91%)
- 35.5.1. Ventral crest size (97%)
- 35.6. Dorsal conchiolin crest (91%)

- 35.6.1. Dorsal crest size (98%)
- 35.6.2. Dorsal crest number (98%)
- 35.6.3. Dorsal crest shape (98%)
- 35.7. Calcareous crests (91%)
- 35.7.1. Infula (91%)
- 35.8. Lamellar endocones (89%)
- 36.1. Dorsoventral annular deposit distribution (79%)
- 36.2. Annular deposit extent (79%)
- 36.2.1. Perispatium (84%)
- 36.2.2. Annular deposit elongation (84%)
- 36.2.3. Engrafts (91%)
- 36.2.4. Canal system (91%)
- 36.2.4.1. Canal system type (92%)
- 36.3. Lamellar annular deposits (79%)
- 40.1. Actinosiphonate type (99%)
- 41.1. Ridge length (94%)
- 42.1. Relative ventral retractor width (33%)
- 42.2. Relative ventral retractor length (32%)
- 42.3. Number of retractor pairs (33%)
- 42.4. Ventral retractor position (32%)
- 42.4.1. Venral periphract shape (38%)
- 44.1. Relative dorsal aponeurosis width (63%)
- 44.2. Relative dorsal aponeurosis length (63%)
- 44.3. Dorsal periphract shape (63%)
- 61.1. Divergent body chamber (91%)
- 62.1. Umbilical perforation (90%)
- 67.1. Ventral foramen adnation (54%)
- 67.2. Siphuncular suture (60%)
- 71.1. Adapical segment adnation (60%)
- 71.2. Siphuncular segment shape (60%)
- 71.2.1. Position of maximum segment height (67%)
- 71.2.2. Position of maximum segment convexity (67%)
- 72.1.1. Adapertural segment adnation (83%)
- 72.1.2. Brim-neck ratio (83%)

Text S3: Taxon sets

Below, we list all taxa that were excluded from the complete list of taxa in different analyses. Values in brackets denote relevant exclusion criteria. For the complete list of taxa, references, and stratigraphic positions, see Additional file 2: Data S1. Abbreviations are given in Table 1.

Dp: Excluding 4 pseudoduplicate taxa:

- *Bactroceras mourguesi*
- *Bassleroeras perseus*
- *Ectenolites primus*
- *Sinoceras fenxiangense*

Ic: Excluding 38 incomplete taxa (>40% missing data):

- *Aetheloxoceras suxianense* (53%)
- *Allotrioceras bifurcatum* (64%)
- *Bajkaloceras angarense* (47%)
- *Bisonoceras corniforme* (50%)
- *Cabaneroceras aznari* (68%)
- *Cacheoceras unimodum* (63%)
- *Centrocyrtocerina frizonensis* (47%)
- *Clelandoceras rarum* (54%)
- *Clytoceras capax* (44%)
- *Cyclorangeroceras blakei* (44%)
- *Dwightoceras dactyloides* (56%)
- *Eburoceras jiagouense* (43%)
- *Eothinoceras maitlandi* (43%)
- *Exoclitendoceras rochdalense* (43%)
- *Glenisteroceras obscurum* (62%)
- *Gouldoceras synchonena* (47%)
- *Huaihecerina elegans* (56%)
- *Intejoceras angarense* (47%)
- *Lebetoceras oepiki* (54%)
- *Loxochoanella warburtoni* (56%)
- *Manchuroceras wolungense* (43%)
- *Moridunoceras castelli* (45%)
- *Najaceras triangulum* (63%)
- *Ordosoceras sphaeriforme* (42%)
- *Padunoceras rugosaeforme* (68%)
- *Polydesmia canaliculata* (42%)
- *Protothinoceras yini* (45%)
- *Pseudowutinoceras wuhaiense* (42%)
- *Qiushugouceras inclinatum* (56%)
- *Rummoceras rummuensis* (48%)
- *Striatocycloceras undulostriatum* (43%)
- *Tangshanoceras endogastrum* (45%)

- *Teichertoceras sinclairi* (40%)
- *Thylacoceras kimberleyense* (41%)
- *Ventroloboceras furcillatum* (51%)
- *Wadema taylori* (44%)
- *Xiaoshanoceras jini* (51%)
- *Yanheceras endogastrum* (53%)

El: Excluding 33 early representatives of Ellesmerocerida, Plectronocerida and Yanhecerida:

- *Aetheloxoceras suxianense* (Yanheceratidae, Yanhecerida)
- *Annoceras costatum* (Ellesmeroceratidae, Ellesmerocerida)
- *Balkoceras gracile* (Balkoceratidae, Plectronocerida)
- *Bassleroceras perseus* (Bassleroceratidae, Ellesmerocerida)
- *Caseoceras contractum* (Ellesmeroceratidae, Ellesmerocerida)
- *Chabactoceras planum* (Ellesmeroceratidae, Ellesmerocerida)
- *Clelandoceras* (?) *rarum* (Apocrinoceratidae?)
- *Dwightoceras dactyloides* (Bassleroceratidae, Ellesmerocerida)
- *Eburoceras jiagouense* (Ellesmeroceratidae, Ellesmerocerida)
- *Ectenolites primus* (Ellesmeroceratidae, Ellesmerocerida)
- *Ellesmeroceras bridge* (Ellesmeroceratidae, Ellesmerocerida)
- *Eorudolfoceras antiquum* (Ellesmeroceratidae, Ellesmerocerida)
- *Huaihecerina elegans* (Huaiheceratidae, Ellesmerocerida)
- *Lawrenceoceras confertissimum* (Bassleroceratidae, Ellesmerocerida)
- *Lebetoceras oepiki* (Ellesmeroceratidae, Ellesmerocerida)
- *Loxochoanella warburtoni* (Ellesmeroceratidae, Ellesmerocerida)
- *Novacaroceras endogastrum* (Novacaroceratidae, Ellesmerocerida)
- *Oelandoceras haelluddenense* (Ellesmeroceratidae, Ellesmerocerida)
- *Oneotoceras loculosum* (Ellesmeroceratidae, Ellesmerocerida)
- *Palaeoceras mutabile* (Plectronoceratidae, Plectronocerida)
- *Paradakeoceras minor* (Ellesmeroceratidae, Ellesmerocerida)
- *Phthanoncoceras oelandense* (Phthanoncoceratidae, Ellesmerocerida)
- *Physalactinoceras bullatum* (Plectronoceratidae, Plectronocerida)
- *Plectronoceras cambria* (Plectronoceratidae, Plectronocerida)
- *Qiushugouceras inclinatum* (Ellesmeroceratidae, Ellesmerocerida)
- *Robsonoceras robsonense* (Ellesmeroceratidae, Ellesmerocerida)
- *Rudolfoceras cornuoryx* (Rudolfoceratidae, Ellesmerocerida)
- *Shideleroceras sinuatum* (Shideleroceratidae, Ellesmerocerida)
- *Sinoeremoceras* sp. (Plectronoceratidae, Plectronocerida)
- *Vassaroceras henrietta* (Ellesmeroceratidae, Ellesmerocerida)
- *Ventroloboceras furcillatum* (Ellesmeroceratidae, Ellesmerocerida)
- *Xiaoshanoceras jini* (Xiaoshanoceratidae, Ellesmerocerida)
- *Yanheceras endogastrum* (Yanheceratidae, Yanhecerida)

Rd: Excluding 86 randomly selected taxa (50% of total taxa):

- *Actinoceras tenuifilum*
- *Adamsoceras oelandicum*
- *Apsidoceras montrealense*
- *Arionoceras lotskirkense*
- *Bactroceras sandbergeri*
- *Bassleroceras champlainense*
- *Bassleroceras perseus*
- *Bathmoceras holmi*
- *Bentoceras proteus*
- *Bisonoceras corniforme*
- *Botellusoceras torpense*
- *Buttsoceras novemexicanum*
- *Cameroceras turrisoides*
- *Caseoceras contractum*
- *Cassinoceras wortheni*
- *Centrocyrtoceras annulatum*
- *Centrocyrtocerina frizonensis*
- *Centrotarphyceras seelyi*
- *Clarkoceras newtonwinchelli*
- *Clelandoceras rarum*
- *Clitendoceras sylesi*
- *Clytoceras capax*
- *Cyclolituites lynnensis*
- *Cycloplectoceras miseri*
- *Cyclostomiceras cassinense*
- *Cyrtonybyoceras haesitans*
- *Dawsonocers fenestratum*
- *Dideroceras incognitum*
- *Discoceras vasalemmense*
- *Dwightoceras dactyloides*
- *Eburoceras jiagouense*
- *Ellesmeroceras bridgei*
- *Eorudolfoceras antiquum*
- *Eothinoceras maitlandi*
- *Exoclitendoceras rochdalense*
- *Falcilituites decheni*
- *Glenisteroceras obscurum*
- *Gonioceras anceps*
- *Hedstroemoceras haelluddenense*
- *Isorthoceras wahlenbergi*
- *Kundoceras evansi*
- *Lambeoceras lambii*
- *Langgunites mucronulatus*
- *Lawrenceoceras confertissimum*

- *Leonardoceras parvum*
- *Mysterioceras australe*
- *Najaceras triangulatum*
- *Neumatoceras borense*
- *Nilssonoceras latisiphonatum*
- *Novacaroceras endogastrum*
- *Orthoceras regulare*
- *Orthonybyoceras dyeri*
- *Pachendoceras huzzahense*
- *Padunoceras rugosaeforme*
- *Palaeoceras mutabile*
- *Palaeodawsonocerina senckenbergi*
- *Parvihebetoceras wahli*
- *Physalactinoceras bullatum*
- *Pictetoceras oliviae*
- *Plagiostomoceras fragile*
- *Plectoceras jason*
- *Plectronoceras cambria*
- *Polydesmia canaliculata*
- *Polymeres demetarum*
- *Probillingsites scandinavicum*
- *Proterocameroceras brainerdi*
- *Protothinoceras yini*
- *Rioceras escandei*
- *Rudolfoceras cornuoryx*
- *Ruedemannoceras boycii*
- *Sacerdosoceras promus*
- *Saloceras chinianense*
- *Schuchertoceras troedssoni*
- *Shumardoceras complanatum*
- *Strandoceras tyriense*
- *Tafadnatoceras tiouririnense*
- *Tajaroceras wardae*
- *Tangshanoceras endogastrum*
- *Thylacoceras kimberleyense*
- *Tragoceras falcatum*
- *Trocholites depressus*
- *Troedssonella endoceroides*
- *Ventroloboceras furcillatum*
- *Warburgoceras longitudinale*
- *Wutinoceras foerstei*
- *Xiaoshanoceras jini*

Text S4: Supplementary references

1. Teichert C, Kummel B, Sweet WC, Stenzel HB, Furnish WM, Glenister BF, et al. Treatise on Invertebrate Palaeontology, Part K, Mollusca 3, Cephalopoda. Moore RC, editor. Lawrence: Geological Society of America and University of Kansas Press; 1964.
2. Niko S, Mapes RH. Redescription and New Information on the Carboniferous Cephalopod Brachycycloceras normale Miller, Dunbar and Condra, 1933. Paleontol Res. 2009;13:337–343.
3. Flower RH. Nautiloid shell morphology. New Mex Bur Min Miner Ressources, Mem. 1964;13:1–79.
4. Flower RH. Development of the Mixochoanites. J Paleontol. 1941;15: 523–548.
5. Aubrechtová M. Review of ascocerid cephalopods from the upper Silurian of the Prague Basin (Central Bohemia) – history of research and palaeobiogeographic relationships. Foss Impr. 2019;75:14–24.
6. Brazeau MD. Problematic character coding methods in morphology and their effects. Biol J Linn Soc. 2011;104:489–498.
7. Kröger B. Nautiloids before and during the origin of ammonoids in a Siluro-Devonian section in the Tafilalt, anti-atlas, Morocco. Spec Pap Palaeontol. 2008;79: 1–110.
8. Kröger B. Revision of Middle Ordovician orthoceratacean nautiloids from Baltoscandia. Acta Palaeontol Pol. 2004;49:57–74.
9. Flower RH. Ordovician cephalopods of the Cincinnati region. Bull Am Paleontol. 1946;29:3–547.
10. Kröger B, Zhang Y-B, Isakar M. Discosorids and Oncocerids (Cephalopoda) of the Middle Ordovician Kunda and Aseri Regional Stages of Baltoscandia and the early evolution of these groups. Geobios. 2009;42:273–292.
11. Barskov IS. Conch ornamentation in nonammonoid cephalopods: form and function. Invertebr Zool. 2017;14:2–7.
12. Evans DH. The Lower and Middle Ordovician cephalopod faunas of England and Wales. Monogr Palaeontogr Soc. 2005;158:1–81.
13. Kröger B, Mapes RH. On the origin of bactritoids (Cephalopoda). Paläontol Zeitschr. 2007;81:316–327.
14. Aubrechtová M. A revision of the Ordovician cephalopod *Bactrites sandbergeri* Barrande: Systematic position and palaeobiogeography of *Bactroceras*. Geobios. 2015;48:193–211.
15. Seilacher A. Mechanische Simulation und funktionelle Evolution des Ammoniten-Septums [Mechanical simulation and functional evolution of the ammonite septum]. Paläontol Zeitschr. 1975;49:268–286.
16. Flower RH. The nautiloid order Ellesmeroceratida (Cephalopoda). New Mex Bur Min Miner Ressources, Mem. 1964;12:1–164.
17. Stridsberg S. Silurian oncocerid cephalopods from Gotland. Foss Strat. 1985;18: 1–65.
18. Turek V, Manda Š. “An endocochleate experiment” in the Silurian straight-shelled cephalopod *Sphooceras*. Bull Geosci. 2012;87:767–813.
19. Kröger B. The cephalopods of the Boda Limestone, Late Ordovician, of Dalarna, Sweden. Eur J Taxon. 2013;41: 1–110.
20. Foerste AF, Teichert C. The actinoceroids of east-central North America. Denison Univ Bull J Sci Lab. 1930;25:201–296.
21. Frye MW. Upper Ordovician (Harjuan) oncoceratid nautiloids from the Boda Limestone, Siljan District, Sweden. Geol Föreningen i Stock Förhandlingar. 1987;109:83–99.
22. Troedsson GT. Studies on Baltic fossil cephalopods. I. On the nautiloid genus

Orthoceras. Lund Univ Arsskrift, N F Avd 2. 1931;27:1–36.
23. Flower RH. Cameral deposits in Orthoconic Nautiloids. Geol Mag. 1955;92:89–103.
24. Fischer AG, Teichert C. Cameral deposits in cephalopod shells. Univ Kansas Paleontol Contrib. 1969;37:1–30.
25. Dzik J. Phylogeny of the Nautiloidea. Palaeontol Pol. 1984;45:1–320.
26. Blind W. Über Anlage und Funktion von Kammerablagerungen in Orthoceren-Gehäusen [On formation and function of cameral deposits in shells of orthocerids]. Palaeontogr Abteilung A. 1991;218:35–47.
27. Zhuravleva FA, Doguzhaeva LA. Astrovioidea: A new superorder of paleozoic cephalopods. Paleontol J. 2004;38:S1–S73.
28. Seuss B, Mapes RH, Klug C, Nützel A. Exceptional Cameral Deposits in a Sublethally Injured Carboniferous Orthoconic Nautiloid from the Buckhorn Asphalt Lagerstätte in Oklahoma, USA. Acta Palaeontol Pol. 2012;57:375–390.
29. Pohle A, Klug C. Early and Middle Devonian cephalopods from Hamar Laghdad (Tafilalt, Morocco) and remarks on epicoles and cameral deposits. Neues Jahrb für Geol und Paläontol - Abhandlungen. 2018;290:203–240.
30. King AH, Evans DH. High-level classification of the nautiloid cephalopods: a proposal for the revision of the Treatise Part K. Swiss J Palaeontol. 2019;138:65–85.
31. Mutvei H. A preliminary report on the structure of the siphonal tube and on the precipitation of lime in the shells of fossil Nautiloids. Ark för Mineral och Geol. 1956;2:179–191.
32. Mutvei H. Cameral deposits in Paleozoic cephalopods. GFF. 2018;140:254–263.
33. Mutvei H. Connecting ring structure and its significance for classification of the orthoceratid cephalopods. Acta Palaeontol Pol. 2002;47:157–168.
34. Sweet WC. The Middle Ordovician of the Oslo region of Norway. 10. Nautiloid cephalopods. Nor Geol Tidsskr. 1958;31:1–178.
35. Kröger B, Beresi MS, Landing E. Early orthoceratoid cephalopods from the Argentine Precordillera (Lower-Middle Ordovician). J Paleontol. 2007;81:1266–1283.
36. Chen J-Y, Teichert C. Cambrian Cephalopoda of China. Palaeontogr Abteilung A. 1983;181:1–102.
37. Wade M. Nautiloids and their descendants: cephalopod classification in 1986. New Mex Bur Min Miner Ressources, Mem. 1988;44:15–25.
38. Wade M, Stait B. Subclass Nautiloidea - Introduction and fossil record. In: Beesley PL, Ross GJB, Wells A, editors. Mollusca: The Southern Synthesis, Part A, Fauna of Australia Volume 5. Canberra: Australian Biological Resources Study; 1993. pp. 485–493.
39. Mutvei H, Zhang Y-B, Dunca E. Late Cambrian plectronocerid nautiloids and their role in cephalopod evolution. Palaeontology. 2007;50:1327–1333.
40. Mutvei H. Restudy of some plectronocerid nautiloids (Cephalopoda) from the late Cambrian of China; discussion on nautiloid evolution and origin of the siphuncle. GFF. 2020;142:115–124.
41. Flower RH. Cambrian cephalopods. New Mex Bur Min Miner Ressources, Bull. 1954;40:1–51.
42. Mutvei H. Silurian oncocerid *Octamerella* (Cephalopoda) from Gotland, Sweden. GFF. 2011;133:125–133.
43. Mutvei H, Dunca E. Siphuncular structure in the orders Tarphycerida and Barrandeocerida (Cephalopoda: Nautiloidea). Palaeontology. 2011;54:705–710.
44. Mutvei H. Characterization of nautiloid orders Ellesmerocerida, Oncocerida, Tarphycerida, Discosorida and Ascocerida: new superorder Multiceratoidea. GFF. 2013;135:171–183.

45. Mutvei H. Siphuncular structure in Silurian discosorid and ascocerid nautiloids (Cephalopoda) from Gotland, Sweden: implications for interpretation of mode of life and phylogeny. GFF. 2012;134:27–37.
46. Mutvei H. Siphuncular structures in Calciosiphonate nautiloid orders Actinocerida, Orthocerida and Barrandeocerida (Cephalopoda). GFF. 2016;138:295–305.
47. Mutvei H. Characterization of two new superorders Nautilosiphonata and Calciosiphonata and a new order Cyrtocerinida of the subclass Nautiloidea; siphuncular structure in the Ordovician nautiloid Bathmoceras (Cephalopoda). GFF. 2015;137:164–174.
48. Mutvei H. The new order Mixosiphonata (Cephalopoda: Nautiloidea) and related taxa; estimations of habitat depth based on shell structure. GFF. 2017;139:219–232.
49. Flower RH, Teichert C. The cephalopod order Discosorida. Univ Kansas, Paleontol Contrib. 1957;6:1–144.
50. Stait B. Ordovician nautiloids of Tasmania - Gouldoceratidae fam. nov. (Discosorida). Proc R Soc Victoria. 1984;96:187–207.
51. Evans DH, King AH. Resolving polyphyly within the Endocerida: The Bisonocerida nov., a new order of early palaeozoic nautiloids. Geobios. 2012;45:19–28.
52. Hook SC, Flower RH. *Tajaroceras* and the origin of the Troedssonellidae. J Paleontol. 1976;50:293–300.
53. Collins DH. Endocone diaphragms and the “phragmocone of *Ecdyceras*” (Nautiloidea). J Paleontol. 1967;41:1101–1112.
54. Kröger B. The “Vaginaten”: The dominant cephalopods of the Baltoscandian Mid Ordovician endocerid limestone. GFF. 2012;134:115–132.
55. Mutvei H. On the secondary internal calcerous lining of the wall of the siphonal tube in certain fossil “nautiloid” cephalopods. Ark för Zool Ser 2. 1964;16:375–424.
56. Balashov ZG. Novye ordovikskie nautiloidei SSSR [New Ordovician nautiloids of the USSR]. Novye Vidy Drevnikh Rastennyi i Besposvonochnykh SSSR. 1960;2:123–137.
57. Kröger B, Gutiérrez-Marco JC. First record of a nonpaleotropical intejocerid cephalopod from Darriwilian (Middle Ordovician) strata of central Spain. J Paleontol. 2020;94:273–278.
58. Teichert C. Der Bau der Actinoceroiden Cephalopoden [The structure of the actinoceroid cephalopods]. Palaeontogr Abteilung A. 1933;78:111–230.
59. Flower RH. Study of the Pseudorthoceratidae. Palaeontogr Am. 1939;2:245–461.
60. Wade M. Georginidae, New Family of Actinoceratoid Cephalopods, Middle Ordovician, Australia. Mem Queensl Museum. 1977;18:1–15.
61. Wade M. The siphuncle in Georginidae and other Ordovician actinoceroid cephalopods. Lethaia. 1977;10:303–315.
62. Ebbestad JOR, Polechová M, Kröger B, Gutiérrez-Marco JC. Late Ordovician molluscs of the central and eastern Anti-Atlas, Morocco. Geol Soc London, Spec Publ. 2019;485. doi:10.1144/SP485.9
63. Flower RH. Studies of the Actinoceratida. New Mex Bur Min Miner Ressources, Mem. 1957;2:1–73.
64. Crick RE, Teichert C. Siphuncular structures in the Devonian nautiloid *Archiacoceras* from the Eifel of West Germany. Palaeontology. 1979;22:747–766.
65. Flower RH. Studies on Paleozoic Nautiloidea IV: Investigations of actinosiphonate cephalopods. Bull Am Paleontol. 1943;28:30–67.
66. Chen J-Y, Teichert C. The Ordovician cephalopod suborder Cyrtocerinina (order Ellesmerocerida). Palaeontol Cathayana. 1987;3:145–229.
67. Cichowolski M, Waisfeld BG, Vaccari NE, Marengo L. The nautiloid Family Eothinoceratidae from the Floian of the Central Andean Basin (NW Argentina and

South Bolivia). Geol J. 2015;50:764–782.
68. Mutvei H. On the relations of the principal muscles to the shell in Nautilus and some fossil nautiloids. Ark för Mineral och Geol. 1957;2:219–254.
69. Sweet WC. Muscle attachment impressions of some Paleozoic nautiloid cephalopods. J Paleontol. 1959;33:55–62.
70. Pohle A, Klug C, Toom U, Kröger B. Conch structures, soft-tissue imprints and taphonomy of the Middle Ordovician cephalopod *Tragoceras falcatum* from Estonia. Foss Impr. 2019;75:70–78.
71. Kröger B, Mutvei H. Nautiloids with multiple paired muscle scars from lower-middle Ordovician of Baltoscandia. Palaeontology. 2005;48:781–791.
72. Kröger B, Isakar M. Revision of annulated orthoceridan cephalopods of the Baltoscandic Ordovician. Foss Rec. 2006;9:137–163.
73. Engeser T. The Position of the Ammonoidea within the Cephalopoda. In: Landman NH, Tanabe K, Davis RA, editors. Ammonoid Paleobiology Topics in Geobiology 13. New York: Plenum Press; 1996. pp. 3–19.
74. Kröger B. Early growth-stages and classification of orthoceridan cephalopods of the Darriwillian (Middle Ordovician) of Baltoscandia. Lethaia. 2006;39:129–139.
75. Kröger B, Mapes RH. Carboniferous Actinoceratoid Nautiloidea (Cephalopoda)— a New Perspective. J Paleontol. 2007;81:714–724.
76. Manda Š, Turek V. Embryonic and early juvenile development in the basal Silurian nautilid Peismoceras Hyatt, 1894. Swiss J Palaeontol. 2019;138:123-139.
77. Arnold JM. Reproduction and embryology of *Nautilus*. In: Sanders WB, Landman NH, editors. *Nautilus*: The Biology and Paleobiology of a Living Fossil. New York: Plenum Press; 1987. pp. 353–372.
78. Kröger B, Mapes RH. Lower Carboniferous (Chesterian) embryonic orthoceratid nautiloids. J Paleontol. 2004;78:560–573.
79. Evans DH. The Lower Ordovician cephalopod faunas of the Durness group, North-West Scotland. Monogr Palaeontogr Soc. 2011;165:1–131.
80. Kobayashi T. On the phylogeny of the primitive nautiloids, with descriptions of *Plectronoceras liaotungense*, new species and *Iddingsia* (?) *shantungensis*, new species. Japanese J Geol Geogr. 1935;12:17–26.
81. Teichert C, Kummel B. Size of endoceroid cephalopods. Breviora, Museum Comp Zool. 1960;128:1–7.
82. Ulrich EO, Foerste AF, Miller AK, Furnish WM. Ozarkian and Canadian cephalopods part I: nautilicones. Geol Soc Am Spec Pap. 1942;37:1–157.
83. King AH. Taxonomic review of early Darriwilian estonioceratids (Tarphycerida, Nautiloidea) from Sweden, Estonia, and the 'Diluvium-Geschiebe' of northern Germany and Poland. Kataloge des Oberösterreichischen Landesmuseums, Neue Ser. 2014;157:47–57.
84. Pohle A, Klug C. Body size of orthoconic cephalopods from the late Silurian and Devonian of the Anti-Atlas (Morocco). Lethaia. 2018;51:126–148.
85. Chen J-Y, Tsou S-P, Chen T-E, Qi D-L. Late Cambrian cephalopods of North China. Plectronocerida, Protactinocerida (ord. nov.) and Yanhecerida (ord. nov.). Acta Palaeontol Sin. 1979;18:1–24.
86. Chen J-Y, Zou X-P, Chen T-E, Qi D-L. Late Cambrian Ellesmerocerida (Cephalopoda) of North China. Acta Palaeontol Sin. 1979;18:103–124.
87. Chen J-Y, Zou X-P. Ordovician cephalopods from the Ordos area, China. Acta Palaeontol Sin. 1984;20:33–84.
88. Korn D, Klug C. Conch Form Analysis, Variability, Morphological Disparity, and Mode of Life of the Frasnian (Late Devonian) Ammonoid *Manticoceras* from Coumiac

(Montagne Noire, France). In: Landman NH, Davis RA, Mapes RH, editors. Cephalopods Present and Past: New Insights and Fresh Perspectives. Dordrecht: Springer Netherlands; 2007. pp. 57–85.
89. Kröger B, Aubrechtová M. Cephalopods from reef limestone of the Vasalemma Formation, northern Estonia (latest Sandbian, Upper Ordovician) and the establishment of a local warm-water fauna. J Syst Palaeontol. 2018;16:799–839.
90. Aronoff SM. Orthoconic nautiloid morphology and the case of *Treptoceras* vs. *Orthonybyoceras*. Neues Jahrb für Geol und Paläontologie - Abhandlungen. 1979;158:100–122.
91. Frey RC. Middle and Upper Ordovician Nautiloid Cephalopods of the Cincinnati Arch Region of Kentucky, Indiana, and Ohio. Contrib to Ordovician Paleontol Kentucky nearby states US Geol Surv Prof Pap. 1995;1066:P1–P126.
92. Fang X, Zhang Y-B, Chen T-E, Zhang Y-D. A quantitative study of the Ordovician cephalopod species *Sinoceras chinense* (Foord) and its palaeobiogeographic implications. Alcheringa. 2017;41:321–334.
93. Kröger B, Aubrechtová M. The cephalopods of the Kullsberg Limestone Formation, Upper Ordovician, central Sweden and the effects of reef diversification on cephalopod diversity. J Syst Palaeontol. 2019;17:961-995.
94. Flower RH, Kummel B. A Classification of the Nautiloidea. J Paleontol. 1950;24:604–616.
95. Kröger B. The size of the siphuncle in cephalopod evolution. Senckenbergiana lethaea. 2003;83:39–52.
96. Holm G. Palaeontologiska notiser. Om *Bathmoceras* [Palaeontological notes. On *Bathmoceras*]. Geol Föreningen i Stock Förhandlingar. 1899;21:271–304.

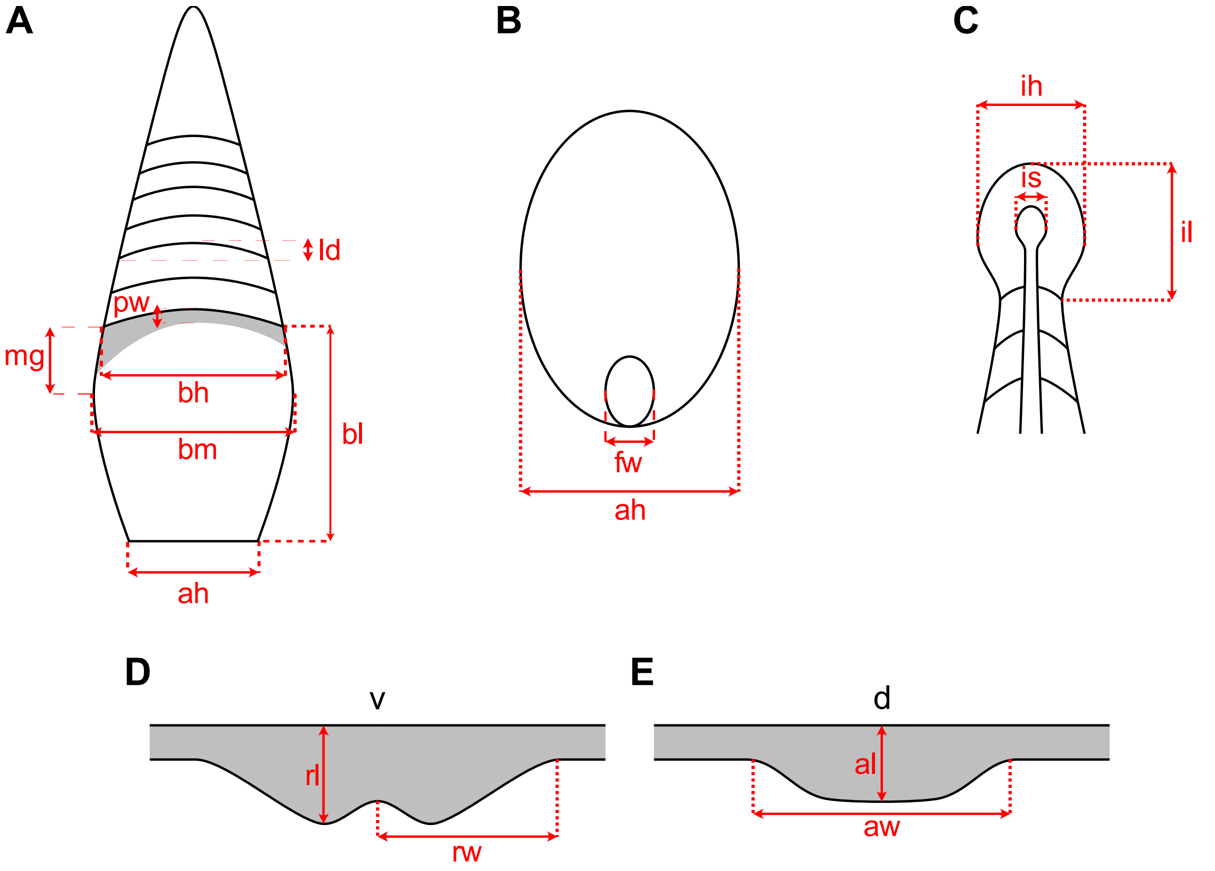

Fig. S1. Measurements.

Basic measurements that were used to calculate conch parameters. For abbreviations, see Additional file 1: Table S1. (**A**): Internal mold in lateral view. Grey area represents periphract and lines on the phragmocone represent suture lines. Orientation with aperture downwards. (**B**): Cross section of phragmocone at the position of the septal foramen. Inner circle represents siphuncle. (**C**): Earliest ontogenetic stages of the conch in median section, showing initial chamber and caecum. Orientation with aperture downwards. (**D**): Ventral periphract including retractor muscles. Orientation with aperture downwards. (**E**): Dorsal periphract including dorsal aponeurosis. Orientation with aperture downwards. Further measurements are shown in Fig. 1A of the main text.

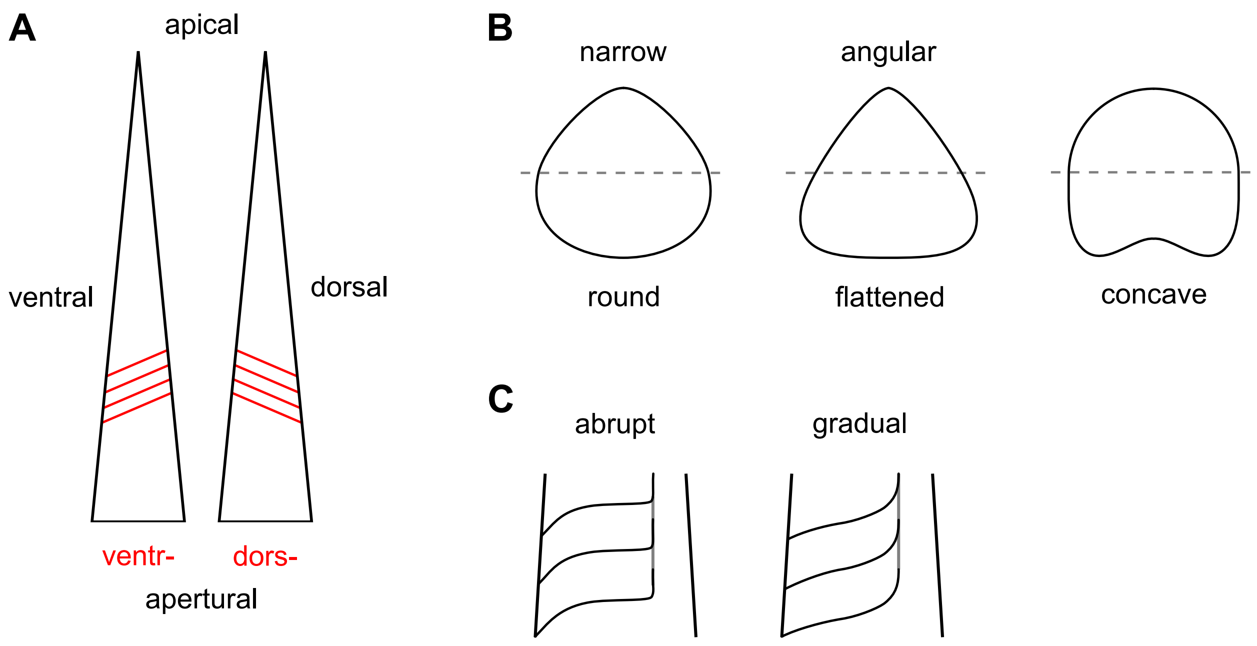

Fig. S2. Illustrations of characters.

Except for cross sections, all illustrations are oriented with aperture downwards. (**A**): Terminology of growth directions (ventrapertural and dorsapertural) of growth lines (character 3), annulations (character 8.1), sutures (character 14), and diaphragms (character 34.3). Straight lines are termed transverse. (**B**): Conch cross section shape, characters 10.-12. (**C**): Septal neck transition, character 26.

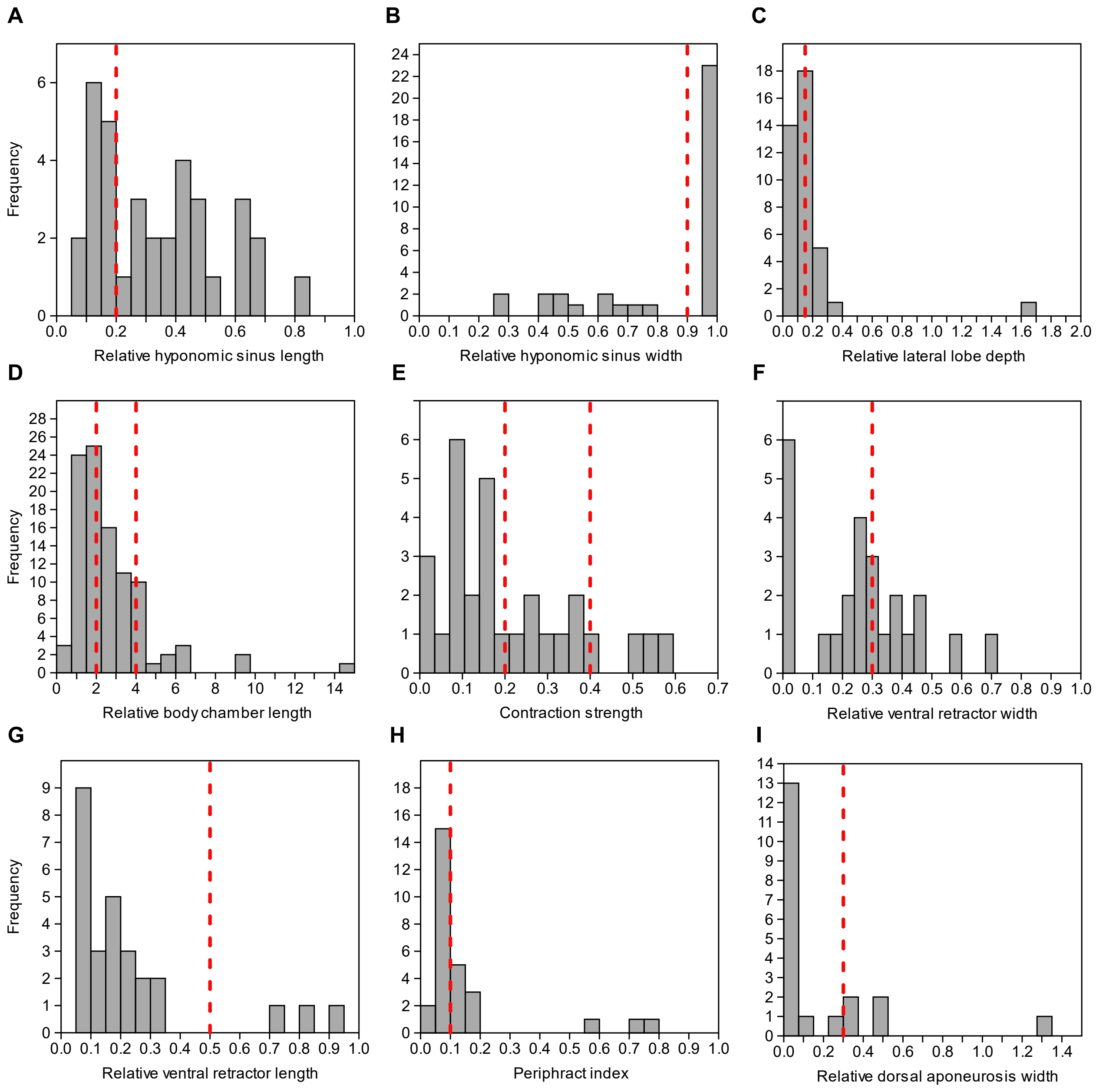

Fig. S3. Distribution of conch parameters (1/3).

Histograms of continuous, discretized characters. Red dotted lines represent threshold values between states. (**A**): Relative hyponomic sinus length, character 5.1. (**B**): Relative hyponomic sinus width, character 5.2. (**C**): Relative lateral lobe depth, character 13.3.2. (**D**): Relative body chamber length, character 18. (**E**): Contraction strength, character 20.2. (**F**): Relative ventral retractor width, character 42.1. (**G**): Relative ventral retractor length, character 42.2. (**H**): Periphract index, character 43. (**I**): Relative dorsal aponeurosis width, character 44.1.

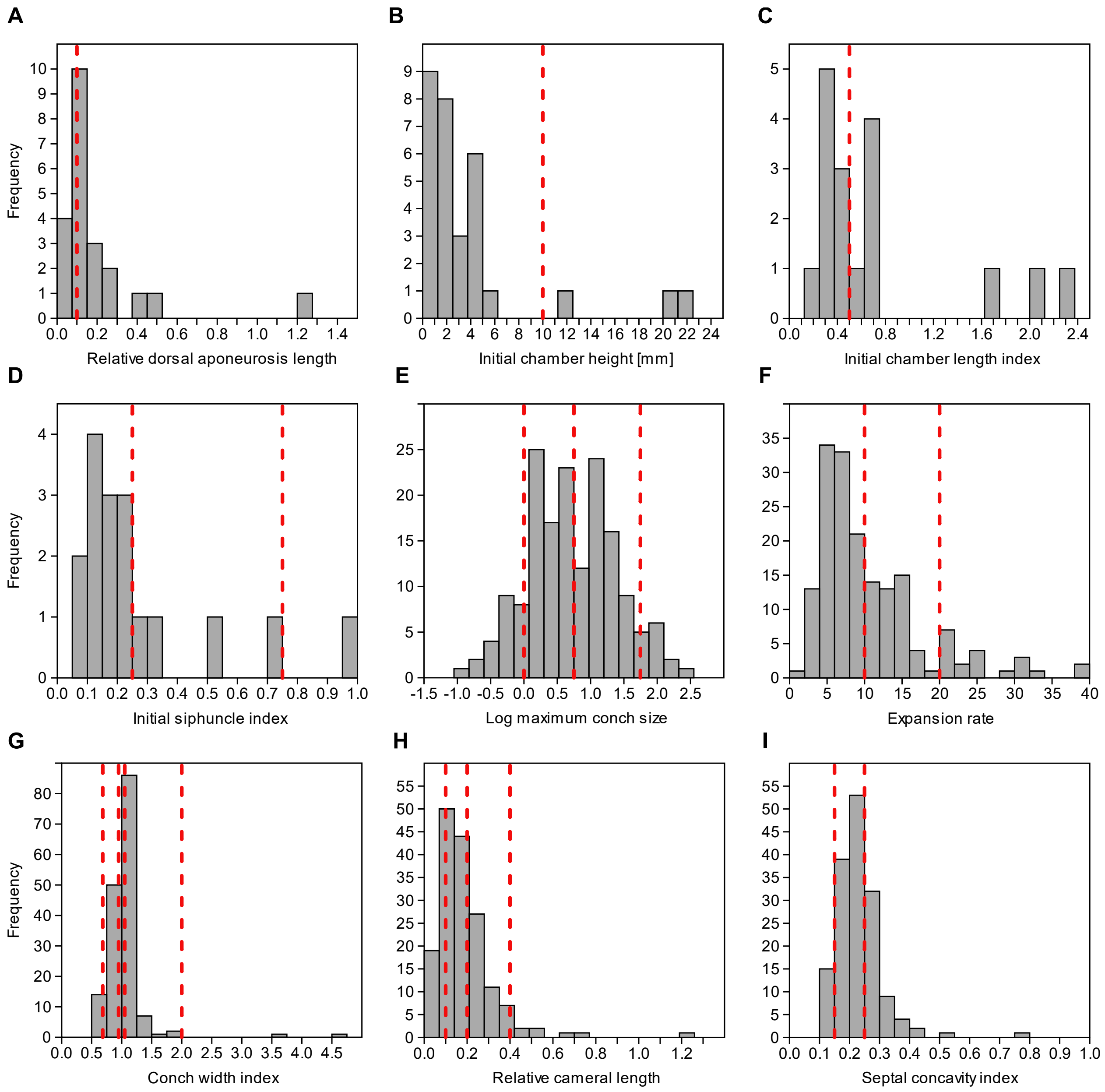

Fig. S4. Distribution of conch parameters (2/3).

Histograms of continuous, discretized characters. Red dotted lines represent threshold values between states. (**A**): Relative dorsal aponeurosis length, character 44.2. (**B**): Initial chamber height, character 48. (**C**): Initial chamber length index, character 49. (**D**): Initial siphuncle index, character 51.1. (**E**): Maximum conch size, character 60. (**F**): Expansion rate, character 63. (**G**): Conch width index, character 64. (**H**): Relative cameral length, character 65. (**I**): Septal concavity index, character 66.

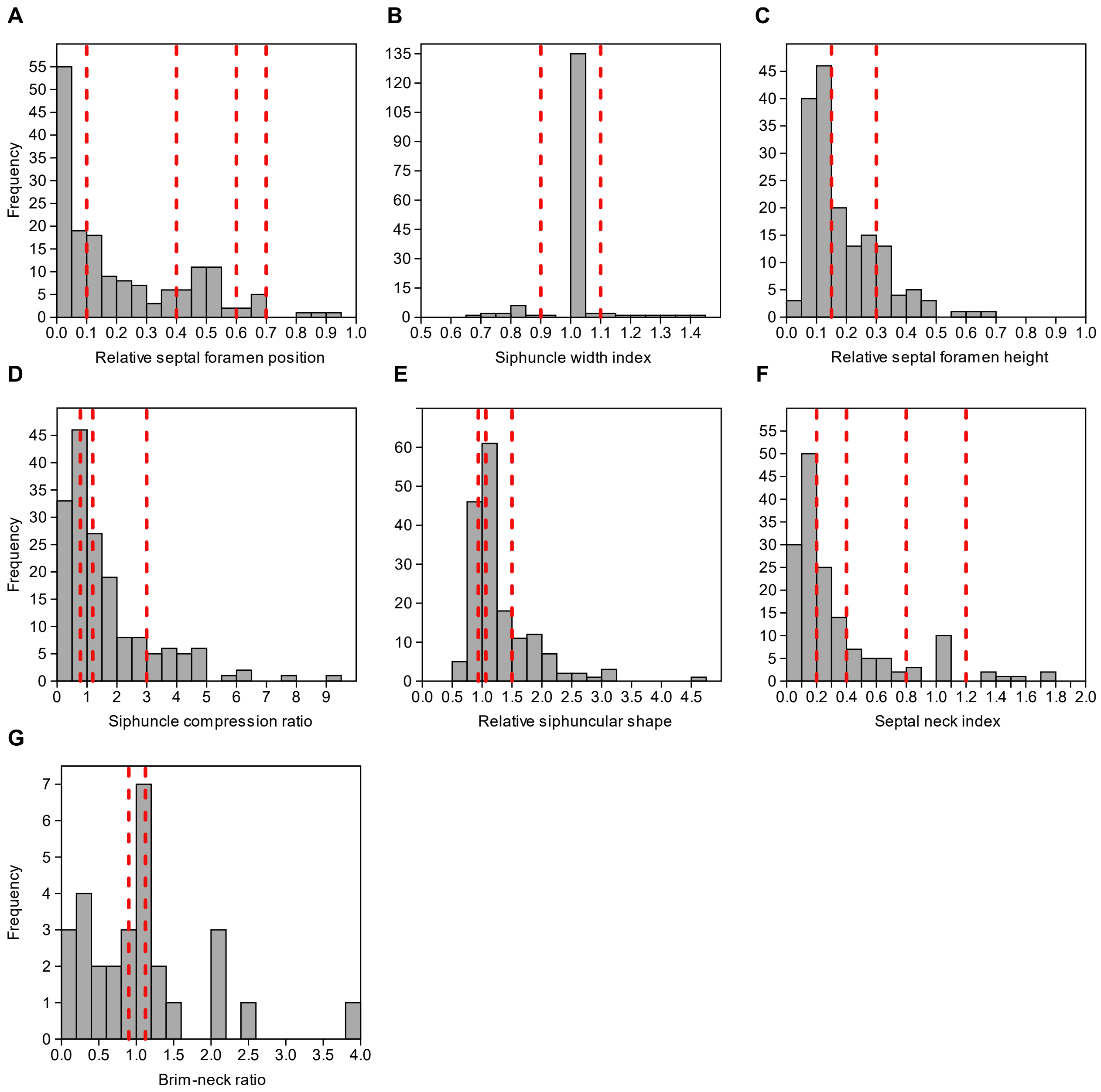

Fig. S5. Distribution of conch parameters (3/3).

Histograms of continuous, discretized characters. Red dotted lines represent threshold values between states. (**A**): Relative septal foramen position, character 67. (**B**): Siphuncle width index, character 68. (**C**): Relative septal foramen height, character 67. (**D**): Siphuncle compression ratio, character 70. (**E**): Relative siphuncular shape, character 71. (**F**): Septal neck index, character 72. (**G**): Brim-neck ratio, character 72.1.2.

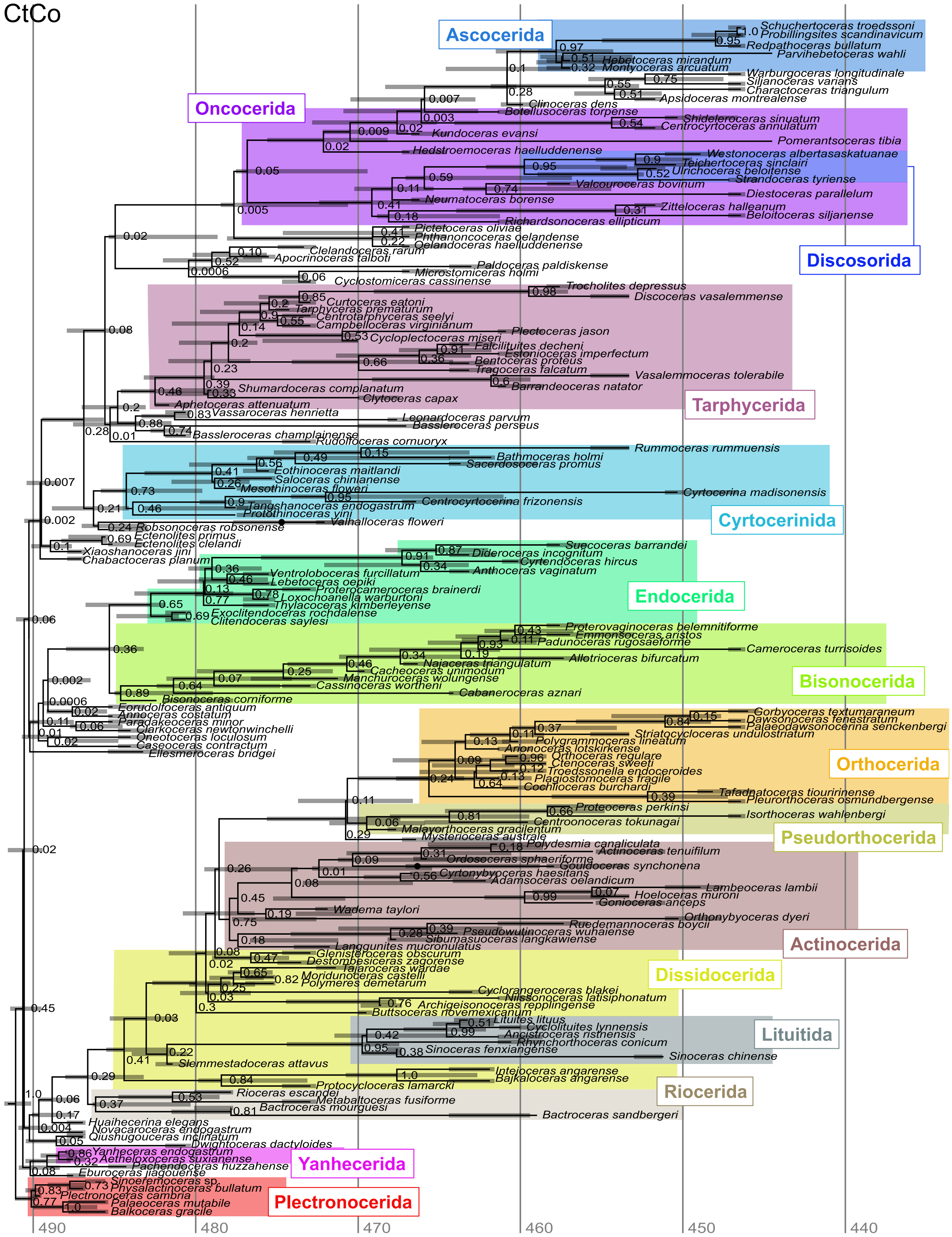
CtCo
Ascocerida
Oncocerida
Discosorida
Tarphycerida
Cyrtocerinida
Endocerida
Bisonocerida
Orthocerida
Pseudorthocerida
Actinocerida
Dissidocerida
Lituitida
Riocerida
Yanhecerida
Plectronocerida
Schuchertoceras troedssoni
Probillingsites scandinavicum
Redpathoceras bullatum
Parvihebetoceras wahli
Hebetoceras mirandum
Montyoceras arcuatum
Warburgoceras longitudinale
Siljanoceras varians
Charactoceras triangulum
Apsidoceras montrealense
Clinoceras dens
Botellusoceras torpense
Shideleroceras sinuatum
Centrocyrtoceras annulatum
Kundoceras evansi
Pomerantsoceras tibia
Hedstroemoceras haelluddenense
Westonoceras albertasaskatuanae
Teichertoceras sinclairi
Ulrichoceras beloitense
Strandoceras tyriense
Valcouroceras bovinum
Diestoceras parallelum
Neumatoceras borense
Zitteloceras halleanum
Beloitoceras siljanense
Richardsonoceras ellipticum
Pictetoceras oliviae
Phthanoncoceras oelandense
Oelandoceras haelluddenense
Clelandoceras rarum
Apocrinoceras talboti
Paldoceras paldiskense
Microstomiceras holmi
Cyclostomiceras cassinense
Trocholites depressus
Discoceras vasalemmense
Curtoceras eatoni
Tarphyceras prematurum
Centrotarphyceras seelyi
Campbelloceras virginianum
Plectoceras jason
Cycloplectoceras miseri
Falcilituites decheni
Estonioceras imperfectum
Bentoceras proteus
Tragoceras falcatum
Vasalemmoceras tolerabile
Barrandeoceras natator
Shumardoceras complanatum
Clytoceras capax
Aphetoceras attenuatum
Vassaroceras henrietta
Leonardoceras parvum
Bassleroceras perseus
Bassleroceras champlainense
Rudolfoceras cornuoryx
Rummoceras rummuensis
Bathmoceras holmi
Sacerdosoceras promus
Eothinoceras maitlandi
Saloceras chinianense
Mesothinoceras floweri
Cyrtocerina madisonensis
Centrocyrtocerina frizonensis
Tangshanoceras endogastrum
Protothinoceras yini
Valhalloceras floweri
Robsonoceras robsonense
Ectenolites primus
Ectenolites clelandi
Xiaoshanoceras jini
Chabactoceras planum
Suecoceras barrandei
Dideroceras incognitum
Cyrtendoceras hircus
Anthoceras vaginatum
Ventroloboceras furcillatum
Lebetoceras oepiki
Proterocameroceras brainerdi
Loxochoanella warburtoni
Thylacoceras kimberleyense
Exoclitendoceras rochdalense
Clitendoceras saylesi
Proterovaginoceras belemnitiforme
Emmonsoceras aristos
Padunoceras rugosaeforme
Cameroceras turrisoides
Allotrioceras bifurcatum
Najaceras triangulatum
Cacheoceras unimodum
Manchuroceras wolungense
Cassinoceras wortheni
Cabaneroceras aznari
Bisonoceras corniforme
Eorudolfoceras antiquum
Annoceras costatum
Paradakeoceras minor
Clarkoceras newtonwinchelli
Oneotoceras loculosum
Caseoceras contractum
Ellesmeroceras bridgei
Gorbyoceras textumaraneum
Dawsonoceras fenestratum
Palaeodawsonocerina senckenbergi
Striatocycloceras undulostriatum
Polygrammoceras lineatum
Arionoceras lotskirkense
Orthoceras regulare
Ctenoceras sweeti
Troedssonella endoceroides
Plagiostomoceras fragile
Cochlioceras burchardi
Tafadnatoceras tiouririnense
Pleurorthoceras osmundbergense
Proteoceras perkinsi
Isorthoceras wahlenbergi
Centroonoceras tokunagai
Malayorthoceras gradilentum
Mysterioceras australe
Polydesmia canaliculata
Actinoceras tenuifilum
Ordosoceras sphaeriforme
Gouldoceras synchonena
Cyrtonybyoceras haesitans
Adamsoceras oelandicum
Lambeoceras lambii
Hoeloceras muroni
Gonioceras anceps
Wadema taylori
Orthonybyoceras dyeri
Ruedemannoceras boycii
Pseudowutinoceras wuhaiense
Sibumasuoceras langkawiense
Langgunites mucronulatus
Glenisteroceras obscurum
Destombesiceras zagorense
Tajaroceras wardae
Moridunoceras castelli
Polymeres demetarum
Cyclorangeroceras blakei
Nilssonoceras latisiphonatum
Archigeisonoceras repplingense
Buttsoceras novemexicanum
Lituites lituus
Cyclolituites lynnensis
Ancistroceras risthensis
Rhynchorthoceras conicum
Sinoceras fenxiangense
Sinoceras chinense
Slemmestadoceras attavus
Intejoceras angarense
Bajkaloceras angarense
Protocycloceras lamarcki
Rioceras escandei
Metabaltoceras fusiforme
Bactroceras mourguesi
Bactroceras sandbergeri
Huaihecerina elegans
Novacaroceras endogastrum
Qiushugouceras inclinatum
Dwightoceras dactyloides
Yanheceras endogastrum
Aetheloxoceras suxianense
Pachendoceras huzzahense
Eburoceras jiagouense
Sinoeremoceras sp.
Physalactinoceras bullatum
Plectronoceras cambria
Palaeoceras mutabile
Balkoceras gracile
490
480
470
460
450
440

Fig. S6. Full MCC tree of the CtCo analysis.

This analysis excluded all controversial characters. Numbers at nodes represent posterior probabilities. Colored boxes represent orders. Some species were recovered as sampled ancestors, represented here by black dots.

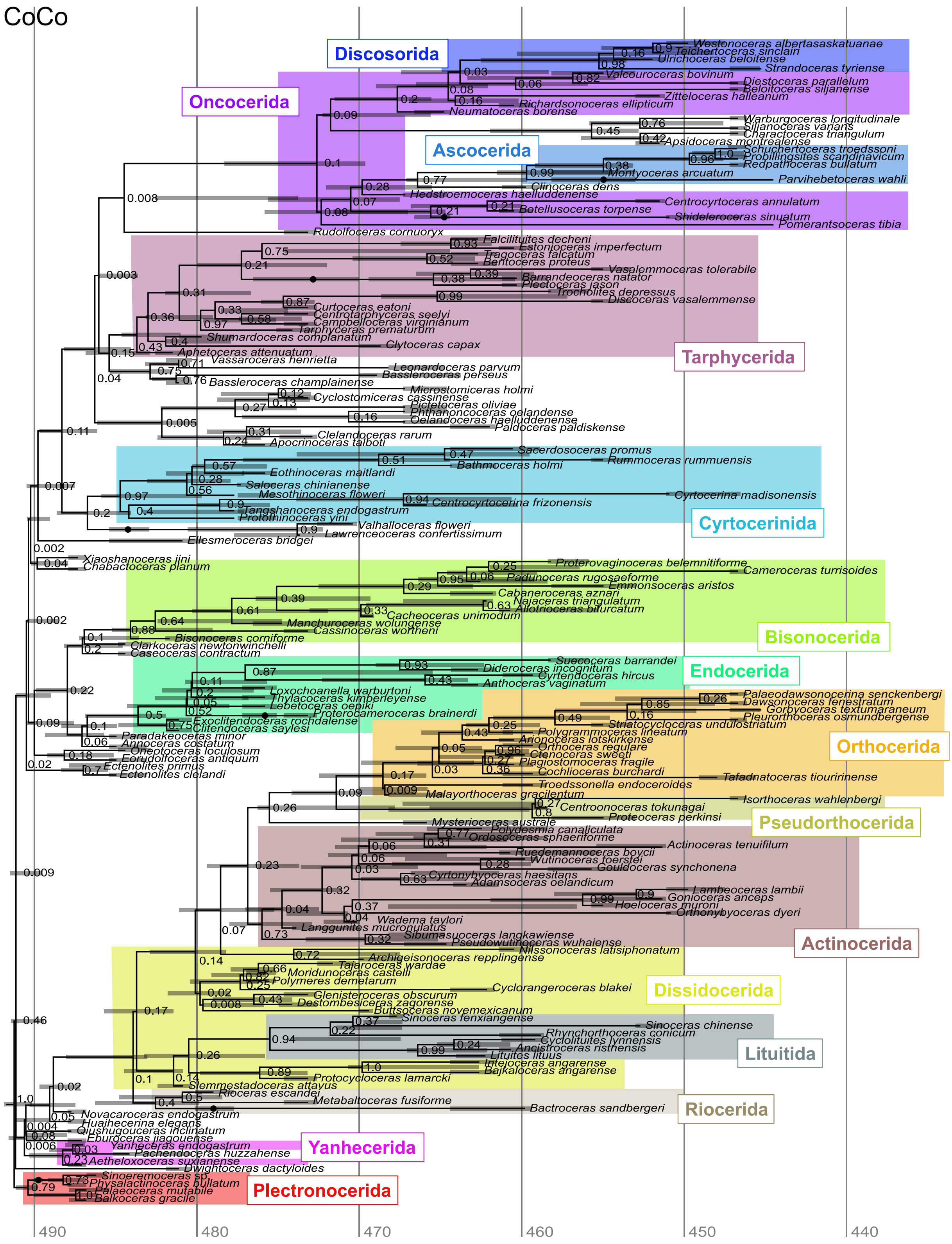
CoCo
Discosorida
Oncocerida
Ascocerida
Tarphycerida
Cyrtocerinida
Bisonocerida
Endocerida
Orthocerida
Pseudorthocerida
Actinocerida
Dissidocerida
Lituitida
Riocerida
Yanhecerida
Plectronocerida
Westonoceras albertasaskatuanae
Teichertoceras sinclairi
Ulrichoceras beloitense
Strandoceras tyriense
Valcouroceras bovinum
Diestoceras parallelum
Beloitoceras siljanense
Zitteloceras halleanum
Richardsonoceras ellipticum
Neumatoceras borense
Warburgoceras longitudinale
Siljanoceras varians
Charactoceras triangulum
Apsidoceras montrealense
Schuchertoceras troedssoni
Probillingsites scandinavicum
Redpathoceras bullatum
Montyoceras arcuatum
Parvihebetoceras wahli
Clinoceras dens
Hedstroemoceras haelluddenense
Centrocyrtoceras annulatum
Botellusoceras torpense
Shideleroceras sinuatum
Pomerantsoceras tibia
Rudolfoceras cornuoryx
Falcilituites decheni
Estonioceras imperfectum
Tragoceras falcatum
Bentoceras proteus
Vasalemmoceras tolerabile
Barrandeoceras natator
Plectoceras jason
Trocholites depressus
Discoceras vasalemmense
Curtoceras eatoni
Centrotarphyceras seelyi
Campbelloceras virginianum
Tarphyceras prematurum
Shumardoceras complanatum
Clytoceras capax
Aphetoceras attenuatum
Vassaroceras henrietta
Leonardoceras parvum
Bassleroceras perseus
Bassleroceras champlainense
Microstomiceras holmi
Cyclostomiceras cassinense
Pictetoceras oliviae
Phthanoncoceras oelandense
Oelandoceras haelluddenense
Paldoceras paldiskense
Clelandoceras rarum
Apocrinoceras talboti
Sacerdosoceras promus
Rummoceras rummuensis
Bathmoceras holmi
Eothinoceras maitlandi
Saloceras chinianense
Mesothinoceras floweri
Cyrtocerina madisonensis
Centrocyrtocerina frizonensis
Tangshanoceras endogastrum
Protothinoceras yini
Valhalloceras floweri
Lawrenceoceras confertissimum
Ellesmeroceras bridgei
Xiaoshanoceras jini
Chabactoceras planum
Proterovaginoceras belemnitiforme
Cameroceras turrisoides
Padunoceras rugosaeforme
Emmonsoceras aristos
Cabaneroceras aznari
Najaceras triangulatum
Allotrioceras bifurcatum
Cacheoceras unimodum
Manchuroceras wolungense
Cassinoceras wortheni
Bisonoceras corniforme
Clarkoceras newtonwinchelli
Caseoceras contractum
Suecoceras barrandei
Dideroceras incognitum
Cyrtendoceras hircus
Anthoceras vaginatum
Loxochoanella warburtoni
Thylacoceras kimberleyense
Lebetoceras oepiki
Proterocameroceras brainerdi
Exoclitendoceras rochdalense
Clitendoceras saylesi
Paradakeoceras minor
Annoceras costatum
Oneotoceras loculosum
Eorudolfoceras antiquum
Ectenolites primus
Ectenolites clelandi
Palaeodawsonocerina senckenbergi
Dawsonoceras fenestratum
Gorbyoceras textumaraneum
Pleurorthoceras osmundbergense
Striatocycloceras undulostriatum
Polygrammoceras lineatum
Arionoceras lotskirkense
Orthoceras regulare
Ctenoceras sweeti
Plagiostomoceras fragile
Cochlioceras burchardi
Tafadnatoceras tiouririnense
Troedssonella endoceroides
Malayorthoceras gracilentum
Isorthoceras wahlenbergi
Centroonoceras tokunagai
Proteoceras perkinsi
Mysterioceras australe
Polydesmia canaliculata
Ordosoceras sphaeriforme
Actinoceras tenuifilum
Ruedemannoceras boycii
Wutinoceras foerstei
Gouldoceras synchonena
Cyrtonybyoceras haesitans
Adamsoceras oelandicum
Lambeoceras lambii
Gonioceras anceps
Hoeloceras muroni
Orthonybyoceras dyeri
Wadema taylori
Langgunites mucronulatus
Sibumasuoceras langkawiense
Pseudowutinoceras wuhaiense
Nilssonoceras latisiphonatum
Archigeisonoceras repplingense
Tajaroceras wardae
Moridunoceras castelli
Polymeres demetarum
Cyclorangeroceras blakei
Glenisteroceras obscurum
Destombesiceras zagorense
Buttsoceras novemexicanum
Sinoceras fenxiangense
Sinoceras chinense
Rhynchorthoceras conicum
Cyclolituites lynnensis
Ancistroceras risthensis
Lituites lituus
Intejoceras angarense
Bajkaloceras angarense
Protocycloceras lamarcki
Slemmestadoceras attavus
Rioceras escandei
Metabaltoceras fusiforme
Bactroceras sandbergeri
Novacaroceras endogastrum
Huaihecerina elegans
Qiushugouceras inclinatum
Eburoceras jiagouense
Yanheceras endogastrum
Pachendoceras huzzahense
Aetheloxoceras suxianense
Dwightoceras dactyloides
Sinoeremoceras sp.
Physalactinoceras bullatum
Palaeoceras mutabile
Balkoceras gracile
490
480
470
460
450
440

Fig. S7. Full MCC tree of the CoCo analysis.

This analysis explicitly included controversial characters. Numbers at nodes represent posterior probabilities. Colored boxes represent orders. Some species were recovered as sampled ancestors, represented here by black dots.

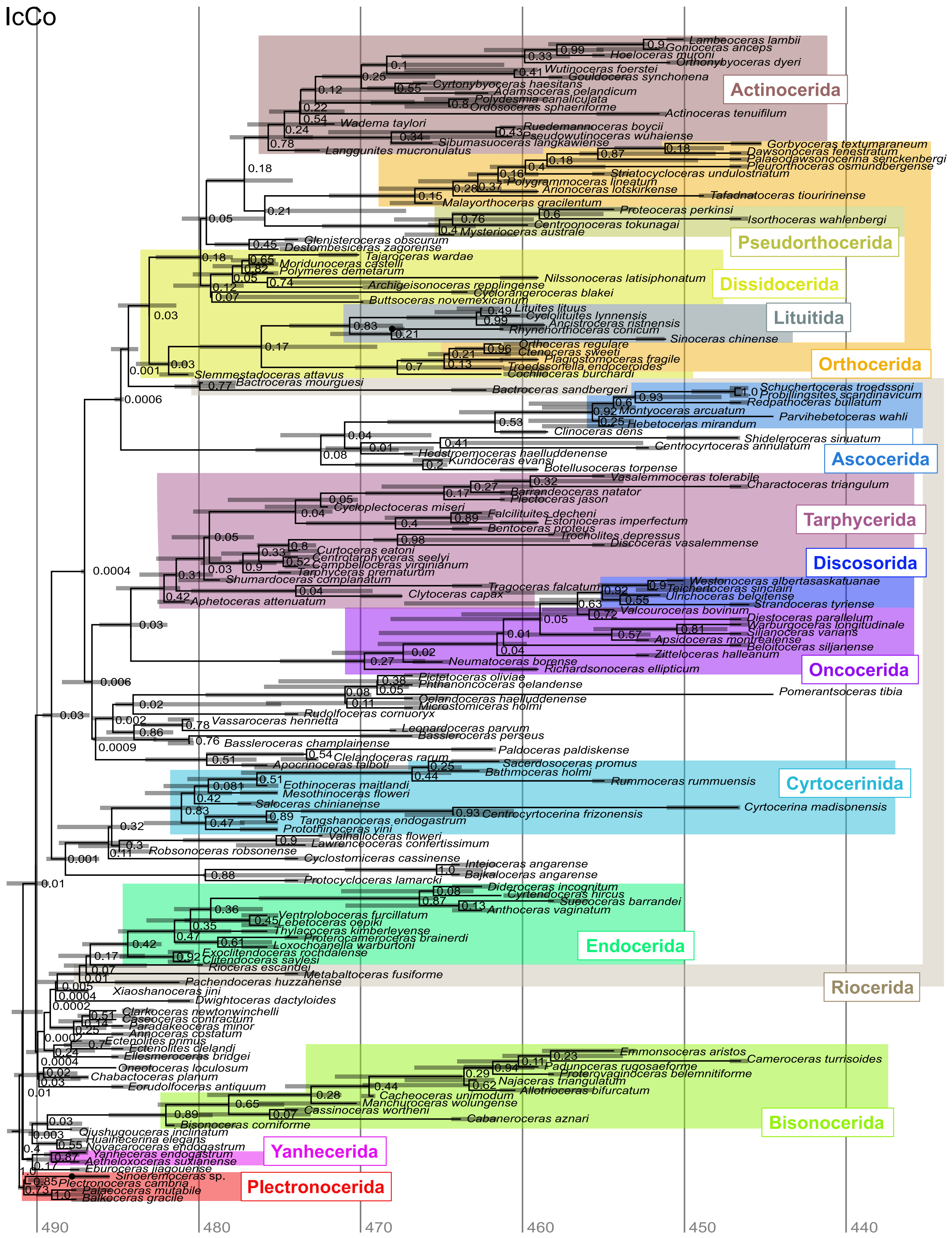
IcCo
Actinocerida
Lambeoceras lambii
Gonioceras anceps
Hoeloceras muroni
Orthonybyoceras dyeri
Wutinoceras foerstei
Gouldoceras synchonena
Cyrtonybyoceras haesitans
Adamsoceras oelandicum
Polydesmia canaliculata
Ordosoceras sphaeriforme
Actinoceras tenuifilum
Wadema taylori
Ruedemannoceras boycii
Pseudowutinoceras wuhaiense
Sibumasuoceras langkawiense
Langgunites mucronulatus
Gorbyoceras textumaraneum
Dawsonoceras fenestratum
Palaeodawsonocerina senckenbergi
Pleurorthoceras osmundbergense
Striatocycloceras undulostriatum
Polygrammoceras lineatum
Arionoceras lotskirkense
Tafadnatoceras tiouririnense
Malayorthoceras gracilentum
Proteoceras perkinsi
Isorthoceras wahlenbergi
Centroonoceras tokunagai
Mysterioceras australe
Pseudorthocerida
Glenisteroceras obscurum
Destombesiceras zagorense
Tajaroceras wardae
Moridunoceras castelli
Polymeres demetarum
Dissidocerida
Nilssonoceras latisiphonatum
Archigeisonoceras repplingense
Cyclorangeroceras blakei
Buttsoceras novemexicanum
Lituites lituus
Cyclolituites lynnensis
Ancistroceras ristnensis
Rhynchorthoceras conicum
Lituitida
Sinoceras chinense
Orthoceras regulare
Ctenoceras sweeti
Plagiostomoceras fragile
Troedssonella endoceroides
Cochlioceras burchardi
Orthocerida
Slemmestadoceras attavus
Bactroceras mourguesi
Bactroceras sandbergeri
Schuchertoceras troedssoni
Probillingsites scandinavicum
Redpathoceras bullatum
Montyoceras arcuatum
Parvihebetoceras wahli
Hebetoceras mirandum
Clinoceras dens
Shideleroceras sinuatum
Centrocyrtoceras annulatum
Ascocerida
Hedstroemoceras haelluddenense
Kundoceras evansi
Botellusoceras torpense
Vasalemmoceras tolerabile
Charactoceras triangulum
Barrandeoceras natator
Plectoceras jason
Cycloplectoceras miseri
Tarphycerida
Falcilituites decheni
Estonioceras imperfectum
Bentoceras proteus
Trocholites depressus
Discoceras vasalemmense
Curtoceras eatoni
Centrotarphyceras seelyi
Campbelloceras virginianum
Tarphyceras prematurum
Shumardoceras complanatum
Discosorida
Tragoceras falcatum
Westonoceras albertasaskatuanae
Teichertoceras sinclairi
Ulrichoceras beloitense
Strandoceras tyriense
Clytoceras capax
Aphetoceras attenuatum
Valcouroceras bovinum
Diestoceras parallelum
Warburgoceras longitudinale
Siljanoceras varians
Apsidoceras montrealense
Beloitoceras siljanense
Zitteloceras halleanum
Neumatoceras borense
Richardsonoceras ellipticum
Oncocerida
Pictetoceras oliviae
Phthanonceras oelandense
Pomerantsoceras tibia
Oelandoceras haelluddenense
Microstomiceras holmi
Rudolfoceras cornuoryx
Vassaroceras henrietta
Leonardoceras parvum
Bassleroceras perseus
Bassleroceras champlainense
Paldoceras paldiskense
Clelandoceras rarum
Apocrinoceras talboti
Sacerdosoceras promus
Bathmoceras holmi
Rummoceras rummuensis
Cyrtocerinida
Eothinoceras maitlandi
Mesothinoceras floweri
Saloceras chinianense
Cyrtocerina madisonensis
Centrocyrtocerina frizonensis
Tangshanoceras endogastrum
Protothinoceras yini
Valhalloceras floweri
Lawrenceoceras confertissimum
Robsonoceras robsonense
Cyclostomiceras cassinense
Intejoceras angarense
Bajkaloceras angarense
Protocycloceras lamarcki
Dideroceras incognitum
Cyrtendoceras hircus
Suecoceras barrandei
Anthoceras vaginatum
Ventroloboceras furcillatum
Lebetoceras oepiki
Thylacoceras kimberleyense
Proterocameroceras brainerdi
Loxochoanella warburtoni
Endocerida
Exoclitendoceras rochdalense
Clitendoceras saylesi
Rioceras escandei
Metabaltoceras fusiforme
Pachendoceras huzzahense
Riocerida
Xiaoshanoceras jini
Dwightoceras dactyloides
Clarkoceras newtonwinchelli
Caseoceras contractum
Paradakeoceras minor
Annoceras costatum
Ectenolites primus
Ectenolites clelandi
Ellesmeroceras bridgei
Emmonsoceras aristos
Cameroceras turrisoides
Padunoceras rugosaeforme
Proterovaginoceras belemnitiforme
Najaceras triangulatum
Allotrioceras bifurcatum
Oneotoceras loculosum
Chabactoceras planum
Eorudolfoceras antiquum
Cacheoceras unimodum
Manchuroceras wolungense
Cassinoceras wortheni
Cabaneroceras aznari
Bisonoceras corniforme
Bisonocerida
Qiushugouceras inclinatum
Huaihecerina elegans
Novacaroceras endogastrum
Yanhecerida
Yanheceras endogastrum
Aetheloxoceras suxianense
Eburoceras jiagouense
Sinoeremoceras sp.
Plectronoceras cambria
Palaeoceras mutabile
Balkoceras gracile
Plectronocerida
490
480
470
460
450
440

Fig. S8. Full MCC tree of the IcCo analysis.

This analysis excluded incompletely sampled characters. Numbers at nodes represent posterior probabilities. Colored boxes represent orders. Some species were recovered as sampled ancestors, represented here by black dots.

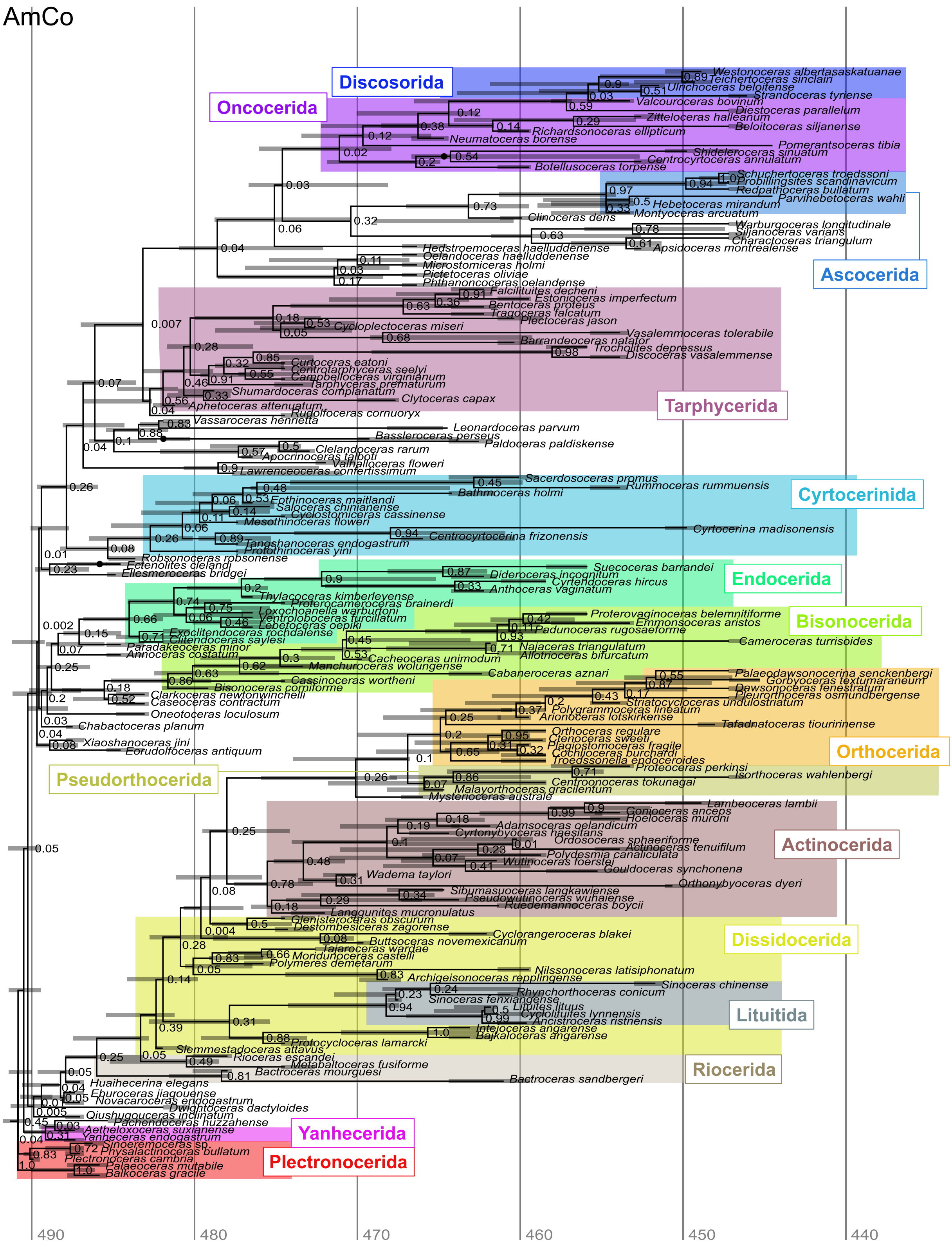
AmCo
Discosorida
Oncocerida
Ascocerida
Tarphycerida
Cyrtocerinida
Endocerida
Bisonocerida
Orthocerida
Pseudorthocerida
Actinocerida
Dissidocerida
Lituitida
Riocerida
Yanhecerida
Plectronocerida
Westonoceras albertasaskatuanae
Teichertoceras sinclairi
Ulrichoceras beloitense
Strandoceras tyriense
Valcouroceras bovinum
Diestoceras parallelum
Zitteloceras hallianum
Beloitoceras siljanense
Richardsonoceras ellipticum
Neumatoceras borense
Pomerantsoceras tibia
Shideleroceras sinuatum
Centrocyrtoceras annulatum
Botellusoceras torpense
Schuchertoceras troedssoni
Probillingsites scandinavicum
Redpathoceras bullatum
Parvihebetoceras wahli
Hebetoceras mirandum
Montyoceras arcuatum
Clinoceras dens
Warburgoceras longitudinale
Siljanoceras varians
Charactoceras triangulum
Apsidoceras montrealense
Hedstroemoceras haelluddenense
Oelandoceras haelluddenense
Microstomiceras holmi
Pictetoceras oliviae
Phthanoncoceras oelandense
Falcilituites decheni
Estonioceras imperfectum
Bentoceras proteus
Tragoceras falcatum
Plectoceras jason
Cycloplectoceras miseri
Vasalemmoceras tolerabile
Barrandeoceras natator
Trocholites depressus
Discoceras vasalemmense
Curtoceras eatoni
Centrotarphyceras seelyi
Campbelloceras virginianum
Tarphyceras prematurum
Shumardoceras complanatum
Clytoceras capax
Aphetoceras attenuatum
Rudolfoceras cornuoryx
Vassaroceras henrietta
Leonardoceras parvum
Bassleroceras perseus
Paldoceras paldiskense
Clelandoceras rarum
Apocrinoceras talboti
Valhalloceras floweri
Lawrenceoceras confertissimum
Sacerdosoceras promus
Rummoceras rummuensis
Bathmoceras holmi
Eothinoceras maitlandi
Saloceras chinianense
Cyclostomiceras cassinense
Mesothinoceras floweri
Centrocyrtocerina frizonensis
Cyrtocerina madisonensis
Tangshanoceras endogastrum
Protothinoceras yini
Robsonoceras robsonense
Ectenolites clelandi
Ellesmeroceras bridgei
Suecoceras barrandei
Dideroceras incognitum
Cyrtendoceras hircus
Anthoceras vaginatum
Thylacoceras kimberleyense
Proterocameroceras brainerdi
Loxochoanella warburtoni
Ventroloboceras furcillatum
Lebetoceras oepiki
Proterovaginoceras belemnitiforme
Emmonsoceras aristos
Padunoceras rugosaeforme
Cameroceras turrisoides
Exoclitendoceras rochdalense
Clitendoceras saylesi
Paradakeoceras minor
Annoceras costatum
Najaceras triangulatum
Allotrioceras bifurcatum
Cacheoceras unimodum
Manchuroceras wolungense
Cabaneroceras aznari
Cassinoceras wortheni
Bisonoceras corniforme
Palaeodawsonocerina senckenbergi
Gorbyoceras textumaraneum
Dawsonoceras fenestratum
Pleurorthoceras osmundbergense
Clarkoceras newtonwinchelli
Caseoceras contractum
Striatocycloceras undulostriatum
Polygrammoceras lineatum
Oneotoceras loculosum
Arionoceras lotskirkense
Tafadnatoceras tiouririnense
Chabactoceras planum
Orthoceras regulare
Ctenoceras sweeti
Xiaoshanoceras jiini
Plagiostomoceras fragile
Eorudolfoceras antiquum
Cochlioceras burchardi
Troedssonella endoceroides
Proteoceras perkinsi
Isorthoceras wahlenbergi
Centroonoceras tokunagai
Malayorthoceras gracilentum
Mysterioceras australe
Lambeoceras lambii
Gonioceras anceps
Hoeloceras muroni
Adamsoceras oelandicum
Cyrtonybyoceras haesitans
Ordosoceras sphaeriforme
Actinoceras tenuifilum
Polydesmia canaliculata
Wutinoceras foerstei
Gouldoceras synchonena
Wadema taylori
Orthonybyoceras dyeri
Sibumasuoceras langkawiense
Pseudowutinoceras wuhaiense
Ruedemannoceras boycii
Langgunites mucronulatus
Glenisteroceras obscurum
Destombesiceras zagorense
Cyclorangeroceras blakei
Buttsoceras novemexicanum
Tajaroceras wardae
Moridunoceras castelli
Polymeres demetarum
Nilssonoceras latisiphonatum
Archigeisonoceras repplingense
Sinoceras chinense
Rhynchorthoceras conicum
Sinoceras fenxiangense
Lituites lituus
Cyclolituites lynnensis
Ancistroceras ristnensis
Intejoceras angarense
Baikaloceras angarense
Protocycloceras lamarcki
Slemmestadoceras attavus
Rioceras escandei
Metabaltoceras fusiforme
Bactroceras mourguesi
Bactroceras sandbergeri
Huaihecerina elegans
Eburoceras jiagouense
Novacaroceras endogastrum
Dwightoceras dactyloides
Qiushugouceras inclinatum
Pachendoceras huzzahense
Aetheloxoceras suxianense
Yanheceras endogastrum
Sinoeremoceras sp.
Physalactinoceras bullatum
Plectronoceras cambria
Palaeoceras mutabile
Balkoceras gracile
490
480
470
460
450
440

Fig. S9. Full MCC tree of the AmCo analysis.

This analysis excluded autapomorphic characters. Numbers at nodes represent posterior probabilities. Colored boxes represent orders. Some species were recovered as sampled ancestors, represented here by black dots.

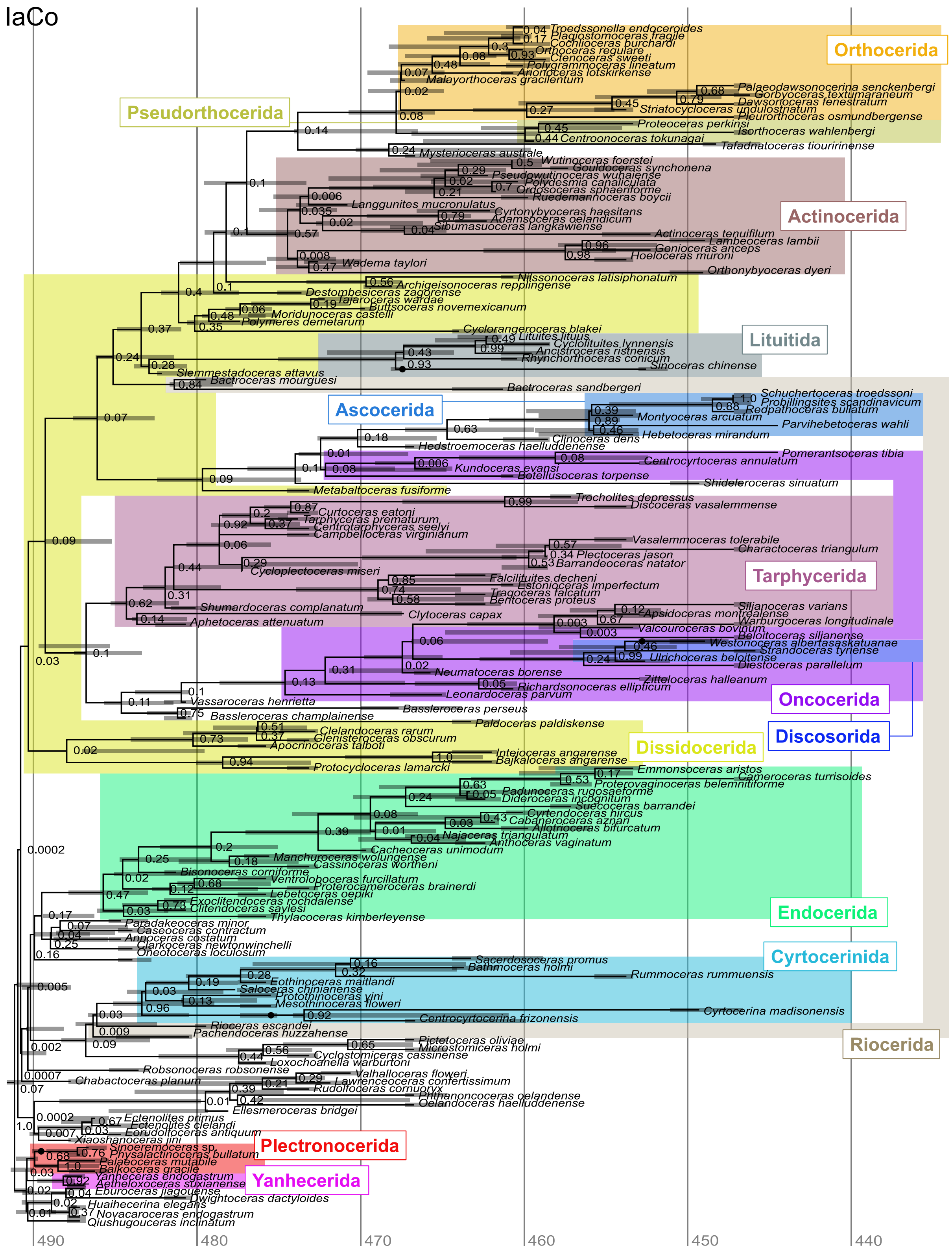

IaCo
Orthocerida
Pseudorthoceridae
Pseudorthocerida
Actinocerida
Lituitida
Ascocerida
Tarphycerida
Oncocerida
Discosorida
Dissidocerida
Endocerida
Cyrtocerinida
Riocerida
Plectronocerida
Yanhecerida
Troedssonella endoceroides
Plagiostomoceras fragile
Cochlioceras burchardi
Orthoceras regulare
Ctenoceras sweeti
Polygrammoceras lineatum
Arionoceras lotskirkense
Malayorthoceras gracilentum
Palaeodawsonocerina senckenbergi
Gorbyoceras textumaraneum
Dawsonoceras fenestratum
Striatocycloceras undulostriatum
Pleurorthoceras osmundbergense
Proteoceras perkinsi
Isorthoceras wahlenbergi
Centroonoceras tokunagai
Tafadnatoceras tiouririnense
Mysterioceras australe
Wutinoceras foerstei
Gouldoceras synchonena
Pseudowutinoceras wuhaiense
Polydesmia canaliculata
Ordosoceras sphaeriforme
Ruedemannoceras boycii
Langgunites mucronulatus
Cyrtonybyoceras haesitans
Adamsoceras oelandicum
Sibumasuoceras langkawiense
Actinoceras tenuifilum
Lambeoceras lambii
Gonioceras anceps
Hoeloceras muroni
Wadema taylori
Orthonybyoceras dyeri
Nilssonoceras latisiphonatum
Archigeisonoceras repplingense
Destombesiceras zagorense
Talaroceras wardae
Buttsoceras novemexicanum
Moridunoceras castelli
Polymeres demetarum
Cyclorangeroceras blakei
Lituites lituus
Cyclolituites lynnensis
Ancistroceras ristnensis
Rhynchorthoceras conicum
Sinoceras chinense
Slemmestadoceras attavus
Bactroceras mourguesi
Bactroceras sandbergeri
Schuchertoceras troedssoni
Probillingsites scandinavicum
Redpathoceras bullatum
Montyoceras arcuatum
Parvihebetoceras wahli
Clinoceras dens
Hebetoceras mirandum
Hedstroemoceras haelluddenense
Pomerantsoceras tibia
Centrocyrtoceras annulatum
Kundoceras evansi
Botellusoceras torpense
Shideleroceras sinuatum
Metabaltoceras fusiforme
Trocholites depressus
Discoceras vasalemmense
Curtoceras eatoni
Tarphyceras prematurum
Centrotarphyceras seelyi
Campbelloceras virginianum
Vasalemmoceras tolerabile
Charactoceras triangulum
Plectoceras jason
Barrandeoceras natator
Cycloplectoceras miseri
Falcilituites decheni
Estonioceras imperfectum
Tragoceras falcatum
Bentoceras proteus
Shumardoceras complanatum
Clytoceras capax
Aphetoceras attenuatum
Siljanoceras varians
Apsidoceras montrealense
Warburgoceras longitudinale
Valcouroceras bovinum
Beloitoceras siljanense
Westonoceras albertasaskatuanae
Strandoceras tyriense
Ulrichoceras beloitense
Diestoceras parallelum
Neumatoceras borense
Zittelloceras halleanum
Richardsonoceras ellipticum
Leonardoceras parvum
Vassaroceras henrietta
Bassleroceras perseus
Bassleroceras champlainense
Paldoceras paldiskense
Clelandoceras rarum
Glenisteroceras obscurum
Apocrinoceras talboti
Intejoceras angarense
Bajkaloceras angarense
Protocycloceras lamarcki
Emmonsoceras aristos
Cameroceras turrisoides
Proterovaginoceras belemnitiforme
Padunoceras rugosaeforme
Dideroceras incognitum
Suecoceras barrandei
Cyrtendoceras hircus
Cabaneroceras aznari
Allotrioceras bifurcatum
Najaceras triangulatum
Anthoceras vaginatum
Cacheoceras unimodum
Manchuroceras wolungense
Cassinoceras wortheni
Bisonoceras corniforme
Ventroloboceras furcillatum
Proterocameroceras brainerdi
Lebetoceras oepiki
Exoclitendoceras rochdalense
Clitendoceras saylesi
Thylacoceras kimberleyense
Paradakeoceras minor
Caseoceras contractum
Annoceras costatum
Clarkoceras newtonwinchelli
Oneotoceras loculosum
Sacerdosoceras promus
Bathmoceras holmi
Rummoceras rummuensis
Eothinoceras maitlandi
Saloceras chinianense
Protothinoceras vini
Mesothinoceras floweri
Cyrtocerina madisonensis
Centrocyrtocerina frizonensis
Rioceras escandei
Pachendoceras huzzahense
Pictetoceras oliviae
Microstomiceras holmi
Cyclostomiceras cassinense
Loxochoanella warburtoni
Robsonoceras robsonense
Valhalloceras floweri
Chabactoceras planum
Lawrenceoceras confertissimum
Rudolfoceras cornuoryx
Pithanonceras oelandense
Oelandoceras haelluddenense
Ellesmeroceras bridgei
Ectenolites primus
Ectenolites clelandi
Eburoidoceras antiquum
Xiaoshanoceras jini
Sinoeremoceras sp.
Physalactinoceras bullatum
Palaeoceras mutabile
Balkoceras gracile
Yanheceras endogastrum
Aetheloxoceras suxianense
Eburoceras jiagouense
Dwightoceras dactyloides
Huaihecerina elegans
Novacaroceras endogastrum
Qiushugouceras inclinatum
490
480
470
460
450
440

Fig. S10. Full MCC tree of the IaCo analysis.

This analysis excluded characters that contained high amounts of inapplicable data (gaps). Numbers at nodes represent posterior probabilities. Colored boxes represent orders. Some species were recovered as sampled ancestors, represented here by black dots.

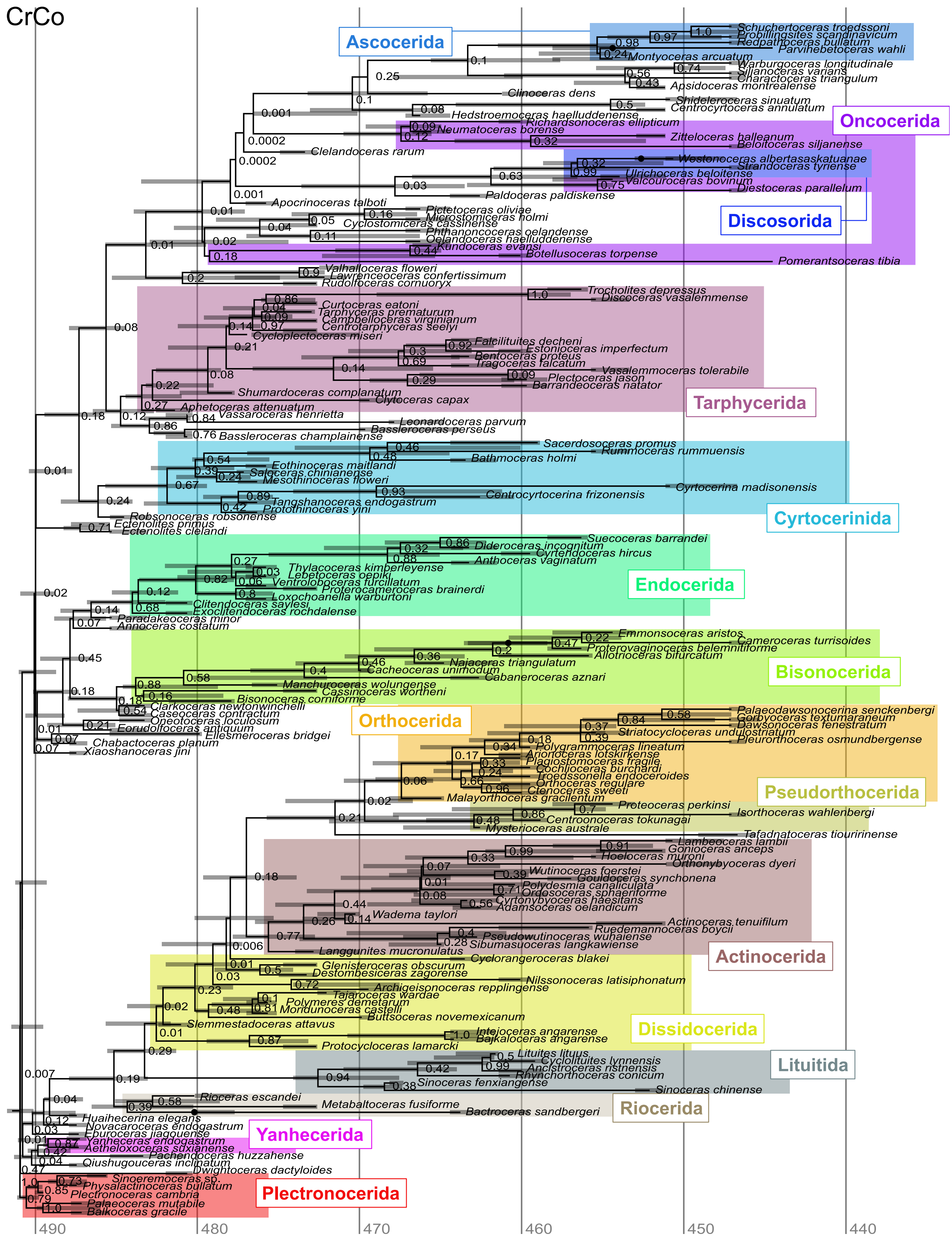
CrCo
Ascocerida
Schuchertoceras troedssoni
Probillingsites scandinavicum
Redpathoceras bullatum
Parvihebetoceras wahli
Montyoceras arcuatum
Warburgoceras longitudinale
Siljanoceras varians
Charactoceras triangulum
Apsidoceras montrealense
Clinoceras dens
Shideleroceras sinuatum
Centrocyrtoceras annulatum
Oncocerida
Hedstroemoceras haelluddenense
Richardsonoceras ellipticum
Neumatoceras borense
Zitteloceras halleanum
Beloitoceras siljanense
Clelandoceras rarum
Westonoceras albertasaskatuanae
Strandoceras tyriense
Ulrichoceras beloitense
Valcouroceras bovinum
Diestoceras parallelum
Paldoceras paldiskense
Apocrinoceras talboti
Discosorida
Pictetoceras oliviae
Microstomiceras holmi
Cyclostomiceras cassinense
Phthanoncoceras oelandense
Oelandoceras haelluddenense
Kundoceras evansi
Botellusoceras torpense
Pomerantsoceras tibia
Valhalloceras floweri
Lawrenceoceras confertissimum
Rudolfoceras cornuoryx
Trocholites depressus
Discoceras vasalemmense
Curtoceras eatoni
Tarphyceras prematurum
Campbelloceras virginianum
Centrotarphyceras seelyi
Cycloplectoceras miseri
Falcilituites decheni
Estonioceras imperfectum
Bentoceras proteus
Tragoceras falcatum
Vasalemmoceras tolerabile
Plectoceras jason
Barrandeoceras natator
Shumardoceras complanatum
Clytoceras capax
Tarphycerida
Aphetoceras attenuatum
Vassaroceras henrietta
Leonardoceras parvum
Bassleroceras perseus
Bassleroceras champlainense
Sacerdosoceras promus
Rummoceras rummuensis
Bathmoceras holmi
Eothinoceras maitlandi
Saloceras chinianense
Mesothinoceras floweri
Cyrtocerina madisonensis
Centrocyrtocerina frizonensis
Tangshanoceras endogastrum
Protothinoceras yini
Cyrtocerinida
Robsonoceras robsonense
Ectenolites primus
Ectenolites clelandi
Suecoceras barrandei
Dideroceras incognitum
Cyrtendoceras hircus
Anthoceras vaginatum
Thylacoceras kimberleyense
Lebetoceras oepiki
Ventroloboceras furcillatum
Proterocameroceras brainerdi
Loxochoanella warburtoni
Endocerida
Clitendoceras saylesi
Exoclitendoceras rochdalense
Paradakeoceras minor
Annoceras costatum
Emmonsoceras aristos
Cameroceras turrisoides
Proterovaginoceras belemnitiforme
Allotrioceras bifurcatum
Najaceras triangulatum
Cacheoceras unimodum
Cabaneroceras aznari
Bisonocerida
Manchuroceras wolungense
Cassinoceras wortheni
Bisonoceras corniforme
Clarkoceras newtonwinchelli
Caseoceras contractum
Orthocerida
Oneotoceras loculosum
Eorudolfoceras antiquum
Ellesmeroceras bridgei
Chabactoceras planum
Xiaoshanoceras jini
Palaeodawsonocerina senckenbergi
Gorbyoceras textumaraneum
Dawsonoceras fenestratum
Striatocycloceras undulostriatum
Pleurorthoceras osmundbergense
Polygrammoceras lineatum
Arionoceras lotskirkense
Plagiostomoceras fragile
Cochlioceras burchardi
Troedssonella endoceroides
Orthoceras regulare
Ctenoceras sweeti
Malayorthoceras gracilentum
Pseudorthocerida
Proteoceras perkinsi
Isorthoceras wahlenbergi
Centroonoceras tokunagai
Mysterioceras australe
Tafadnatoceras tiouririnense
Lambeoceras lambii
Gonioceras anceps
Hoeloceras muroni
Orthonybyoceras dyeri
Wutinoceras foerstei
Gouldoceras synchonena
Polydesmia canaliculata
Ordosoceras sphaeriforme
Cyrtonybyoceras haesitans
Adamsoceras oelandicum
Wadema taylori
Actinoceras tenuifilum
Ruedemannoceras boycii
Pseudowutinoceras wuhaiense
Sibumasuoceras langkawiense
Langgunites mucronulatus
Actinocerida
Cyclorangeroceras blakei
Glenisteroceras obscurum
Destombesiceras zagorense
Nilssonoceras latisiphonatum
Archigeisonoceras repplingense
Tajaroceras wardae
Polymeres demetarum
Moridunoceras castelli
Buttsoceras novemexicanum
Slemmestadoceras attavus
Dissidocerida
Intejoceras angarense
Bajkaloceras angarense
Protocycloceras lamarcki
Lituites lituus
Cyclolituites lynnensis
Ancistroceras ristnensis
Rhynchorthoceras conicum
Lituitida
Sinoceras fenxiangense
Sinoceras chinense
Rioceras escandei
Metabaltoceras fusiforme
Bactroceras sandbergeri
Riocerida
Huaihecerina elegans
Novacaroceras endogastrum
Eburoceras jiagouense
Yanheceras endogastrum
Yanhecerida
Aetheloxoceras sdxianense
Pachendoceras huzzahense
Qiushugouceras inclinatum
Dwightoceras dactyloides
Sinoeremoceras sp.
Physalactinoceras bullatum
Plectronoceras cambria
Plectronocerida
Palaeoceras mutabile
Balkoceras gracile
490
480
470
460
450
440

Fig. S11. Full MCC tree of the CrCo analysis.

This analysis included an alternative, speculatively scored connecting ring type. Numbers at nodes represent posterior probabilities. Colored boxes represent orders. Some species were recovered as sampled ancestors, represented here by black dots.

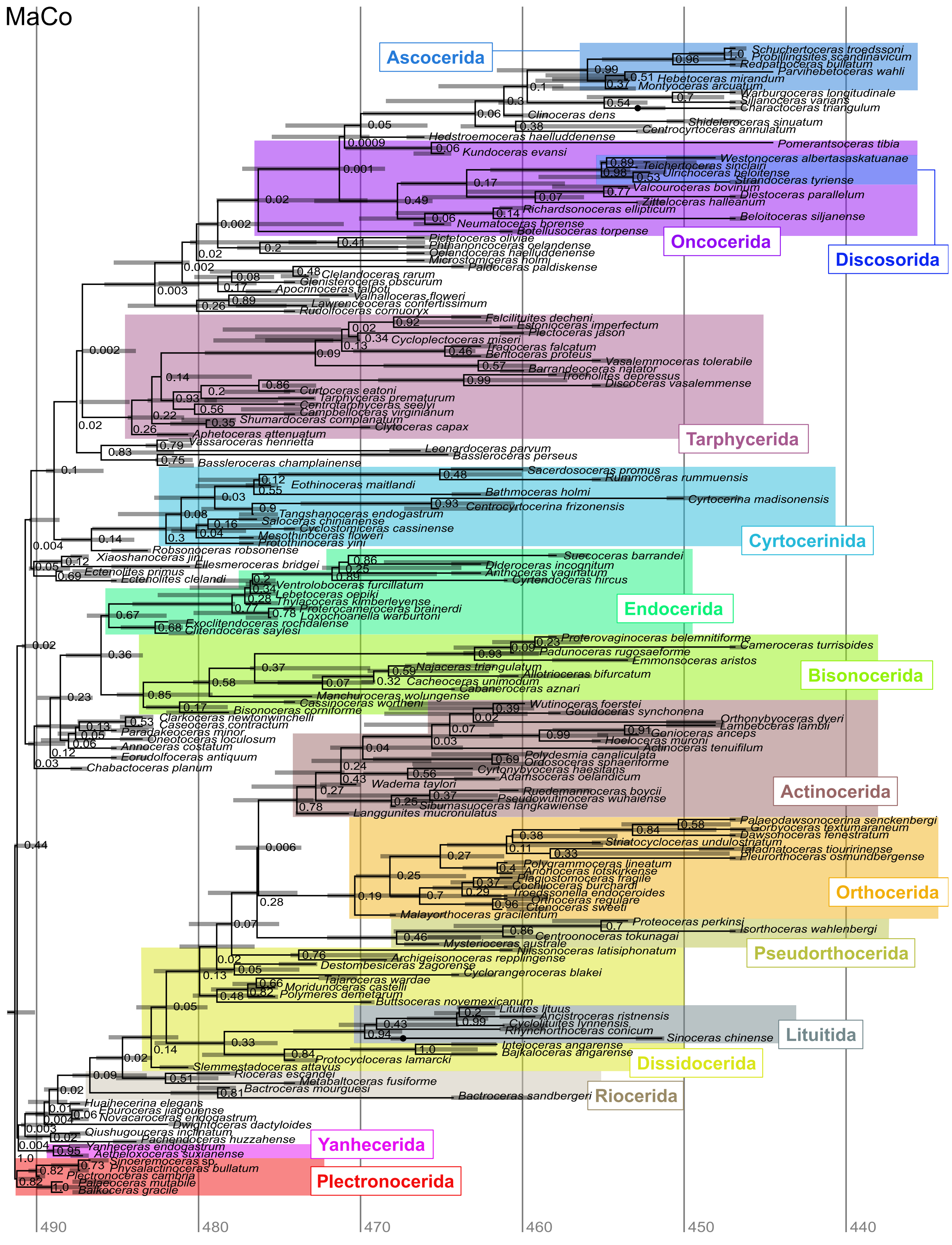

MaCo
Ascocerida
Schuchertoceras troedssoni
Probillingsites scandinavicum
Redpathoceras bullatum
Parvihebetoceras wahli
Hebetoceras mirandum
Montyoceras arcuatum
Warburgoceras longitudinale
Siljanoceras varians
Characteoceras triangulum
Clinoceras dens
Shideleroceras sinuatum
Centrocyrtoceras annulatum
Hedstroemoceras haelluddenense
Pomerantsoceras tibia
Kundoceras evansi
Westonoceras albertasaskatuanae
Teichertoceras sinclairi
Ulrichoceras beloitense
Strandoceras tyriense
Valcouroceras bovinum
Diestoceras parallelum
Zitteloceras halleanum
Richardsonoceras ellipticum
Beloitoceras siljanense
Neumatoceras borense
Bottellusoceras torpense
Oncocerida
Discosorida
Pictetoceras oliviae
Phthanoncoceras oelandense
Oelandoceras haelluddenense
Microstomiceras holmi
Paldoceras paldiskense
Clelandoceras rarum
Glenisteroceras obscurum
Apocrinoceras talboti
Valhalloceras floweri
Lawrenceoceras confertissimum
Rudolfoceras cornuoryx
Falcilituites decheni
Estonioceras imperfectum
Plectoceras jason
Cycloplectoceras miseri
Tragoceras falcatum
Bentoceras proteus
Vasalemmoceras tolerabile
Barrandeoceras natator
Trocholites depressus
Discoceras vasalemmense
Curtoceras eatoni
Tarphyceras prematurum
Centrotarphyceras seelyi
Campbelloceras virginianum
Shumardoceras complanatum
Clytoceras capax
Aphetoceras attenuatum
Tarphycerida
Vassaroceras henrietta
Leonardoceras parvum
Bassleroceras perseus
Bassleroceras champlainense
Sacerdosoceras promus
Rummoceras rummuensis
Eothinoceras maitlandi
Bathmoceras holmi
Cyrtocerina madisonensis
Tangshanoceras endogastrum
Centrocyrtocerina frizonensis
Saloceras chinianense
Cyclostomiceras cassinense
Mesothinoceras floweri
Protothinoceras yini
Cyrtocerinida
Robsonoceras robsonense
Xiaoshanoceras jini
Suecoceras barrandei
Ellesmeroceras bridgei
Dideroceras incognitum
Ectenolites primus
Anthoceras vaginatum
Ectenolites clelandi
Cyrtendoceras hircus
Ventroloboceras furcillatum
Lebetoceras oepiki
Thylacoceras kimberleyense
Proterocameroceras brainerdi
Loxochoanella warburtoni
Exoclitendoceras rochdalense
Clitendoceras saylesi
Endocerida
Proterovaginoceras belemnitiforme
Cameroceras turrisoides
Padunoceras rugosaeforme
Emmonsoceras aristos
Najaceras triangulatum
Allotrioceras bifurcatum
Cacheoceras unimodum
Cabaneroceras aznari
Bisonocerida
Manchuroceras wolungense
Wutinoceras foersteri
Cassinoceras wortheni
Bisonoceras corniforme
Gouldoceras synchonena
Clarkoceras newtonwinchelli
Orthonybyoceras dyeri
Caseoceras contractum
Lambeoceras lambii
Paradakeoceras minor
Gonioceras anceps
Oneotoceras loculosum
Hoeloceras muroni
Annoceras costatum
Actinoceras tenuifilum
Eorudolfoceras antiquum
Polydesmia canaliculata
Chabactoceras planum
Ordosoceras sphaeriforme
Cyrtonybyoceras haesitans
Adamsoceras oelandicum
Wadema taylori
Actinocerida
Ruedemannoceras boycii
Pseudowutinoceras wuhaiense
Sibumasuoceras langkawiense
Langgunites mucronulatus
Palaeodawsonocerina senckenbergi
Gorbyoceras textumaraneum
Dawsonoceras fenestratum
Striatocycloceras undulostriatum
Tafadnatoceras tiouririnense
Pleurorthoceras osmundbergense
Polygrammoceras lineatum
Arionoceras lotskirkense
Plagiostomoceras fragile
Cochlioceras burchardi
Orthocerida
Troedssonella endoceroides
Orthoceras regulare
Clenoceras sweeti
Malayorthoceras gracilentum
Proteoceras perkinsi
Isorthoceras wahlenbergi
Centroonoceras tokunagai
Mysterioceras australe
Pseudorthocerida
Nilssonoceras latisiphonatum
Archigeisonoceras repplingense
Destombesiceras zagorense
Cyclorangeroceras blakei
Tajaroceras wardae
Moridunoceras castelli
Polymeres demetarum
Buttsoceras novemexicanum
Lituites lituus
Ancistroceras ristnensis
Cyclolituites lynnensis
Rhynchorthoceras conicum
Sinoceras chinense
Lituitida
Intejoceras angarense
Bajkaloceras angarense
Protocycloceras lamarcki
Dissidocerida
Slemmestadoceras attavus
Rioceras escandei
Metabaltoceras fusiforme
Bactroceras mourguesi
Bactroceras sandbergeri
Riocerida
Huaiheceerina elegans
Eburoceras jiagouense
Novacaroceras endogastrum
Dwightoceras dactyloides
Qiushugouceras inclinatum
Pachendoceras huzzahense
Yanheceras endogastrum
Aetheloxoceras suxianense
Yanhecerida
Sinoeremoceras sp.
Physalactinoceras bullatum
Plectronoceras cambria
Palaeoceras mutabile
Balkoceras gracile
Plectronocerida
490
480
470
460
450
440

Fig. S12. Full MCC tree of the MaCo analysis.

This analysis included alternative, speculatively scored muscle attachment patterns. Numbers at nodes represent posterior probabilities. Colored boxes represent orders. Some species were recovered as sampled ancestors, represented here by black dots.

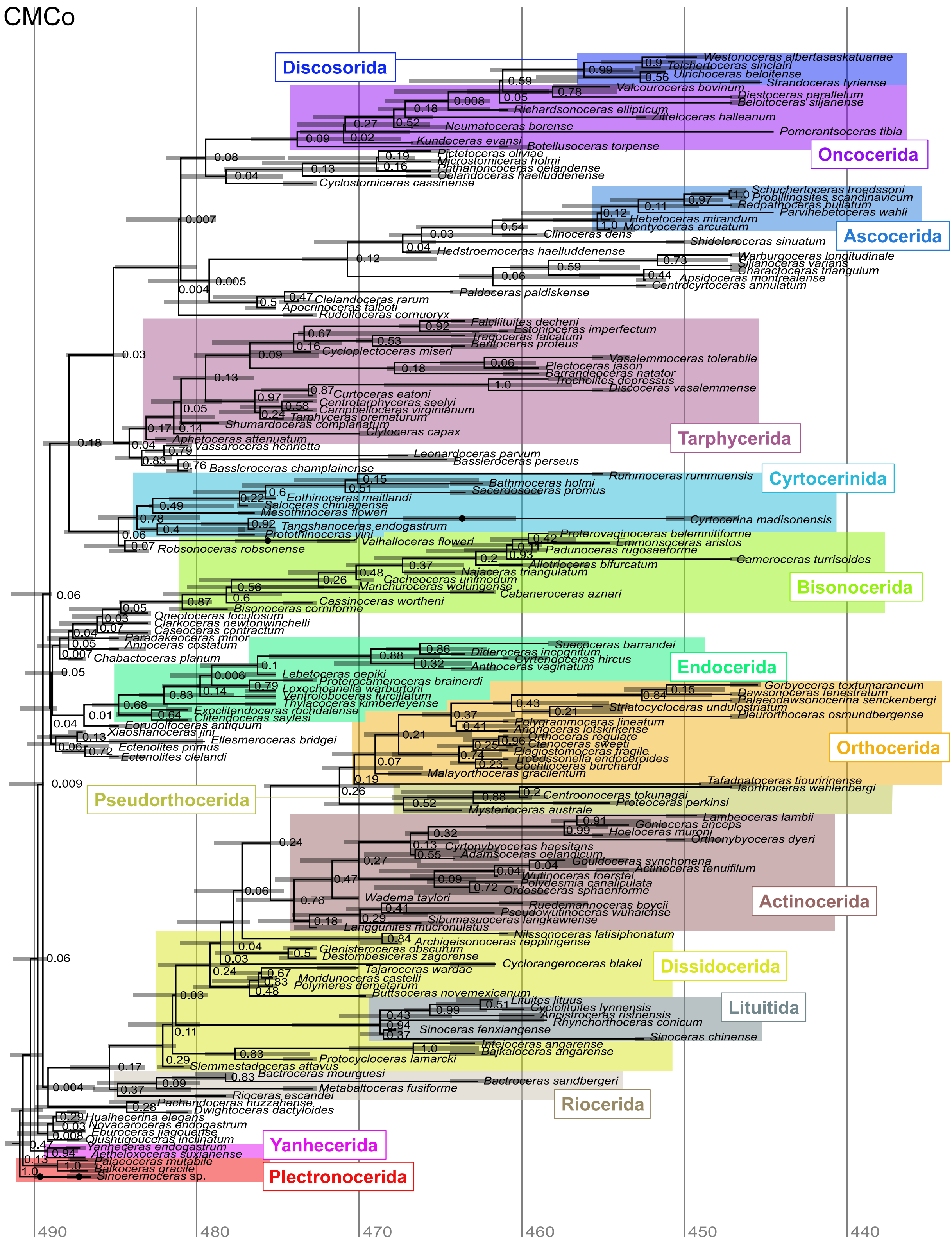
CMCo
Discosorida
Oncocerida
Ascocerida
Tarphycerida
Cyrtocerinida
Bisonocerida
Endocerida
Orthocerida
Pseudorthocerida
Actinocerida
Dissidocerida
Lituitida
Riocerida
Yanhecerida
Plectronocerida
Westonoceras albertasaskatuanae
Teichertoceras sinclairi
Ulrichoceras beloitense
Strandoceras tyriense
Valcouroceras bovinum
Diestoceras parallelum
Beloitoceras siljanense
Richardsonoceras ellipticum
Zittelloceras halleanum
Neumatoceras borense
Pomerantsoceras tibia
Kundoceras evansi
Botellusoceras torpense
Pictetoceras oliviae
Microstomiceras holmi
Ephippiorthoceras oelandense
Oelandoceras haelluddenense
Cyclostomiceras cassinense
Schuchertoceras troedssoni
Probillingsites scandinavicum
Redpathoceras bullatum
Parvihebetoceras wahli
Hebetoceras mirandum
Montyoceras arcuatum
Clinoceras dens
Shideleroceras sinuatum
Hedstroemoceras haelluddenense
Warburgoceras longitudinale
Siljanoceras varians
Charactoceras triangulum
Apsidoceras montrealense
Centrocyrtoceras annulatum
Paldoceras paldiskense
Clelandoceras rarum
Apocrinoceras talboti
Rudolfoceras cornuoryx
Falcilituites decheni
Estonioceras imperfectum
Tragoceras falcatum
Bentoceras proteus
Cycloplectoceras miseri
Vasalemmoceras tolerabile
Plectoceras jason
Barrandeoceras natator
Trocholites depressus
Discoceras vasalemmense
Curtoceras eatoni
Centrotarphyceras seelyi
Campbelloceras virginianum
Tarphyceras prematurum
Shumardoceras complanatum
Clytoceras capax
Aphetoceras attenuatum
Vassaroceras henrietta
Leonardoceras parvum
Bassleroceras perseus
Bassleroceras champlainense
Rummoceras rummuensis
Bathmoceras holmi
Sacerdosoceras promus
Eothinoceras maitlandi
Saloceras chinianense
Mesothinoceras floweri
Cyrtocerina madisonensis
Tangshanoceras endogastrum
Protothinoceras yini
Valhalloceras floweri
Robsonoceras robsonense
Proterovaginoceras belemnitiforme
Emmonsoceras aristos
Padunoceras rugosaeforme
Cameroceras turrisoides
Allotrioceras bifurcatum
Najaceras triangulatum
Cacheoceras uniimodum
Manchuroceras wolungense
Cabaneroceras aznari
Cassinoceras wortheni
Bisonoceras corniforme
Oneotoceras loculosum
Clarkoceras newtonwinchelli
Caseoceras contractum
Paradakeoceras minor
Annoceras costatum
Chabactoceras planum
Suecoceras barrandei
Dideroceras incognitum
Cyrtendoceras hircus
Anthoceras vaginatum
Lebetoceras oepiki
Proterocameroceras brainerdi
Loxochoanella warburtoni
Ventroloboceras furcillatum
Thylacoceras kimberleyense
Exocliitendoceras rochdalense
Clitendoceras saylesi
Eorudolfoceras antiquum
Xiaoshanoceras jini
Ellesmeroceras bridgei
Ectenolites primus
Ectenolites clelandi
Gorbyoceras textumaraneum
Dawsonoceras fenestratum
Palaeodawsonocerina senckenbergi
Striatocycloceras undulostriatum
Pleurorthoceras osmundbergense
Polygrammoceras lineatum
Arionoceras lotskirkense
Orthoceras regulare
Ctenoceras sweeti
Plagiostomoceras fragile
Troedssonella endoceroides
Cochlioceras burchardi
Malayorthoceras gracilentum
Tafadnatoceras tiouririnense
Isorthoceras wahlenbergi
Centroonoceras tokunagai
Proteoceras perkinsi
Mysterioceras australe
Lambeoceras lambii
Gonioceras anceps
Hoeloceras muroni
Orthonybyoceras dyeri
Cyrtonybyoceras haesitans
Adamsoceras oelandicum
Gouldoceras synchonena
Actinoceras tenuifilum
Wutinoceras foerstei
Polydesmia canaliculata
Ordosoceras sphaeriforme
Wadema taylori
Ruedemannoceras boycii
Pseudowutinoceras wuhaiense
Sibumasuoceras langkawiense
Langgunites mucronulatus
Nilssonoceras latisiphonatum
Archigeisonoceras replingense
Glenisteroceras obscurum
Destombesiceras zagorense
Cyclorangeroceras blakei
Tajaroceras wardae
Moriduonoceras castelli
Polymeres demetarum
Buttsoceras novemexicanum
Lituites lituus
Cyclolituites lynnensis
Ancistroceras ristnensis
Rhynchorthoceras conicum
Sinoceras fenxiangense
Sinoceras chinense
Intejoceras angarense
Bajkaloceras angarense
Protocycloceras lamarcki
Slemmestadoceras attavus
Bactroceras mourguesi
Bactroceras sandbergeri
Metabaltoceras fusiforme
Rioceras escandei
Pachendoceras huzzahense
Dwightoceras dactyloides
Huaihecerina elegans
Novacaroceras endogastrum
Eburoceras jiagouense
Qiushugouceras inclinatum
Yanheceras endogastrum
Aetheloxoceras suxianense
Palaeoceras mutabile
Balkoceras gracile
Sinoeremoceras sp.
490
480
470
460
450
440

Fig. S13. Full MCC tree of the CMCo analysis.

This analysis included the combined speculatively scored character sets of muscle attachment patterns and connecting ring type. Numbers at nodes represent posterior probabilities. Colored boxes represent orders. Some species were recovered as sampled ancestors, represented here by black dots.

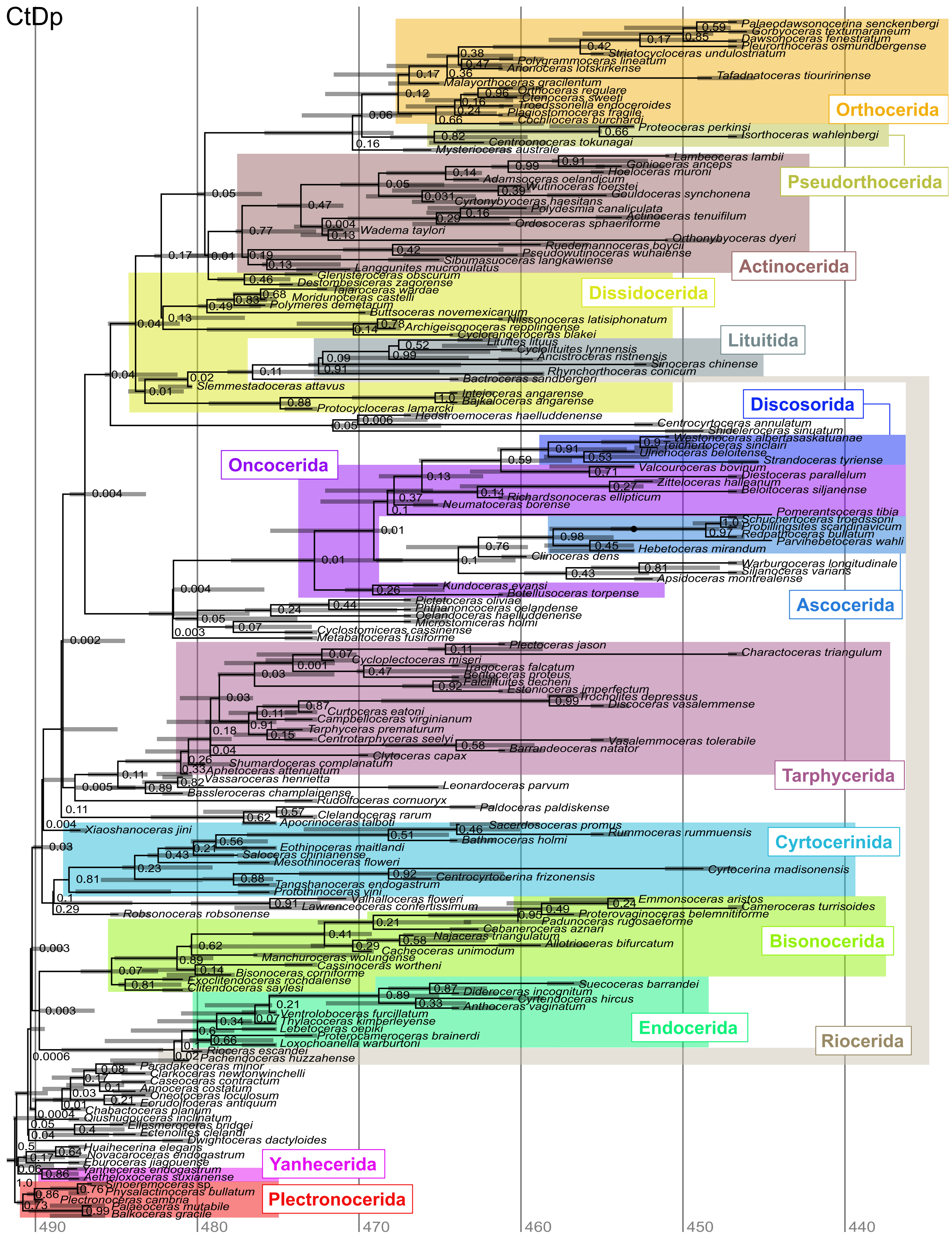
CtDp
Palaeodawsonocerina senckenbergi
Gorbyoceras textumaraneum
Dawsonoceras fenestratum
Pleurorthoceras osmundbergense
Striatocycloceras undulostriatum
Polygrammoceras lineatum
Anonoceras lotskirkense
Tafadnatoceras tiouririnense
Malayorthoceras gracilentum
Orthoceras regulare
Ctenoceras sweeti
Troedssonella endoceroides
Plagiostomoceras fragile
Cochlioceras burchardi
Orthocerida
Proteoceras perkinsi
Isorthoceras wahlenbergi
Centroonoceras tokunagai
Mysterioceras australe
Pseudorthocerida
Lambeoceras lambii
Gonioceras anceps
Hoeloceras muroni
Adamsoceras oelandicum
Wutinoceras foerstei
Gouldoceras synchonena
Cyrtonybyoceras haesitans
Polydesmia canaliculata
Actinoceras tenuifilum
Ordosoceras sphaeriforme
Wadema taylori
Orthonybyoceras dyeri
Ruedemannoceras boycii
Pseudowutinoceras wuhaiense
Sibumasuoceras langkawiense
Langgunites mucronulatus
Actinocerida
Glenisteroceras obscurum
Destombesiceras zagorense
Tajaroceras wardae
Moridunoceras castelli
Polymeres demetarum
Dissidocerida
Buttsoceras novemexicanum
Nilssonoceras latisiphonatum
Archigeisonoceras repplingense
Cyclorangeroceras blakei
Lituitida
Lituites lituus
Cyclolituites lynnensis
Ancistroceras ristnensis
Sinoceras chinense
Rhynchorthoceras conicum
Bactroceras sandbergeri
Slemmestadoceras attavus
Intejoceras angarense
Baikaloceras angarense
Protocycloceras lamarcki
Discosorida
Hedstroemoceras haelluddenense
Centrocyrtoceras annulatum
Shideleroceras sinuatum
Westonoceras albertasaskatuanae
Teichertoceras sinclairi
Ulrichoceras beloitense
Strandoceras tyriense
Oncocerida
Valcouroceras bovinum
Diestoceras parallelum
Zitteloceras hallianum
Beloitoceras siljanense
Richardsonoceras ellipticum
Neumatoceras borense
Pomerantsoceras tibia
Schuchertoceras troedssoni
Probillingsites scandinavicum
Redpathoceras bullatum
Parvihebetoceras wahli
Hebetoceras mirandum
Clinoceras dens
Warburgoceras longitudinale
Siljanoceras varians
Apsidoceras montrealense
Kundoceras evansi
Botellusoceras torpense
Ascocerida
Pictetoceras oliviae
Phthanoncoceras oelandense
Oelandoceras haelluddenense
Microstomiceras holmi
Cyclostomiceras cassinense
Metabaltoceras fusiforme
Plectoceras jason
Charactoceras triangulum
Cycloplectoceras miseri
Tragoceras falcatum
Bentoceras proteus
Falcilituites decheni
Estonioceras imperfectum
Trocholites depressus
Discoceras vasalemmense
Curtoceras eatoni
Campbelloceras virginianum
Tarphyceras prematurum
Centrotarphyceras seelyi
Vasalemmoceras tolerabile
Barrandeoceras natator
Clytoceras capax
Shumardoceras complanatum
Aphetoceras attenuatum
Vassaroceras henrietta
Tarphycerida
Leonardoceras parvum
Bassleroceras champlainense
Rudolfoceras cornuoryx
Paldoceras paldiskense
Clelandoceras rarum
Apocrinoceras talboti
Xiaoshanoceras jini
Sacerdosoceras promus
Rummoceras rummuensis
Bathmoceras holmi
Cyrtocerinida
Eothinoceras maitlandi
Saloceras chinianense
Mesothinoceras floweri
Cyrtocerina madisonensis
Centrocyrtocerina frizonensis
Tangshanoceras endogastrum
Protothinoceras yini
Valhalloceras floweri
Lawrenceoceras confertissimum
Robsonoceras robsonense
Emmonsoceras aristos
Cameroceras turrisoides
Proterovaginoceras belemnitiforme
Padunoceras rugosaeforme
Cabaneroceras aznari
Najaceras triangulatum
Allotrioceras bifurcatum
Bisonocerida
Cacheoceras unimodum
Manchuroceras wolungense
Cassinoceras wortheni
Bisonoceras corniforme
Exoclitendoceras rochdalense
Clitendoceras saylesi
Suecoceras barrandei
Dideroceras incognitum
Cyrtendoceras hircus
Anthoceras vaginatum
Ventroloboceras furcillatum
Thylacoceras kimperleyense
Lebetoceras oepiki
Endocerida
Proterocameroceras brainerdi
Loxochoanella warburtoni
Rioceras escandei
Riocerida
Pachendoceras huzzahense
Paradakeoceras minor
Clarkoceras newtonwinchelli
Caseoceras contractum
Annoceras costatum
Oneotoceras loculosum
Eorudolfoceras antiquum
Chabactoceras planum
Qiushugouceras inclinatum
Ellesmeroceras bridgei
Ectenolites clelandi
Dwightoceras dactyloides
Huaiheceras elegans
Novacaroceras endogastrum
Eburoceras jiagouense
Yanhecerida
Yanheceras endogastrum
Aetheloxoceras suxianense
Sinoeremoceras sp.
Physalactinoceras bullatum
Plectronocerida
Plectronoceras cambria
Palaeoceras mutabile
Balkoceras gracile
490
480
470
460
450
440

Fig. S14. Full MCC tree of the CtDp analysis.

This analysis excluded pseudoduplicate species. Numbers at nodes represent posterior probabilities. Colored boxes represent orders. Some species were recovered as sampled ancestors, represented here by black dots.

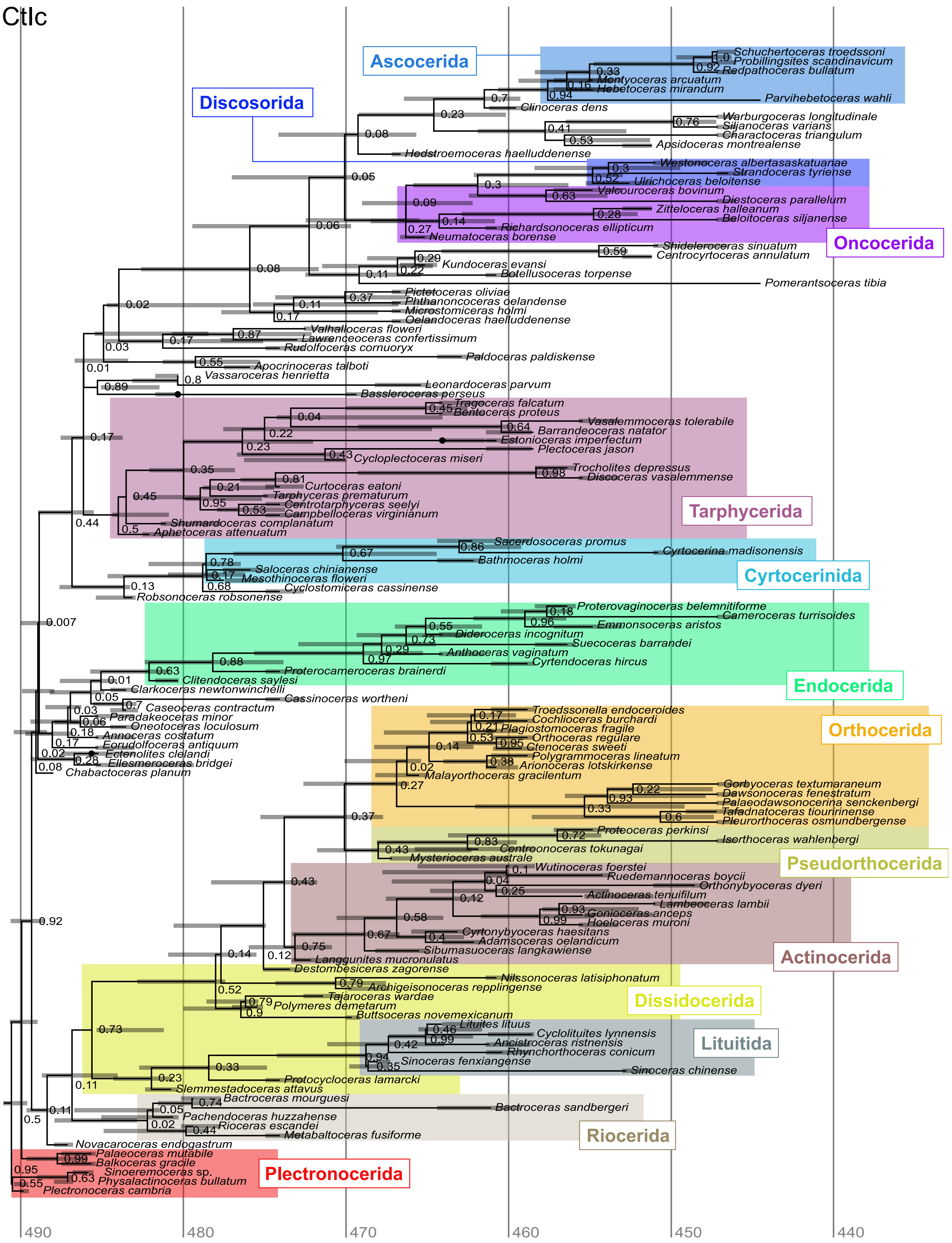
CtIc
Ascocerida
Discosorida
Oncocerida
Tarphycerida
Cyrtocerinida
Endocerida
Orthocerida
Pseudorthocerida
Actinocerida
Dissidocerida
Lituitida
Riocerida
Plectronocerida
Schuchertoceras troedssoni
Probillingsites scandinavicum
Redpathoceras bullatum
Montyoceras arcuatum
Hebetoceras mirandum
Parvihebetoceras wahli
Clinoceras dens
Warburgoceras longitudinale
Siljanoceras varians
Charactoceras triangulum
Apsidoceras montrealense
Hedstroemoceras haelluddenense
Westonoceras albertasaskatuanae
Strandoceras tyriense
Ulrichoceras beloitense
Valcouroceras bovinum
Diestoceras parallelum
Zitteloceras halleanum
Beloitoceras siljanense
Richardsonoceras ellipticum
Neumatoceras borense
Shideleroceras sinuatum
Centrocyrtoceras annulatum
Kundoceras evansi
Botellusoceras torpense
Pomerantsoceras tibia
Pictetoceras oliviae
Phthanoncoceras oelandense
Microstomiceras holmi
Oelandoceras haelluddenense
Valhalloceras floweri
Lawrenceoceras confertissimum
Rudolfoceras cornuoryx
Paldoceras paldiskense
Apocrinoceras talboti
Vassaroceras henrietta
Leonardoceras parvum
Bassleroceras perseus
Tragoceras falcatum
Bentoceras proteus
Vasalemmoceras tolerabile
Barrandeoceras natator
Estonioceras imperfectum
Plectoceras jason
Cycloplectoceras miseri
Trocholites depressus
Discoceras vasalemmense
Curtoceras eatoni
Tarphyceras prematurum
Centrotarphyceras seelyi
Campbelloceras virginianum
Shumardoceras complanatum
Aphetoceras attenuatum
Sacerdosoceras promus
Cyrtocerina madisonensis
Bathmoceras holmi
Saloceras chinianense
Mesothinoceras floweri
Cyclostomiceras cassinense
Robsonoceras robsonense
Proterovaginoceras belemnitiforme
Cameroceras turrisoides
Emmonsoceras aristos
Dideroceras incognitum
Suecoceras barrandei
Anthoceras vaginatum
Cyrtendoceras hircus
Proterocameroceras brainerdi
Clitendoceras saylesi
Clarkoceras newtonwinchelli
Cassinoceras wortheni
Caseoceras contractum
Paradakeoceras minor
Oneotoceras loculosum
Annoceras costatum
Eorudolfoceras antiquum
Ectenolites clelandi
Ellesmeroceras bridgei
Chabactoceras planum
Troedssonella endoceroides
Cochlioceras burchardi
Plagiostomoceras fragile
Orthoceras regulare
Ctenoceras sweeti
Polygrammoceras lineatum
Arionoceras lotskirkense
Malayorthoceras gracilentum
Gorbyoceras textumaraneum
Dawsonoceras fenestratum
Palaeodawsonocerina senckenbergi
Tafadnatoceras tiouririnense
Pleurorthoceras osmundbergense
Proteoceras perkinsi
Isorthoceras wahlenbergi
Centroonoceras tokunagai
Mysterioceras australe
Wutinoceras foerstei
Ruedemannoceras boycii
Orthonybyoceras dyeri
Actinoceras tenuifilum
Lambeoceras lambii
Gonioceras anceps
Hoeloceras muroni
Cyrtonybyoceras haesitans
Adamsoceras oelandicum
Sibumasuoceras langkawiense
Langgunites mucronulatus
Destombesiceras zagorense
Nilssonoceras latisiphonatum
Archigeisonoceras repplingense
Tajaroceras wardae
Polymeres demetarum
Buttsoceras novemexicanum
Lituites lituus
Cyclolituites lynnensis
Ancistroceras ristnensis
Rhynchorthoceras conicum
Sinoceras fenxiangense
Sinoceras chinense
Protocycloceras lamarcki
Slemmestadoceras attavus
Bactroceras mourguesi
Bactroceras sandbergeri
Pachendoceras huzzahense
Rioceras escandei
Metabaltoceras fusiforme
Novacaroceras endogastrum
Palaeoceras mutabile
Balkoceras gracile
Sinoeremoceras sp.
Physalactinoceras bullatum
Plectronoceras cambria
490
480
470
460
450
440

Fig. S15. Full MCC tree of the CtIc analysis.

This analysis excluded species with high proportions of missing data. Numbers at nodes represent posterior probabilities. Colored boxes represent orders. Some species were recovered as sampled ancestors, represented here by black dots.

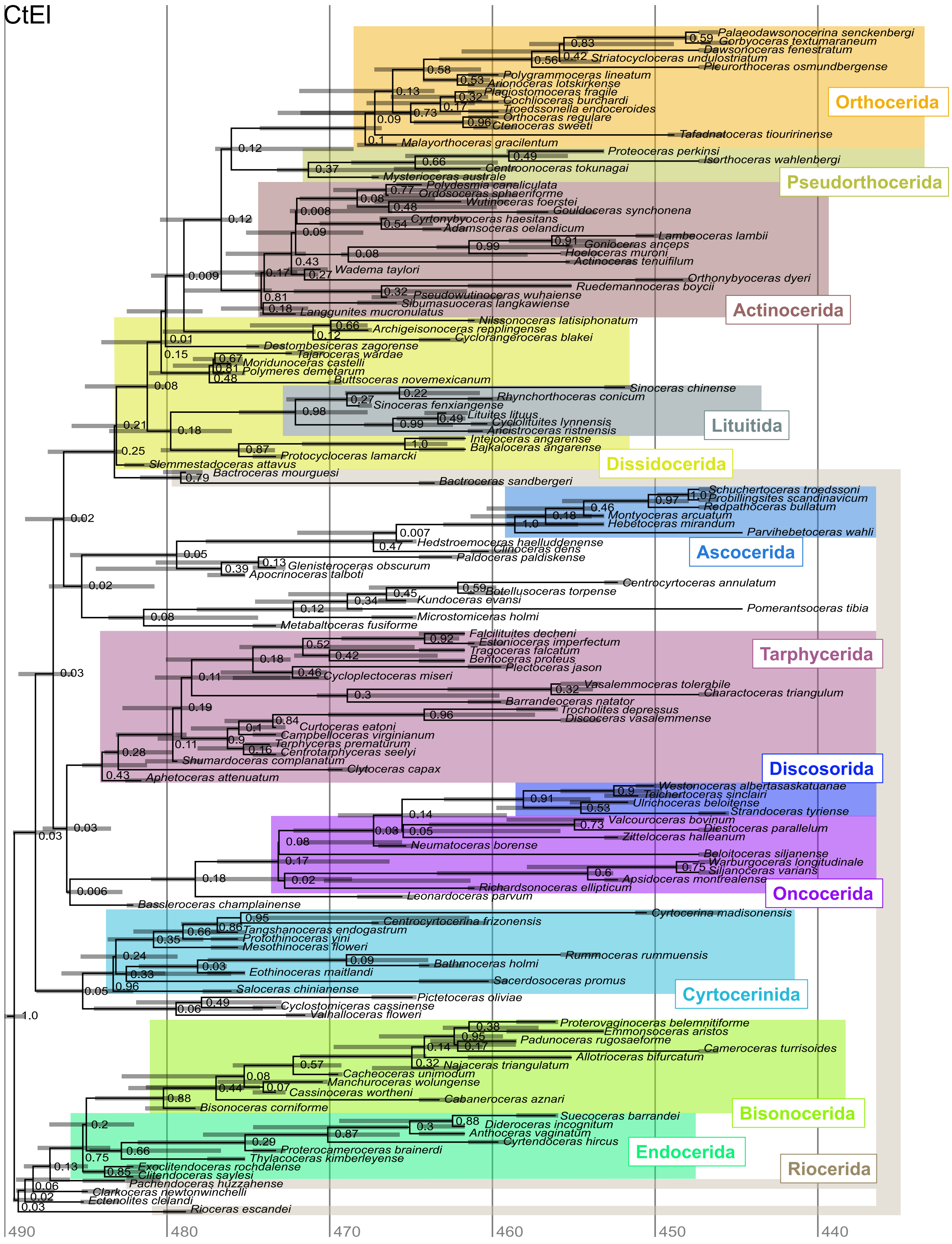
CtEI
Palaeodawsonocerina senckenbergi
Gorbyoceras textumaraneum
Dawsonoceras fenestratum
Striatocycloceras undulostriatum
Pleurorthoceras osmundbergense
Polygrammoceras lineatum
Arionoceras lotskirkense
Plagiostomoceras fragile
Cochlioceras burchardi
Troedssonella endoceroides
Orthoceras regulare
Ctenoceras sweeti
Tafadnatoceras tiouririnense
Malayorthoceras gracilentum
Orthocerida
Proteoceras perkinsi
Isorthoceras wahlenbergi
Centroonoceras tokunagai
Mysterioceras australe
Pseudorthocerida
Polydesmia canaliculata
Ordosoceras sphaeriforme
Wutinoceras foerstei
Gouldoceras synchonena
Cyrtonybyoceras haesitans
Adamsoceras oelandicum
Lambeoceras lambii
Gonioceras anceps
Hoeloceras muroni
Actinoceras tenuifilum
Wadema taylori
Orthonybyoceras dyeri
Ruedemannoceras boycii
Pseudowutinoceras wuhaiense
Sibumasuoceras langkawiense
Langgunites mucronulatus
Actinocerida
Nilssonoceras latisiphonatum
Archigeisonoceras repplingense
Cycloringeroceras blakei
Destombesiceras zagorense
Tajaroceras wardae
Moridunoceras castelli
Polymeres demetarum
Buttsoceras novemexicanum
Sinoceras chinense
Rhynchorthoceras conicum
Sinoceras fenxiangense
Lituites lituus
Cyclolituites lynnensis
Ancistroceras ristnensis
Lituitida
Intejoceras angarense
Bajkaloceras angarense
Protocycloceras lamarcki
Dissidocerida
Slemmestadoceras attavus
Bactroceras mourguesi
Bactroceras sandbergeri
Schuchertoceras troedssoni
Probillingsites scandinavicum
Redpathoceras bullatum
Montyoceras arcuatum
Hebetoceras mirandum
Parvihebetoceras wahli
Hedstroemoceras haelluddenense
Clinoceras dens
Paldoceras paldiskense
Ascocerida
Glenisteroceras obscurum
Apocrinoceras talboti
Centrocyrtoceras annulatum
Botellusoceras torpense
Kundoceras evansi
Pomerantsoceras tibia
Microstomiceras holmi
Metabaltoceras fusiforme
Falcilituites decheni
Estonioceras imperfectum
Tragoceras falcatum
Bentoceras proteus
Plectoceras jason
Tarphycerida
Cycloplectoceras miseri
Vasalemmoceras tolerabile
Charactoceras triangulum
Barrandeoceras natator
Trocholites depressus
Discoceras vasalemmense
Curtoceras eatoni
Campbelloceras virginianum
Tarphyceras prematurum
Centrotarphyceras seelyi
Shumardoceras complanatum
Clytoceras capax
Aphetoceras attenuatum
Discosorida
Westonoceras albertasaskatuanae
Teichertoceras sinclairi
Ulrichoceras beloitense
Strandoceras tyriense
Valcouroceras bovinum
Diestoceras parallelum
Zitteloceras halleanum
Neumatoceras borense
Beloitoceras siljanense
Warburgoceras longitudinale
Siljanoceras varians
Apsidoceras montrealense
Richardsonoceras ellipticum
Oncocerida
Leonardoceras parvum
Bassleroceras champlainense
Cyrtocerina madisonensis
Centrocyrtocerina frizonensis
Tangshanoceras endogastrum
Protothinoceras yini
Mesothinoceras floweri
Rummoceras rummuensis
Bathmoceras holmi
Eothinoceras maitlandi
Sacerdosoceras promus
Saloceras chinianense
Cyrtocerinida
Pictetoceras oliviae
Cyclostomiceras cassinense
Valhalloceras floweri
Proterovaginoceras belemnitiforme
Emmonsoceras aristos
Padunoceras rugosaeforme
Cameroceras turrisoides
Allotrioceras bifurcatum
Najaceras triangulatum
Cacheoceras unimodum
Manchuroceras wolungense
Cassinoceras wortheni
Cabaneroceras aznari
Bisonoceras corniforme
Bisonocerida
Suecoceras barrandei
Dideroceras incognitum
Anthoceras vaginatum
Cyrtendoceras hircus
Proterocameroceras brainerdi
Thylacoceras kimberleyense
Exoclitendoceras rochdalense
Clitendoceras saylesi
Pachendoceras huzzahense
Endocerida
Riocerida
Clarkoceras newtonwinchelli
Ectenolites clelandi
Rioceras escandei
490
480
470
460
450
440

Fig. S16. Full MCC tree of the CtEl analysis.

This analysis excluded a large number of early species, i.e., representatives of the Ellesmerocerida, Yanhecerida and Protactinocerida. Numbers at nodes represent posterior probabilities. Colored boxes represent orders. Some species were recovered as sampled ancestors, represented here by black dots.

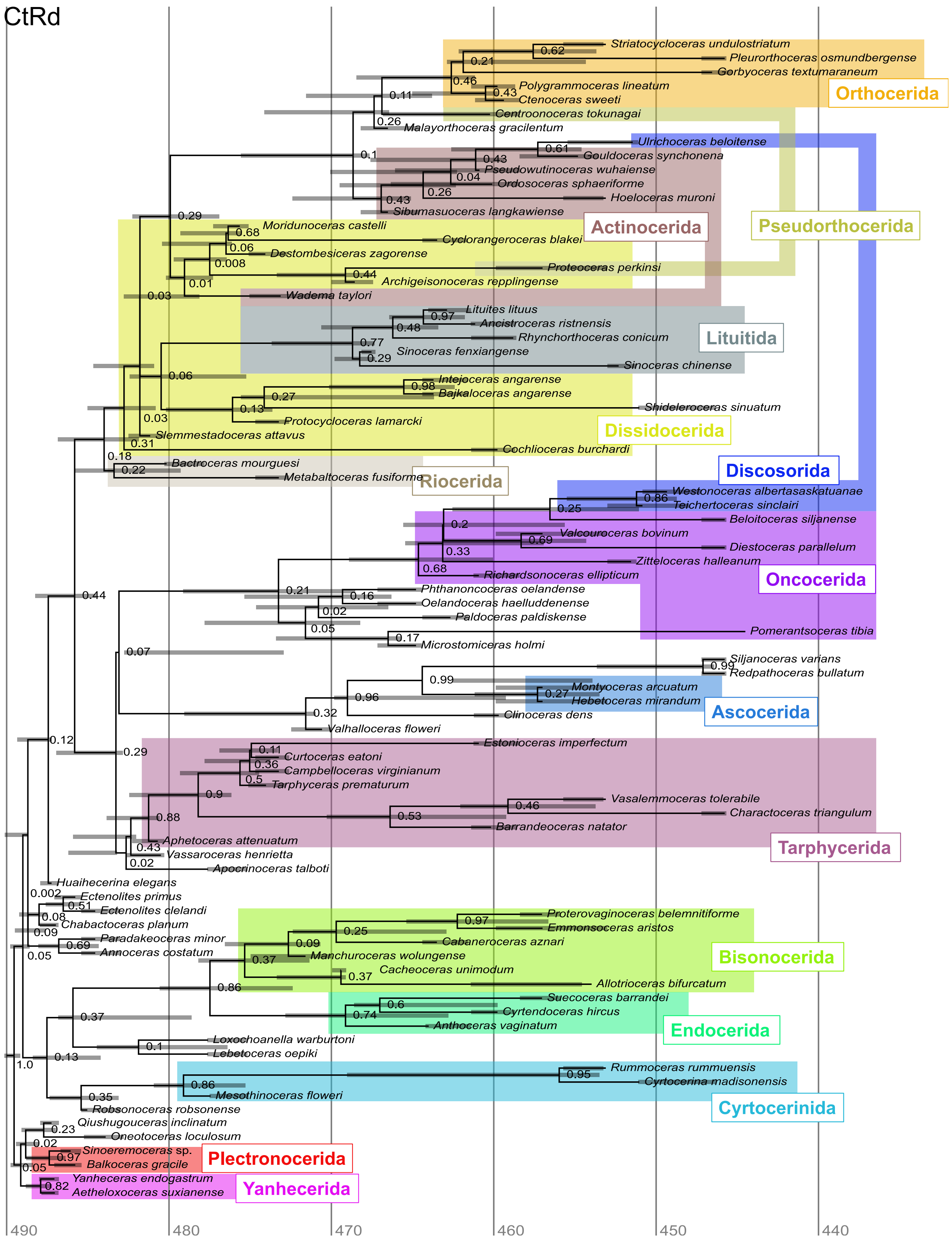
CtRd
Striatocycloceras undulostriatum
Pleurorthoceras osmundbergense
Gorbyoceras textumaraneum
Polygrammoceras lineatum
Ctenoceras sweeti
Centroonoceras tokunagai
Malayorthoceras gracilentum
Orthocerida
Ulrichoceras beloitense
Gouldoceras synchonena
Pseudowutinoceras wuhaiense
Ordosoceras sphaeriforme
Hoeloceras muroni
Sibumasuoceras langkawiense
Actinocerida
Pseudorthocerida
Moridunoceras castelli
Cyclorangeroceras blakei
Destombesiceras zagorense
Proteoceras perkinsi
Archigeisonoceras repplingense
Wadema taylori
Lituites lituus
Ancistroceras ristnensis
Rhynchorthoceras conicum
Sinoceras fenxiangense
Sinoceras chinense
Lituitida
Intejoceras angarense
Bajkaloceras angarense
Shideleroceras sinuatum
Protocycloceras lamarcki
Slemmestadoceras attavus
Dissidocerida
Cochlioceras burchardi
Bactroceras mourguesi
Metabaltoceras fusiforme
Riocerida
Discosorida
Westonoceras albertasaskatuanae
Teichertoceras sinclairi
Beloitoceras siljanense
Valcouroceras bovinum
Diestoceras parallelum
Zitteloceras halleanum
Richardsonoceras ellipticum
Oncocerida
Phthanonceras oelandense
Oelandoceras haelluddenense
Paldoceras paldiskense
Pomerantsoceras tibia
Microstomiceras holmi
Siljanoceras varians
Redpathoceras bullatum
Montyoceras arcuatum
Hebetoceras mirandum
Clinoceras dens
Ascocerida
Valhalloceras floweri
Estonioceras imperfectum
Curtoceras eatoni
Campbelloceras virginianum
Tarphyceras prematurum
Vasalemmoceras tolerabile
Charactoceras triangulum
Barrandeoceras natator
Aphetoceras attenuatum
Vassaroceras henrietta
Apocrinoceras talboti
Tarphycerida
Huaihecerina elegans
Ectenolites primus
Ectenolites clelandi
Chabactoceras planum
Paradakeoceras minor
Annoceras costatum
Proterovaginoceras belemnitiforme
Emmonsoceras aristos
Cabaneroceras aznari
Manchuroceras wolungense
Cacheoceras unimodum
Allotrioceras bifurcatum
Bisonocerida
Suecoceras barrandei
Cyrtendoceras hircus
Anthoceras vaginatum
Endocerida
Loxochoanella warburtoni
Lebetoceras oepiki
Rummoceras rummuensis
Cyrtocerina madisonensis
Mesothinoceras floweri
Cyrtocerinida
Robsonoceras robsonense
Qiushugouceras inclinatum
Oneotoceras loculosum
Sinoeremoceras sp.
Balkoceras gracile
Plectronocerida
Yanheceras endogastrum
Aetheloxoceras suxianense
Yanhecerida
490
480
470
460
450
440

Fig. S17. Full MCC tree of the CtRd analysis.

This analysis randomly excluded 50% of the species. Numbers at nodes represent posterior probabilities. Colored boxes represent orders. Some species were recovered as sampled ancestors, represented here by black dots.

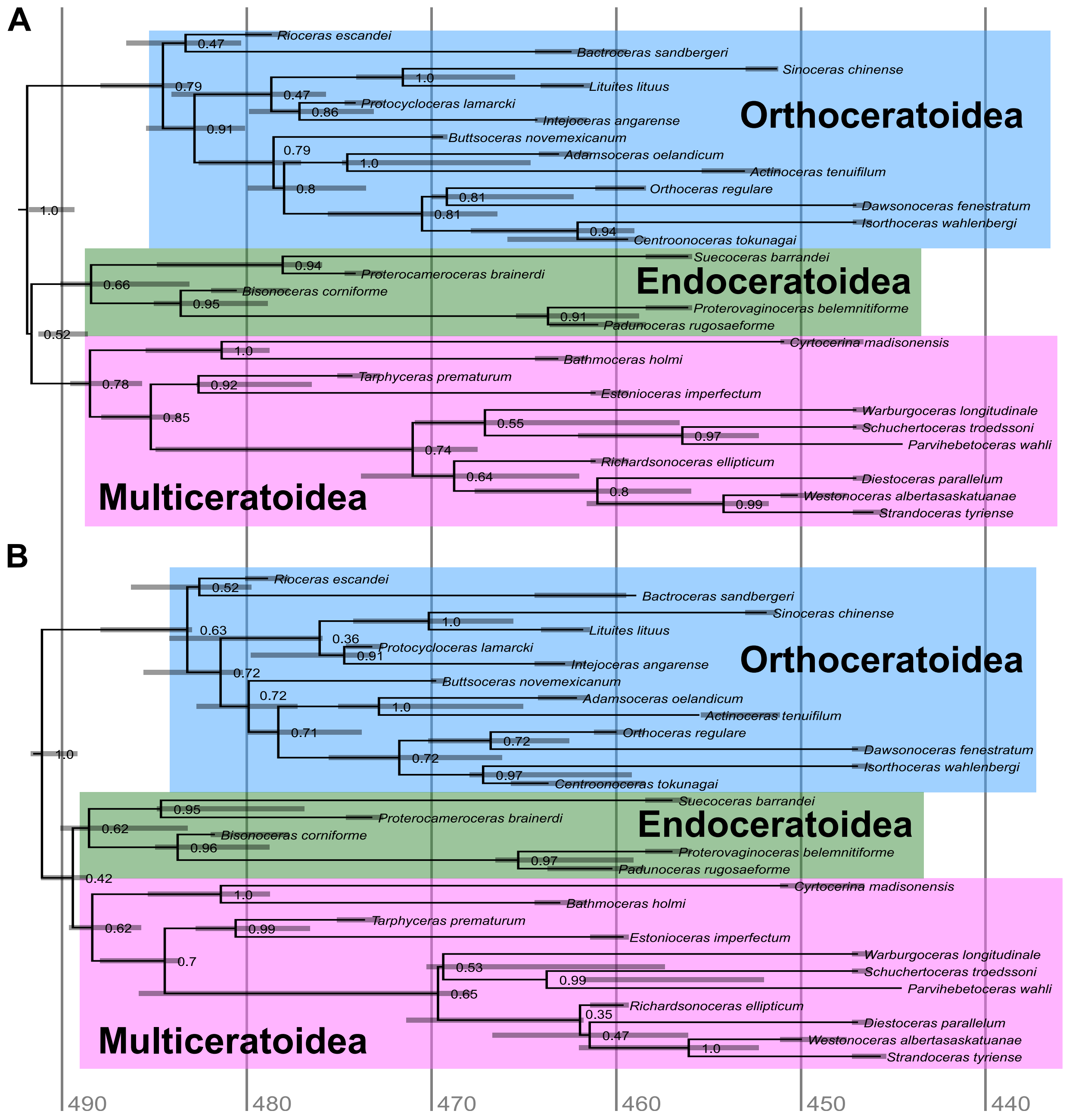

Fig. S18. Pruned MCC trees (1/6).

All trees in the posterior tree sample were pruned before reconstructing the MCC trees. The retained species were chosen according to a number of criteria (see Methods). Colored boxes represent subclasses. (**A**): CtCo, controversial characters excluded, 144 taxa pruned. (**B**): CoCo, controversial characters included, 144 taxa pruned.

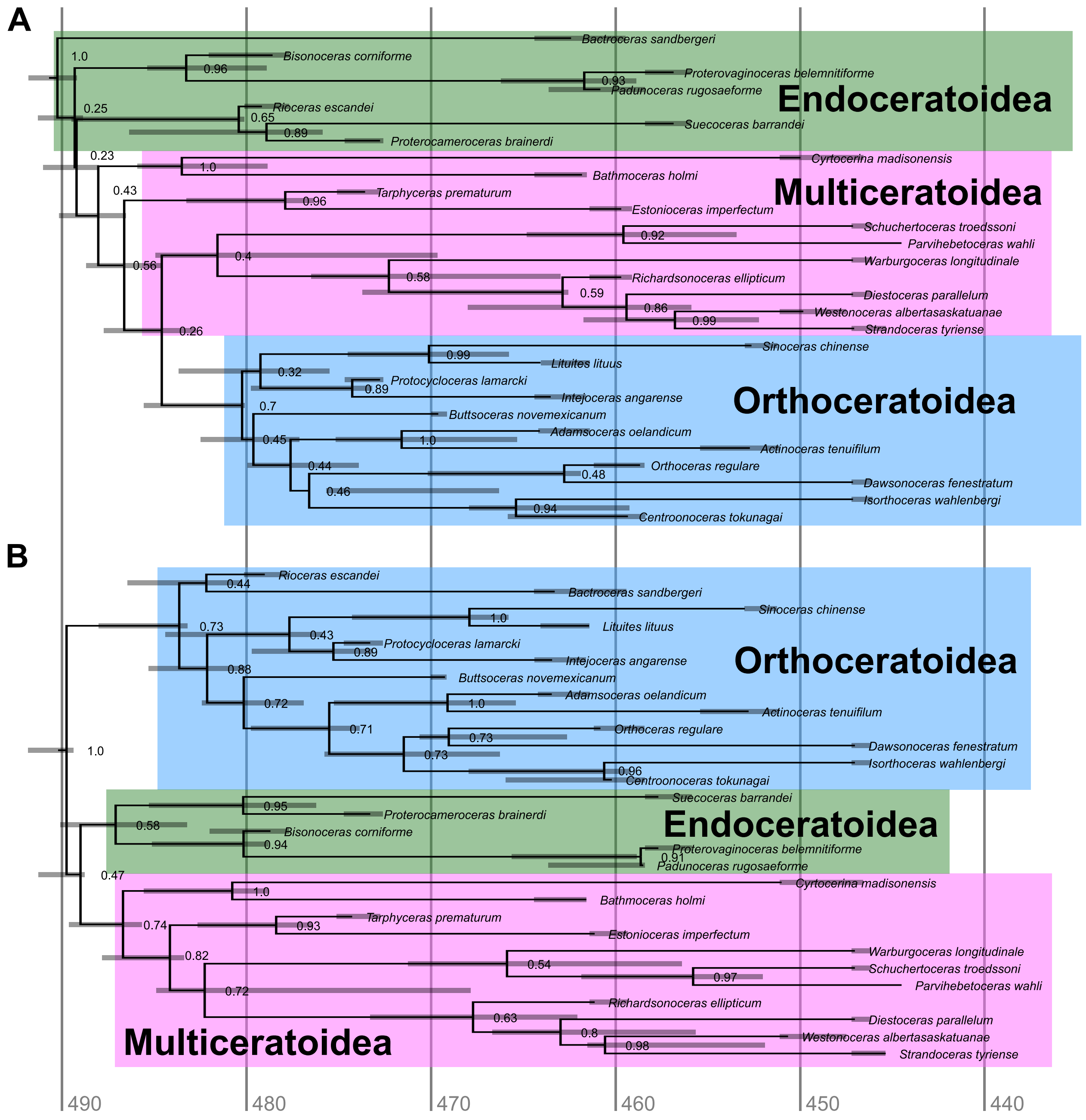

Fig. S19. Pruned MCC trees (2/6).

All trees in the posterior tree sample were pruned before reconstructing the MCC trees. The retained species were chosen according to a number of criteria (see Methods). Colored boxes represent subclasses. (**A**): IcCo, incomplete characters excluded, 144 taxa pruned. Note that the Endoceratoidea and Multiceratoidea are paraphyletic under this dataset. (**B**): AmCo, autapomorphic characters excluded, 144 taxa pruned.

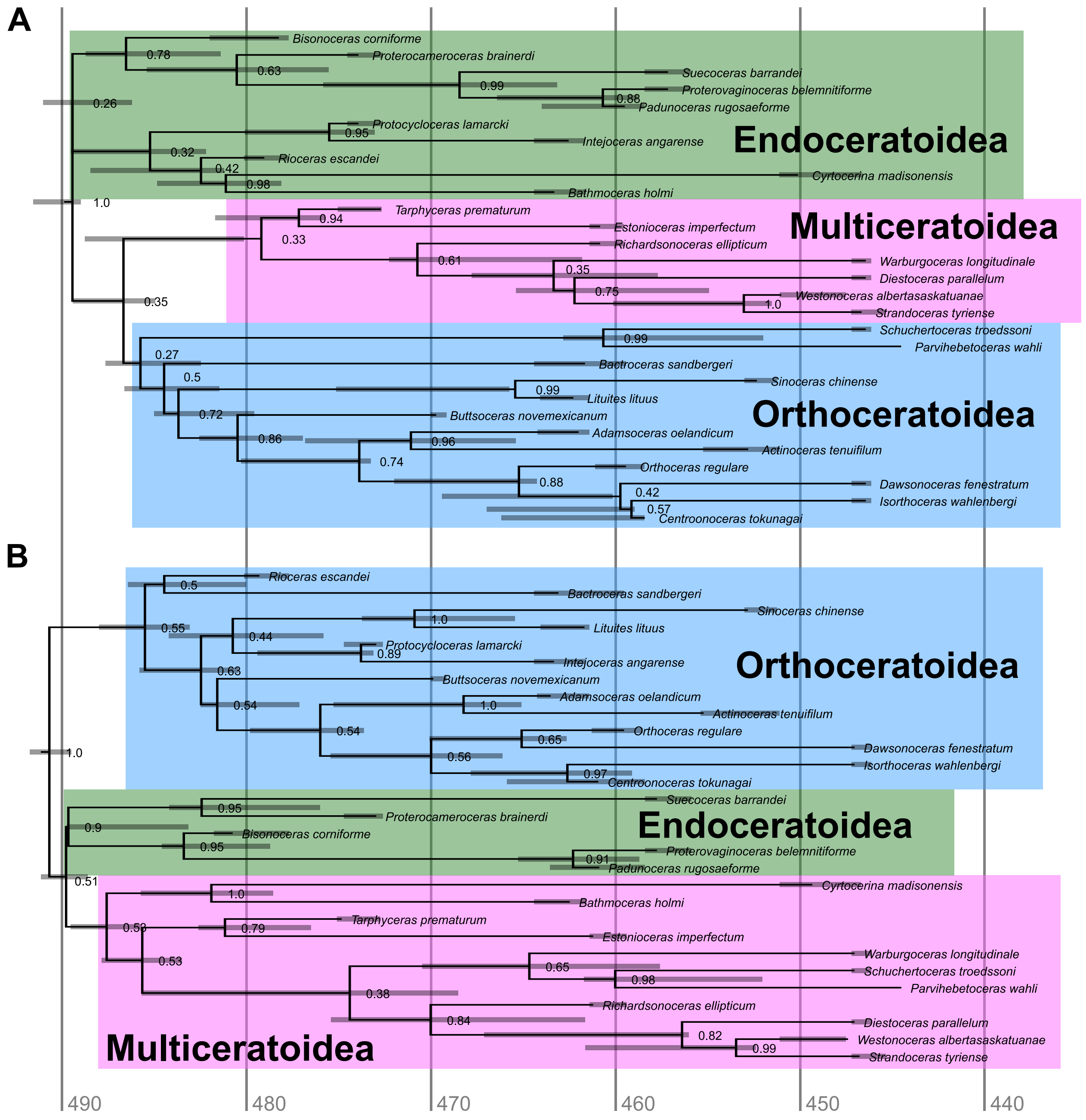

Fig. S20. Pruned MCC trees (3/6).

All trees in the posterior tree sample were pruned before reconstructing the MCC trees. The retained species were chosen according to a number of criteria (see Methods). Colored boxes represent subclasses. (**A**): IaCo, inapplicable characters excluded, 144 taxa pruned. (**B**): CrCo, speculatively scored connecting ring type, 144 taxa pruned.

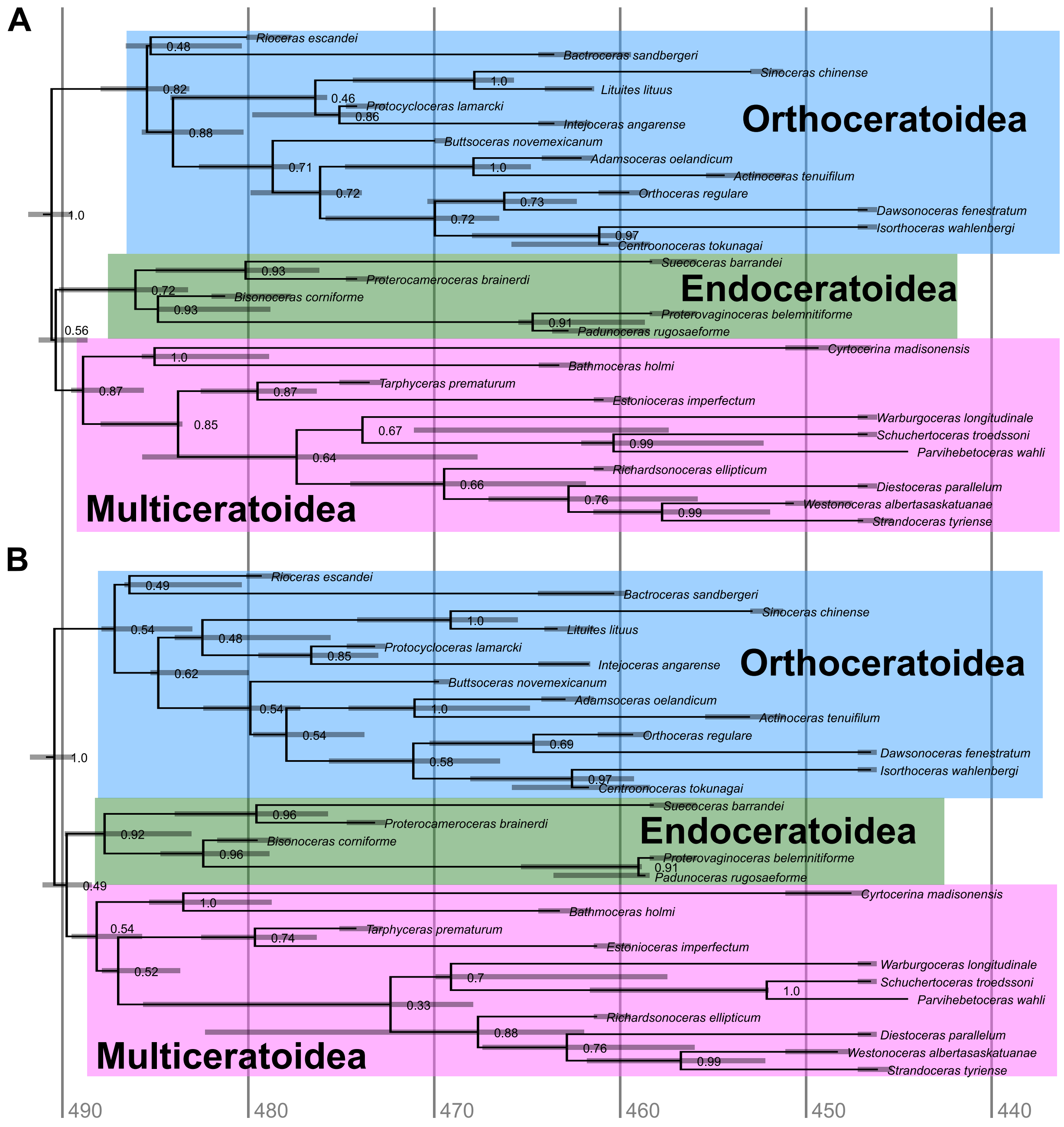

Fig. S21. Pruned MCC trees (4/6).

All trees in the posterior tree sample were pruned before reconstructing the MCC trees. The retained species were chosen according to a number of criteria (see Methods). Colored boxes represent subclasses. (**A**): MaCo, speculatively scored muscle attachment patterns, 144 taxa pruned. (**B**): CMCo, speculatively scored connecting ring and muscle attachments, 144 taxa pruned.

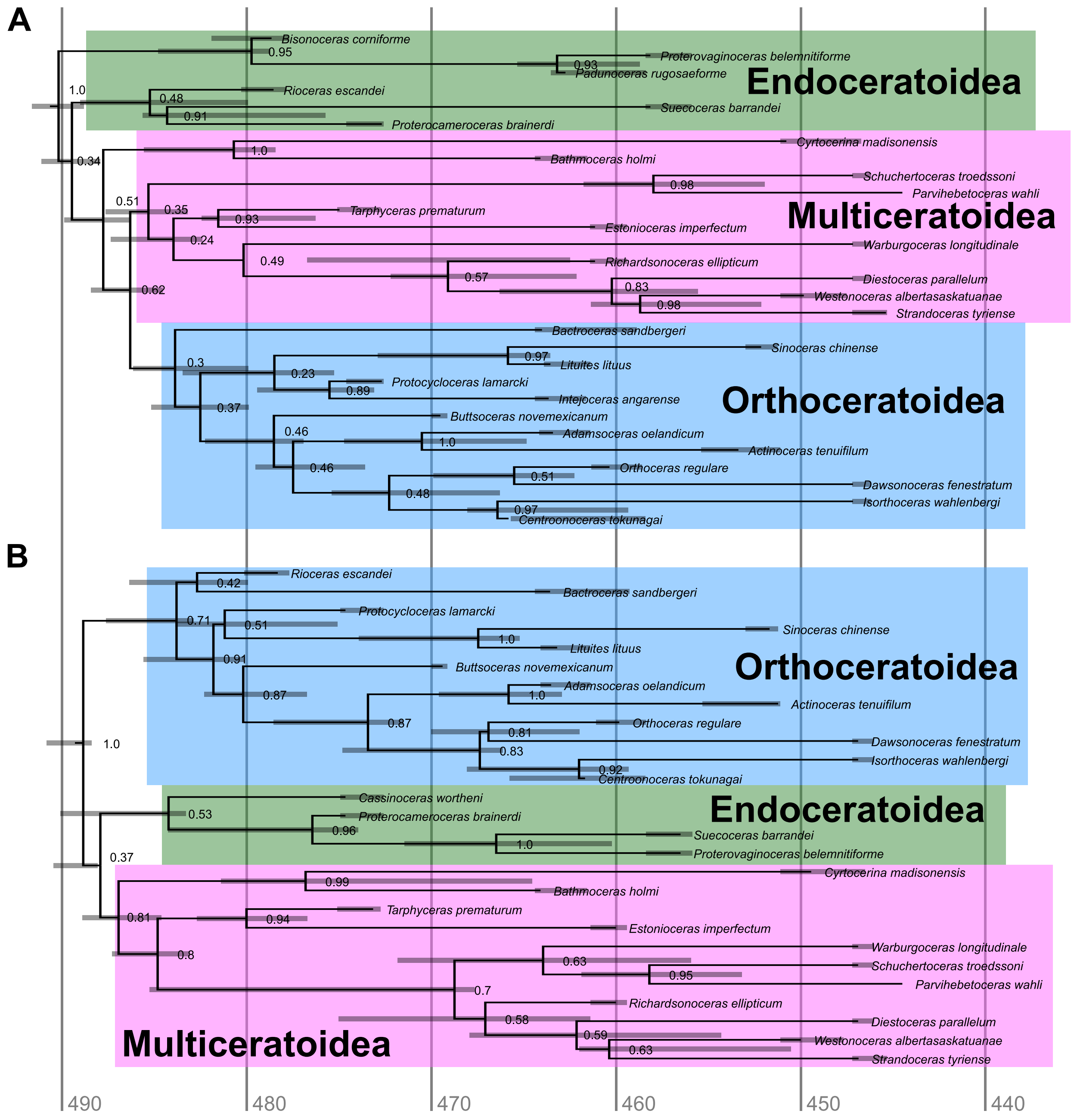

Fig. S22. Pruned MCC trees (5/6).

All trees in the posterior tree sample were pruned before reconstructing the MCC trees. The retained species were chosen according to a number of criteria (see Methods). Colored boxes represent subclasses. (**A**): CtDp, pseudoduplicate species excluded, 140 taxa pruned. Note that the Endoceratoidea and Multiceratoidea are paraphyletic under this dataset. (**B**): CtIc, Incomplete species excluded, 108 taxa pruned.

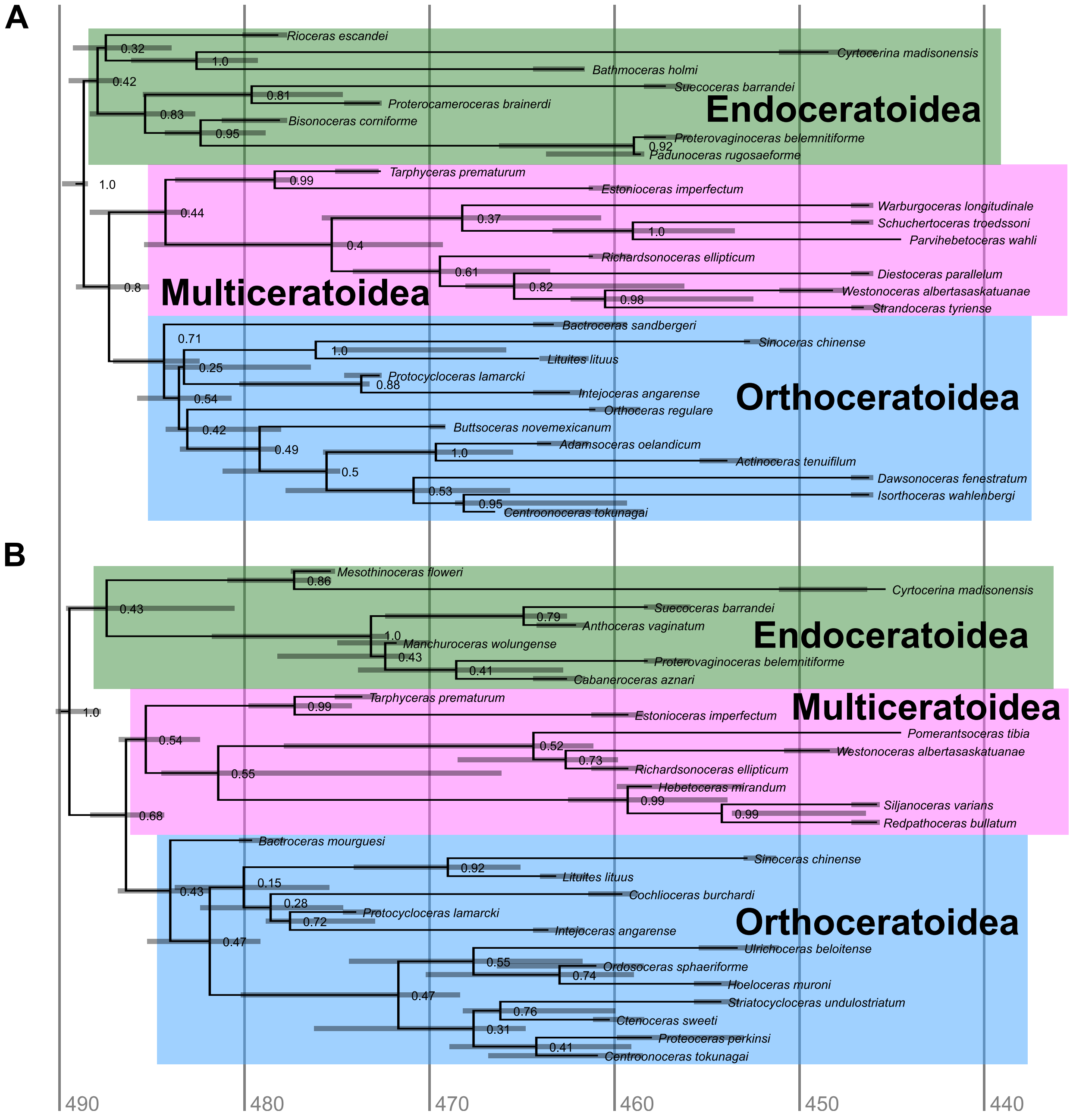

Fig. S23. Pruned MCC trees (6/6).

All trees in the posterior tree sample were pruned before reconstructing the MCC trees. The retained species were chosen according to a number of criteria (see Methods). Colored boxes represent subclasses. (**A**): CtEl, early species excluded, 111 taxa pruned. (**B**): CtRd, random species excluded, 59 taxa pruned.

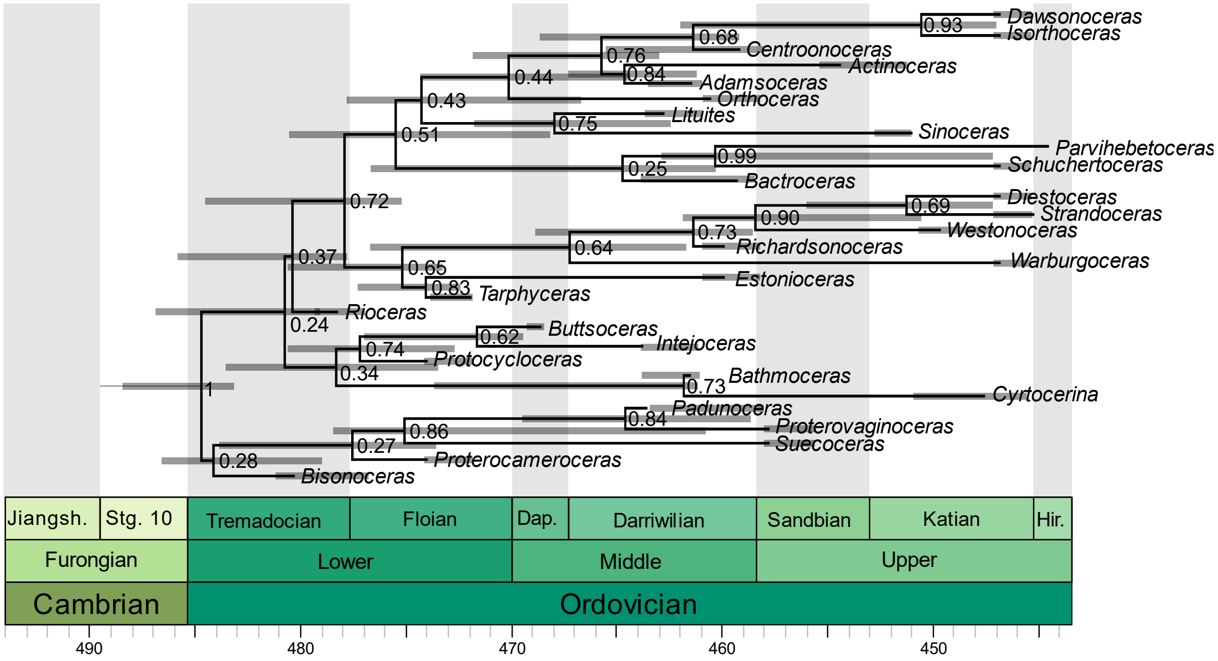

Fig. S24. MCC tree of an analysis containing the same taxa as the CtCo pruned MCC tree.

This analysis contained the same characters as the CtCo analysis. Note the alternative clade compositions and lowered posterior probabilities when compared to the pruned MCC tree of that analysis.

Table S1. Details of measurements and their abbreviations.

Abbreviation	Full name	Conch region
ah	Aperture height	Body chamber
al	Dorsal aponeurosis length	Body chamber
aw	Dorsal aponeurosis width	Body chamber
bh	Body chamber base height	Body chamber
bl	Body chamber length	Body chamber
br	Brim height	Siphuncle
ch	Conch height	Phragmocone
cl	Cameral length	Phragmocone
cw	Conch width	Phragmocone
fh	Septal foramen height	Siphuncle
fw	Septal foramen width	Siphuncle
ih	Initial chamber diameter (height)	Apex
il	Initial chamber length	Apex
is	Caecum (initial siphuncle) height	Apex
ld	Lateral lobe depth	Phragmocone
mb	Maximum body chamber height	Body chamber
mg	Position of maximum gibbosity	Body chamber
mh	Maximum conch height	Body chamber/phragmocone
mw	Maximum conch width	Body chamber/phragmocone
nl	Septal neck length	Siphuncle
pw	Minimum periphract width	Body chamber
rl	Ventral retractor length	Body chamber
rw	Ventral retractor width	Body chamber
sc	Septal concavity	Phragmocone
sh	Siphuncular segment height	Siphuncle
sl	Siphuncular segment length	Siphuncle
sv	Ventral distance to septal foramen	Siphuncle

Measurements are illustrated in Fig. 1 and Additional File 1: Fig. S1. More details can be found in the relevant character descriptions.

Table S2. Details of conch parameters and their abbreviations.

Abbreviation	Full name	Calculation
BNR	Brim-neck ratio	br/nl
CS	Contraction strength	(mb-ah)/(bl-mp)
CWI	Conch width index	ch/cw
ER	Expansion rate	See (*145*), or measured directly
ILI	Initial chamber length index	il/ih
ISI	Initial siphuncle (caecum) index	is/ih
MCS	Maximum conch size	log √(mh*mw)
PPI	Periphract width index	pw/bh
RAL	Relative dorsal aponeurosis length	al/bh
RAW	Relative dorsal aponeurosis width	aw/bh
RBL	Relative body chamber length	bl/bh
RCL	Relative cameral length	cl/ch
RHL	Relative hyponomic sinus length	hl/hw
RHW	Relative hyponomic sinus width	hw/cw
RLD	Relative lateral lobe depth	ld/ch
RRL	Relative ventral retractor length	rl/bh
RRW	Relative ventral retractor width	rw/bh
RSH	Relative septal foramen height	fh/ch
RSP	Relative septal foramen position	sv/(ch-fh)
RSS	Relative siphuncular shape	sh/fh
SCI	Septal concavity index	sc/ch
SCR	Siphuncle compression ratio	sh/sl
SNI	Septal neck index	nl/sl
SWI	Siphuncular width index	fh/fw

Conch parameters were calculated from at least two measurements to make them growth independent. More details can be found in the relevant character descriptions.

Table S3. Equivalent nodes in full and pruned MCC trees.

Dataset	FMCCT EqN mean pp	PMCCT EqN mean pp	mean difference	CN / TN
CtCo	0.397	0.817	0.420	1 / 28
CoCo	0.403	0.757	0.354	1 / 28
IcCo	0.316	0.683	0.367	14 / 28
AmCo	0.420	0.793	0.373	1 / 28
IaCo	0.367	0.694	0.327	3 / 28
CrCo	0.342	0.750	0.408	2 / 28
MaCo	0.437	0.811	0.374	2 / 28
CMCo	0.405	0.750	0.345	1 / 28
CtDp	0.340	0.670	0.329	6 / 28
CtIc	0.340	0.782	0.442	0 / 26
CtEl	0.293	0.712	0.420	9 / 28
CtRd	0.191	0.634	0.443	5 / 27

Only equivalent nodes are considered. Thus, the mean posterior probabilities of full MCC trees deviate slightly from the ones given in Additional file 1: Table S4. Abbreviations: FMCCT = full maximum clade credibility tree, PMCCT = pruned maximum clade credibility tree, EqN = equivalent node, CN = number of contradicting nodes, TN = total number of nodes.

Table S4. Tree similarities.

Dataset	Bp CtCo	Qu CtCo	QuN CtCo	Bp mean	Qu mean	QuN mean	PP mean
CtCo	-	-	-	0.459	0.845	0.779	0.404
CtCo*	**0.962**	**0.979**	**0.970**	0.827	**0.902**	0.860	0.817
CoCo	0.506	**0.946**	**0.923**	0.442	0.834	0.763	0.385
CoCo*	**0.962**	**0.979**	**0.970**	0.827	**0.901**	0.859	0.756
IcCo	0.447	0.699	0.570	0.402	0.733	0.619	0.390
IcCo*	0.731	0.788	0.697	0.737	0.812	0.731	0.682
AmCo	0.588	**0.942**	**0.917**	0.454	0.841	0.773	0.410
AmCo*	**0.962**	**0.979**	**0.970**	0.827	**0.901**	0.859	0.791
IaCo	0.282	0.678	0.540	0.327	0.724	0.606	0.336
IaCo*	0.577	0.731	0.616	0.566	0.751	0.644	0.695
CrCo	0.541	**0.953**	**0.933**	0.453	0.842	0.774	0.391
CrCo*	**0.962**	**0.979**	**0.970**	0.830	**0.903**	0.861	0.749
MaCo	0.488	**0.931**	**0.901**	0.440	0.836	0.766	0.393
MaCo*	**0.962**	**0.979**	**0.970**	0.830	**0.901**	0.859	0.812
CMCo	0.559	**0.952**	**0.931**	0.454	0.845	0.779	0.383
CMCo*	**0.962**	**0.979**	**0.970**	0.827	**0.902**	0.860	0.749
CtDp	0.452	0.728	0.611	0.413	0.745	0.636	0.387
CtDp*	0.731	0.859	0.799	0.747	0.872	0.817	0.670
CtIc	0.508	**0.925**	0.893	0.446	0.838	0.769	0.436
CtIc*	0.875	**0.982**	**0.974**	0.748	**0.900**	0.857	0.781
CtEl	0.438	0.787	0.696	0.418	0.810	0.729	0.410
CtEl*	0.731	0.872	0.817	0.721	0.877	0.824	0.710
CtRd	0.238	0.752	0.646	0.231	0.758	0.654	0.394
CtRd*	0.4	0.796	0.709	0.396	0.773	0.676	0.639
CtPr	0.385	0.665	0.521	-	-	-	0.644

Tree comparison metrics of the full MCC trees obtained from different runs and the pruned MCC trees (marked by *). The values represent bipartition and quartet agreement values. The trees were compared to the main analysis that excluded controversial characters (CtCo). In addition, the mean values of the comparisons with all other trees are shown. Lastly, for each tree, the mean posterior probabilities of all clades are given. For all metrics, a value of 1.0 equals identical trees. CtPr represents a separate analysis with the same taxa as in the pruned MCC tree of the CtCo analysis.

Additional file 2: Data S1. Species list (separate file)

This Excel file lists all species used in the analyses and includes a list of references. It furthermore lists corresponding stratigraphic horizons and their age ranges, and traditional views on the systematic assignment of each species (family and order level).

Additional file 3: Data S2. Measurements and conch parameters (separate file)

Here, all measurements and the corresponding conch parameters for continuous characters are listed. Note that the measurements do not always represent raw values but are proportionally adjusted in cases where not all measurements could be taken from the same specimen at the same position. One specimen is always taken as reference specimen, this specimen is explicitly cited with figure and repository number. Repository numbers printed in bold represent that the specimens have not been tracked, i.e., the corresponding collections may have been moved or reorganized. Underlined repository numbers indicate that the measurements have been taken from the literature. In cases, where additional specimens were used for measurements, the values are underlined. Values that carry some uncertainty, e.g., due to poor preservation, are printed in bold. Institutional abbreviations are listed at the bottom end.

Additional file 4: Data S3. Leaf stability indices and node distances (separate file)

This Excel file contains leaf stability indices and node distances obtained from the CtCo analysis. Abbreviations: END = Endoceratoidea; MUL = Multiceratoidea; ORTH = Orthoceratoidea; PEY = Plectronocerida/Yanhecerida/Ellesmerocerida; AgeMCC = age of the taxon in the CtCo full MCC tree; lsDif = leaf stability index (difference); lsEnt = leaf stability index (entropy); lsMax =leaf stability index (maximum); ct1mean = mean node distance to the closest tip in the MCC tree; ct2mean = mean node distance to the 2nd closest tip in the MCC tree; ; ct3mean = mean node distance to the 3rd closest tip in the MCC tree); ct1max = maximum node distance to the closest tip in the MCC tree; ct2max = maximum node distance to the 2nd closest tip in the MCC tree; ; ct3max = maximum node distance to the 3rd closest tip in the MCC tree.

Additional file 5: Data S4. Nexus files (separate file)

This zip-file contains all character matrices used for the analyses, including alternative character scorings.

Additional file 6: Data S5. BEAST files (separate file)

All xml-files used for the analyses and the corresponding summary trees are combined in a single zip-file.

Additional file 7: Data S6. R-script (separate file)

This file contains the script file that was used to calculate node distances of the closest tips.
